# Pathway analysis for intracellular *Porphyromonas gingivalis *using a strain ATCC 33277 specific database

**DOI:** 10.1186/1471-2180-9-185

**Published:** 2009-09-01

**Authors:** Erik L Hendrickson, Qiangwei Xia, Tiansong Wang, Richard J Lamont, Murray Hackett

**Affiliations:** 1 Department of Chemical Engineering, Box 355014 University of Washington, Seattle, WA 98195, USA; 2 Department of Microbiology, Box 357242 University of Washington, Seattle, WA 98195, USA; 3 Department of Oral Biology, University of Florida, Gainesville, FL 32610, USA; 4 University of Wisconsin-Madison, Department of Chemistry, Madison, WI 53706, USA

## Abstract

**Background:**

*Porphyromonas gingivalis *is a Gram-negative intracellular pathogen associated with periodontal disease. We have previously reported on whole-cell quantitative proteomic analyses to investigate the differential expression of virulence factors as the organism transitions from an extracellular to intracellular lifestyle. The original results with the invasive strain *P. gingivalis *ATCC 33277 were obtained using the genome sequence available at the time, strain W83 [GenBank: AE015924]. We present here a re-processed dataset using the recently published genome annotation specific for strain ATCC 33277 [GenBank: AP009380] and an analysis of differential abundance based on metabolic pathways rather than individual proteins.

**Results:**

Qualitative detection was observed for 1266 proteins using the strain ATCC 33277 annotation for 18 hour internalized *P. gingivalis *within human gingival epithelial cells and controls exposed to gingival cell culture medium, an improvement of 7% over the W83 annotation. Internalized cells showed increased abundance of proteins in the energy pathway from asparagine/aspartate amino acids to ATP. The pathway producing one short chain fatty acid, propionate, showed increased abundance, while that of another, butyrate, trended towards decreased abundance. The translational machinery, including ribosomal proteins and tRNA synthetases, showed a significant increase in protein relative abundance, as did proteins responsible for transcription.

**Conclusion:**

Use of the ATCC 33277 specific genome annotation resulted in improved proteome coverage with respect to the number of proteins observed both qualitatively in terms of protein identifications and quantitatively in terms of the number of calculated abundance ratios. Pathway analysis showed a significant increase in overall protein synthetic and transcriptional machinery in the absence of significant growth. These results suggest that the interior of host cells provides a more energy rich environment compared to the extracellular milieu. Shifts in the production of cytotoxic fatty acids by intracellular *P. gingivalis *may play a role in virulence. Moreover, despite extensive genomic re-arrangements between strains W83 and 33277, there is sufficient sequence similarity at the peptide level for proteomic abundance trends to be largely accurate when using the heterologous strain annotated genome as the reference for database searching.

## Background

The Gram-negative anaerobe *Porphyromonas gingivalis *is an important periodontal pathogen. Amongst the most common infections of humans, periodontal diseases are a group of inflammatory conditions that lead to the destruction of the supporting tissues of the teeth [1] and may be associated with serious systemic conditions, including coronary artery disease and preterm delivery of low birth weight infants [2]. *P. gingivalis *is a highly invasive intracellular oral pathogen [3] that enters gingival epithelial cells through manipulation of host cell signal transduction and remains resident in the perinuclear area for extended periods without causing host cell death [4]. The intracellular location appears to be an integral part of the organism's lifestyle and may contribute to persistence in the oral cavity. Epithelial cells can survive for prolonged periods post infection [5] and epithelial cells recovered from the oral cavity show high levels of intracellular *P. gingivalis *[6,7]. Intracellular *P. gingivalis *is also capable of spreading between host cells [8].

We have previously reported a whole-cell quantitative proteomic analysis of the change in *P. gingivalis *between extracellular and intracellular lifestyles [9]. *P. gingivalis *strain ATCC 33277 internalized within human gingival epithelial cells (GECs) was compared to strain ATCC 33277 exposed to gingival cell culture medium. The analysis focused on well-known or suspected virulence factors such as adhesins and proteases and employed the genome annotation of *P. gingivalis *strain W83. In order to be effective, quantitative proteomic analysis requires that mass spectometry results be matched to an annotated genome sequence to specifically identifiy the detected proteins. At the time, the only available whole genome annotation for *P. gingivalis *was that of strain W83 [10]. Recently, the whole genome sequence of *P. gingivalis *strain ATCC 33277 was published [11].

We re-analyzed the proteomics data using the *P. gingivalis *strain ATCC 33277 genome annotation. Use of the strain specific genome annotation increased the number of detected proteins as well as the sampling depth for detected proteins. As the quantitative accuracy of whole genome shotgun proteomics is dependent on sampling depth [12] the new analysis was expected to provide a more accurate representation of the changes in protein relative abundance between intracellular and extracellular lifestyles.

Given the prolonged periods of intracellular residence [4,5] it is likely that, in addition to changes in virulence factors, metabolic changes in response to the intracellular environment may play an inportant role in the intracellular lifestyle of *P. gingivalis*, including shifts in energy pathways and metabolic end products [13].

## Results and discussion

### Re-analysis using the *P. gingivalis *strain ATCC 33277 genome annotation

The proteomics data previously analyzed using the strain W83 genome annotation [GenBank: AE015924] [9] was recalculated employing the strain specific *P. gingivalis *strain ATCC 33277 annotation [GenBank: AP009380]. Accurately identifying a proteolytic fragment using mass spectrometry-based shotgun proteomics as coming from a particular protein requires matching the MS data to a protein sequence. Differences in amino acid sequence between the proteins expressed by strain ATCC 33277 and the protein sequences derived from the strain W83 genome annotation rendered many tryptic peptides from the whole cell digests employed unidentifiable in the original analysis [9]. Given that the quantitative power of the whole cell proteome analysis is dependent on the number of identified peptides [12,14], the new analysis was expected to give a more complete picture of the differential proteome, an expectation that proved accurate. In addition, some proteins in the strain ATCC 33277 genome are completely absent in the strain W83 genome and were thus qualitatively undetectable in the original analysis.

**Figure 1:**
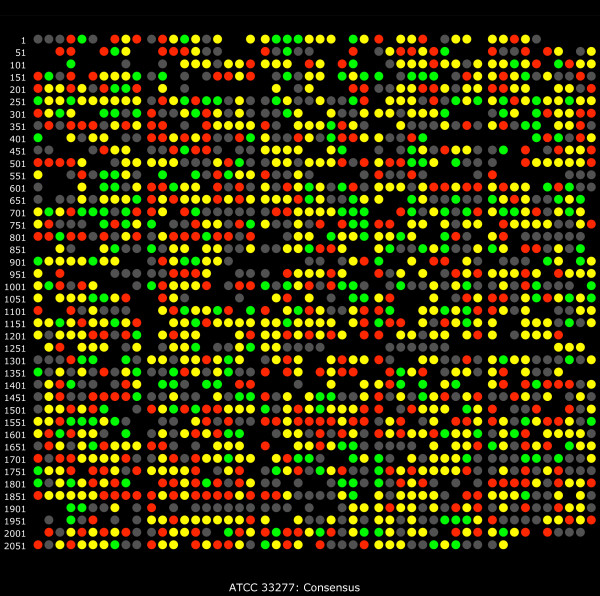
**Map of relative abundance trends based on the ATCC 33277 gene order and annotation**. This plot shows the entire set of consensus calls given in Additional file 1: Table S1 arranged by ascending PGN number [11], which follows the physical order of genes in the genome sequence. Color coding: red indicates increased relative protein abundance for internalized *P. gingivalis*, green decreased relative abundance, grey indicates qualitative non-detects and black indicates an unused ORF number.

Overall, 1266 proteins were detected with 396 over-expressed and 248 under-expressed proteins observed from internalized *P. gingivalis *cells compared to controls (Table 1). Statistics based on multiple hypothesis testing and abundance ratios for all detected proteins can be found in Additional file 1: Table S1, as well as pseudo M/A plots [15] of the entire dataset. The consensus assignment given in Additional file 1: Table S1 of increased or decreased abundance was based on two inputs, the *q*-values for comparisons between internalized *P. gingivalis *and gingival growth medium controls as determined by spectral counting and summed signal intensity from detected peptides that map to a specific ORF [9,14,15]. If one or the other of the spectral counting or protein intensity indicated a significant change (*q *≤ 0.01) and the other measure showed at least the same direction of change with a log_2 _ratio of 0.1 or better, then the consensus was considered changed in that direction, coded red for over-expression or green for under-expression. A simple "beads on a string" genomic map of the consensus calls is shown in Fig. 1.

**Table 1:** A comparison of the proteomics results employing either the W83 [10] or ATCC 33277 [11] genome annotations.

		ATCC 33277				
		Increased	Decreased	Unchanged	Not detected	Total
W83		396	248	622		1266
Increased	380	242	10	124	4	
Decreased	235	5	140	79	11	
Unchanged	570	93	75	345	57	
Not detected		56	23	74		
Total	1185					

The numbers of proteins showing increased, decreased or unchanged abundance in the internalized state for each analysis are given. Entries indicate the number of proteins from each category in one analysis that are assigned to the categories in the other analysis, including proteins that are not detected in a specific analysis.

Whole cell proteomics measurements of this type are noisy and the trade off between quantitative FDR (false discovery rate) and FNR (false negative rate) is made based on the informed judgment of the analyst, and often tends to be *ad hoc *and arbitrary in practice [9,14]. The *q*-value cut-off of 0.01 used here for statistical significance based on formal hypothesis testing was in good agreement with experimentally derived error distributions, as illustrated by the two pseudo M/A plots given in Additional file 1. The present findings serve to show the value of examining trends in groups of proteins, both as an end in itself with respect to biological questions and as feedback in the determination of proper cut-off values for the quantitative significance testing of individual proteins. As proteomics technology improves and it becomes economically feasible to run a greater number of independent cultures (biological replicates) than what was possible here, the overall noise issue in any one set of measurements will be less of a concern, and it will be easier to distinguish biological noise from deficiencies with respect to analytical repeatability, and thus identify biological trends that are truly significant rather than stochastically driven. Nonetheless, as in our previous work [9] the trends identified here are consistent with what we know about the behavior of the organism under intracellular conditions [3,9,16].

### Comparison between W83 and ATCC 33277 annotations for proteomics

As expected, the new analysis identified more proteins, 1266 proteins compared to 1185 in the previous analysis (Table 1). The number of proteins with statistically significant changes between internalized and medium incubated cells also increased, from 380 proteins with increased abundance to 396 proteins and from 235 proteins with decreased abundance to 248 proteins. This was a consequence of the higher number of proteolytic fragments detected across the proteome. However, there was a fairly large shift as to which proteins made the cut-off for statistically significant change: 168 proteins called unchanged in the W83 analysis now show statistically significant changes in the ATCC 33277-based analysis, while 203 proteins previously called significantly different no longer make the cut-off (Table 1), at *q *≤ 0.01. This is not surprising as values reasonably close to the cut-off point for significance would be expected to be very sensitive to changes in protein detection and sampling depth, with a small shift in the peptides involved in the calculations moving the protein over or under the significance cut-off point. A small number of proteins, 15, switched trend direction, moving from statistically significant increased or reduced abundance in internalized cells in the W83 analysis to the opposite trend in the ATCC 33277 analysis. The 15 proteins are listed in Table 2. In every case these 15 proteins showed inconsistency between two control cultures. In these cases the direction of change differed between the two controls with one control giving statistically significant change in one direction and the other giving change in the other direction but without making the statistical cut-off. Again, we saw shifts in borderline cases, in these 15 instances enough to shift the direction of abundance change. We also found that some proteins detected using the W83 genome annotation were no longer detected using the ATCC 33277 annotation. In most cases this was due to the presence of a second similar protein in the ATCC 33277 annotation, but not in the W83 annotation. Peptides that could not be unambiguously assigned to a single protein were not retained for the finished dataset given in Additional file 1: Table S1. The presence of the same peptide sequence in another protein eliminated the data from consideration both here and in the original W83-based analysis. Despite the shifts in assigned *q*-values and abundance ratio magnitudes as a consequence of the change in annotations, the abundance trends observed for *P. gingivalis *virulence factors did not differ greatly from those reported previously [9], except as noted in Table 2.

**Table 2:** The 15 proteins with opposite abundance trends.

PGN0148	conserved domain protein
PGN0152	immunoreactive 61 kDa antigen PG91
PGN0294	ragB lipoprotein RagB
PGN0302	rubrerythrin
PGN0503	mmdC methylmalonyl-CoA decarboxylase gamma subunit
PGN0678	thiL thiamine monophosphate kinase
PGN0914	peptidase M24 family
PGN1032	hypothetical protein PG_0914
PGN1403	acetylornithine aminotransferase putative
PGN1529	oxidoreductase putative
PGN1590	rplM ribosomal protein L13
PGN1830	TonB-dependent receptor putative
PGN1849	rplO ribosomal protein L15
PGN1904	hemagglutinin protein HagB
PGN2070	hypothetical protein PG_2204

Out of 1,113 detected (see Table 1) using both annotations, these 15 proteins showed inconsistent trends for significant (*q *≤ 0.01) abundance change depending on whether the W83 [10] or ATCC 33277 [11] genome annotations were used for database searching. The ORF numbers and descriptors given are those for ATCC 33277.

### Metabolic pathways differentially regulated in internalized *P. gingivalis*

The consensus assignments (see Additional file 1: Table S1) of differentially expressed proteins were used to populate metabolic pathways. The results were analyzed manually using the ATCC 33277 genome annotation [11]. In addition, an ontology analysis was done using DAVID (the Database for Annotation, Visualization and Integration Discovery) to identify over- or under-expressed ontology categories [17]. Putative changed categories were then checked manually. DAVID has proven to be useful for prokaryotes when compared with other ontology programs [18].

### Energy metabolism

**Figure 2:**
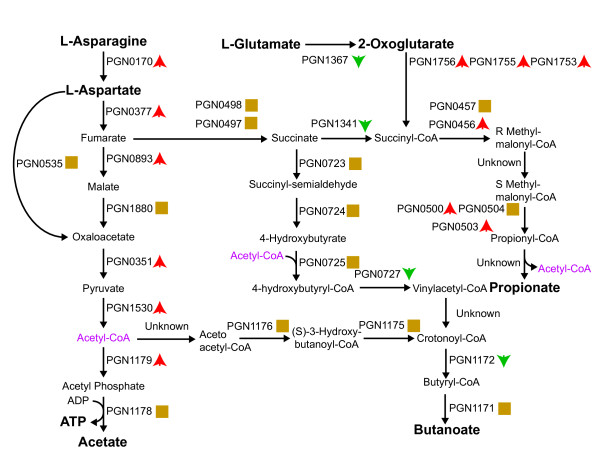
**Metabolic Map of Energy and Cytotoxin Production**. Proteins catalyzing each step are shown by their *P. gingivalis *PGN designation. Red up arrows indicate increased levels upon internalization, green down arrows decreased levels, and yellow squares no statistical change. Acetyl-CoA appears as a substrate and product at multiple points and is shown in purple. Metabolites and metabolic precursors discussed in the text are shown in bold.

*P. gingivalis *is an asaccharolytic bacterium and cannot survive on glucose or carbohydrates alone. While some genes for carbohydrate metabolism are found in the genome, *P. gingivalis *derives its energy from the metabolism of amino acids [11,13]. Takahashi and colleagues measured amino acid usage in culture and found that glutamate/glutamine and aspartate/asparagine were preferentially metabolized [13]. When grown on dipeptides of these substrates, *P. gingivalis *produced different amounts of metabolic byproducts. Importantly, aspartylaspartate produced significantly higher amounts of acetate, which is associated with ATP formation (Fig. 2 and Additional file 1: Table S1). Internalized *P. gingivalis *cells showed an increase in the energy pathway from aspartate/asparagine to acetate and energy (Fig. 2). The corollary of this trend is that the intracellular environment is energy rich for *P. gingivalis*. Interestingly, the protein that converts glutamate, the other favored amino acid, to 2-oxoglutarate (PGN1367, glutamate dehydrogenase) showed a decrease in abundance (Fig. 2). This may represent a preference for energy production in internalized cells or be part of a more general shift in the metabolic byproducts. We also observed a decrease in protein abundance of maltodextrin phosphorolase (PGN0733). Maltodextrin phospholase plays a role in digesting starches and, despite being an asaccharolytic organism, *P. gingivalis *may make some use of the starches available in the oral cavity, but restricts this activity after internalization.

### Cytotoxic byproducts

*P. gingivalis *metabolism produces several short chain fatty acid byproducts that are cytotoxic (Fig. 2) and has been found to shift production between these compounds depending on growth conditions [13]. We have found a general increase in the pathway from 2-oxoglutarate to the cytotoxin propionate while the proteins in the pathways for production of the cytotoxin butyrate showed unchanged or reduced expression (Fig. 2). This is consistent with hints that byproduct production shifts away from butyrate and towards propionate during *P. gingivalis *infections [19]. The results are the opposite of what would be expected from substrate studies. As mentioned previously, the proteomics shows an increase in the aspartate/asparagine pathway and a reduction in glutamate/glutamine. Culture growth studies found that *P. gingivalis *grown on aspartylaspartate had significantly more butyrate production than propionate compared to cultures grown on glutamylglutamate [13]. However, a recent flux balance model of *P. gingivalis *metabolism predicts that there is abundant flexibility in the production of butyrate, propionate and succinate with the metabolic routes to each being equivalent with respect to redox balancing and energy production [20]. Thus a shift towards propionate could be easily explained if it presented an advantage to internalized cells. In that regard, it has been shown that butyrate is a more potent apoptosis inducing agent than propionate [21]. Hence, the diminished production of butyrate by internalized *P. gingivalis *may contribute to the resistance of *P. gingivalis*-infected GECs to apoptotic cell death [22]. There is also the question of the reduced abundance of glutamate dehydrogenase (PGN1367), the protein that converts glutamate to 2-oxoglutarate (Fig. 2). If this is the primary substrate for propionate production it could limit that production even with increased abundance in the rest of the pathway. However, 2-oxoglutarate is a common metabolic intermediate and glutamate/glutamine may not be the only source of 2-oxoglutarate for propionate production. Even if it is the primary source, given the flexibility in byproduct production, a significant shift away from butyrate production from glutamate/glutamine to propionate production could still occur in the presence of an overall reduction in glutamate/glutamine usage. Interestingly, some similar shifts are seen between planktonic cells and biofilms of *P. gingivalis *strain W50. A mass spectrometry analysis of planktonic cells versus biofilm cells identified 81 proteins and found several energy metabolism proteins with significant differences between planktonic and biofilm lifestyles [23]. In biofilms fumarate reductase (PGN0497, 0498) had reduced abundance while oxaloacetate decarboxylase (PGN0351) had increased abundance similar to what we see in internalized cells (Fig. 2). Obviously, biofilms and the interior of GECs are different environments, and the energy metabolism protein glyceraldehyde-3-phosphate dehydrogenase (PGN0173) was increased in biofilms [23] relative to planktonic cells, while it is decreased in internalized cells relative to external controls. A comparison between the two conditions would really require the identification of more metabolic proteins from biofilm cells, but given the relevance of biofilm formation to *P. gingivalis *pathogenicity *in vivo *[24-26], the relation between biofilm conditions and internalized cells is an interesting one that we intend to pursue further at the whole proteome level.

### Translation machinery

Proteomics revealed a significant increase in proteins responsible for translation, including many of the ribosomal proteins (Table 3, 4 and 5, Additional file 1: Table S1). Increased abundance of ribosomal proteins is seen under conditions of increased growth rate in all domains of life [27-29]. However, we have found that internalized *P. gingivalis *maintain viability and replicate slowly within gingival epithelial cells [3]. Thus, an overall increase in protein expression due to increased energy production may be responsible for the increased abundance of translational machinery, more so than growth under these conditions.

**Table 3:** A list of detected proteins, by *P. gingivalis *PGN number [11], assigned to ribosomal proteins as determined using DAVID.

Increased **(32)**		Unchanged **(19)**		Decreased Levels **(1)**
PGN_0035	PGN_0167	PGN_0640	PGN_0965	PGN_0394
PGN_0188	PGN_0279	PGN_1572	PGN_1589	
PGN_0636	PGN_0639	PGN_1647	PGN_1648	
PGN_0641	PGN_0964	PGN_1698	PGN_1844	
PGN_1088	PGN_1219	PGN_1651	PGN_1852	
PGN_1573	PGN_1575	PGN_1853	PGN_1854	
PGN_1588	PGN_1590	PGN_1855	PGN_1861	
PGN_1832	PGN_1840	PGN_1863	PGN_1868	
PGN_1842	PGN_1843	PGN_1872	PGN_1890	
PGN_1849	PGN_1850	PGN_1891		
PGN_1856	PGN_1857			
PGN_1857	PGN_1858			
PGN_1860	PGN_1862			
PGN_1864	PGN_1865			
PGN_1866	PGN_1867			
PGN_1869	PGN_1871			

Proteins are indicated as increased, decreased or unchanged in abundance for internalized *P. gingivalis *versus external control cells. The totals for each category are given in parentheses.

**Table 4:** A list of detected proteins, by *P. gingivalis *PGN number [11], assigned to translation initiation, elongation and termination as determined using DAVID.

Increased **(8)**		Unchanged **(3)**		Decreased Levels **(0)**
PGN_0355	PGN_0963	PGN_0313	PGN_1014	
PGN_1405	PGN_1578	PGN_1244		
PGN_1587	PGN_1846			
PGN_1870	PGN_2022			

Proteins are listed by ORF number in the same manner as in Table 3.

**Table 5:** A list of detected proteins, by *P. gingivalis *PGN number [11], assigned to tRNA synthetases and transferases as determined using DAVID.

Increased **(16)**		Unchanged **(8)**		Decreased Levels **(3)**	
PGN_0209	PGN_0360	PGN_0137	PGN_0278	PGN_0266	PGN_0278
PGN_0365	PGN_0517	PGN_0281	PGN_0366	PGN_1157	
PGN_0543	PGN_0570	PGN_0569	PGN_0981		
PGN_0819	PGN_0962	PGN_1711	PGN_1883		
PGN_0987	PGN_1218				
PGN_1229	PGN_1381				
PGN_1805	PGN_1969				
PGN_2045	PGN_2060				

Proteins are listed by ORF number in the same manner as in Table 3.

### Transcription machinery

Most of the proteins responsible for transcription also showed increased abundance (Table 6, Additional file 1: Table S1). This is consistent with the overall increase in translational machinery as well as the larger number of proteins showing increased versus decreased abundance within gingival epithelial cells.

**Table 6:** A list of detected proteins, by *P. gingivalis *PGN number [11], assigned to transcription as determined using DAVID.

Increased **(7)**		Unchanged **(3)**		Decreased **(0)**
PGN_0423	PGN_0638	PGN_0792	PGN_1190	
PGN_1570	PGN_1571	PGN_1202		
PGN_1576	PGN_1578			
PGN_1630				

Proteins are listed by ORF number in the same manner as in Table 3.

## Conclusion

*P. gingivalis *is an opportunistic, intracellular pathogen that survives for extended periods of time within gingival epithelial cells without causing excessive harm to the host and thus provides a window into host cell adaptive responses by pathogens [3-5]. Re-analysis of whole cell proteomics data using the recently published strain specific genome annotation for ATCC 33277 allowed several novel conclusions. As expected, the strain specific annotation yielded better overall proteome coverage and sampling depth at the level of the number of proteins identified. However, most of the overall trends identified for major *P. gingivalis *virulence factors and other proteins using the W83 genome annotation remain unchanged, showing the viability of employing similar annotations when a strain specific sequence is unavailable. This observation is especially important for oral and gut microbes, where a rapidly increasing body of genomic and RNA-Seq data suggests that genomic re-arrangements in the absence of major changes in amino acid sequence for the expressed proteins may be a widespread occurrence. Although some differences in protein primary structure exist among *P. gingivalis *strains [30], the primary differences observed by Naito *et al. *are extensive genome re-arrangements [11]. The proteomic methods used here are highly sensitive to sequence similarity, but not at all to the order in which genes occur on the chromosome. However, the ways in which proteome data are interpreted in terms of operon and regulon structure are greatly influenced by the physical arrangement of the genome.

When the data were organized in terms of metabolic pathways the whole cell proteomics analysis revealed what appears to be a nutritionally rich intracellular environment for *P. gingivalis*. The energy metabolism pathway from the preferred amino acids aspartate/asparagine showed a significant increase. Transcription and translation proteins also showed significant increases, consistent with energy not being limiting. The production of cytotoxic metabolic byproducts also appears to shift in internalized cells, reducing production of butyrate and increasing production of propionate. This may be simply a byproduct of metabolic shifts, or it may play a role in *P. gingivalis *adaptive response to internalization.

## Methods

### Proteomic methods

The bacterial and gingival cell culturing, sample preparation, proteome extraction, proteolytic digestion, HPLC pre-fractionation, 2-D capillary HPLC [31,32], LTQ linear ion trap mass spectral data acquisition parameters, Sequest database searching [33], DTASelect [34]*in silico *assembly of the *P. gingivalis *proteome, protein relative abundance calculations, statistical methods and analytical validation for FDR and FNR [14] were all as published in the previous paper [9], with the following exceptions. The processing of the raw mass spectral data differs in this report due to the genome sequence annotation specific to strain ATCC 33277 [11], [GenBank: AP009380] which served as the basis for a new ORF database prepared by LANL (Los Alamos National Laboratory, Gary Xie, private communication). The custom database prepared by LANL was combined with reversed sequences from *P. gingivalis *ATCC 33277, human and bovine proteins as with our W83 database [GenBank: AE015924] described previously. The total size of the combined fasta file was 116 Mbytes. The estimated random qualitative FDR for peptide identifications based on the decoy strategy [35,36] was 3%.

### Assignment of ORF numbers

Additional file 1: Table S1 is arranged in ascending order by PGN numbers assigned for the experimental strain used here by Naito *et al*. [11]. They have been cross referenced to the W83 PG numbers originally assigned both by TIGR-CMR and LANL, where it was possible to do so. Certain ATCC 33277 genes do not have a counterpart in the older annotations based on the W83 genome, and will thus be blank in the summary table for PG numbers.

### DAVID

An overall list of detected proteins as well as lists of proteins that showed increased or decreased levels between internalized and gingival growth medium cultured cells were prepared using *Entrez *gene identifiers, as DAVID [17] does not recognize PGN numbers. Ontology analyses were then conducted using the DAVID functional annotation clustering feature with the default databases. Both increased and decreased protein level lists were analyzed using the overall list of detected proteins as the background. Potentially interesting clusters identified by DAVID were then examined manually.

## Abbreviations

ATCC: American Type Culture Collection; DAVID: Database for Annotation, Visualization and Integrated Discovery; FDR: false discovery rate; FNR: false negative rate; GEC: gingival epithelial cell; LANL: Los Alamos National Laboratory; MS: Mass spectrometry; ORF: open reading frame; TIGR-CMR: The Institute for Genomic Research Comprehensive Microbial Resource, now part of the J. Craig Venter Institute.

## Authors' contributions

QX calculated the protein abundance ratios and abundance change statistics. TW performed the mass spectrometry measurements. ELH performed the pathway and ontology analyses. MH and RJL conceived the experiments. ELH and MH wrote the manuscript. All authors read and approved the final manuscript.

## Supplementary Material

Additional file 1 **This file contains explanatory notes, two diagnostic pseudo M/A plots and Table S1, a summary of all the relative abundance ratios for internalized/control *P. gingivalis *mentioned in this report**. Prior to permanent archiving at LANL with the raw mass spectral data, summaries of the ATCC 33277-based protein identifications in the form of *DTASelect filter.txt *files will be available on a University of Washington server http://depts.washington.edu/mhlab/, rather than on the *BMC Microbiology *web site due to their large size. Request a password from the corresponding author. These files include details such as SEQUEST scores, peptide sequence, percentage of peptide coverage by observed ions in the CID spectrum, spectral counts, and other information described in the headers accompanying the filter files. More detail regarding the type of information contained in the filter files can be found in Tabb *et al. *[34]. Click here for file
