# Supplementary material for: Pathway analysis for intracellular Porphyromonas gingivalis using a strain ATCC 33277 specific database
Source: BMC Microbiol. 2009 Sep 1;9:185. doi: 10.1186/1471-2180-9-185 (PMC2753363; doi:10.1186/1471-2180-9-185)
Supplement: Additional file 1 — This file contains explanatory notes, two diagnostic pseudo M/A plots and Table S1, a summary of all the relative abundance ratios for internalized/control P. gingivalis mentioned in this report. Prior to permanent archiving at LANL with the raw mass spectral data, summaries of the ATCC 33277-based protein identifications in the form of DTASelect filter.txt files will be available on a University of Washington server http://depts.washington.edu/mhlab/, rather than on the BMC Microbiology web site due to their large size. Request a password from the corresponding author. These files include details such as SEQUEST scores, peptide sequence, percentage of peptide coverage by observed ions in the CID spectrum, spectral counts, and other information described in the headers accompanying the filter files. More detail regarding the type of information contained in the filter files can be found in Tabb et al. [34]. [file 1471-2180-9-185-S1.pdf]

Explanatory Notes, pseudo M/A plots, and Data Table ST1, Additional Material for Hendrickson *et al.*

The data table lists our entire set of results calculated from the raw mass spectral data first described in Xia *et al.*, *Proteomics* 2007, **7**: 4323-4337. The data were recalculated based on a search of annotated ORFs from the ATCC 33277 annotation of Naito *et al.*, *DNA Res* 2008, **15**:215-225, as described in the main text. The entire dataset is shown in the form of two pseudo M/A plots that precede Table ST1 below. These additional figures look very similar to those originally published using the W83 annotation, but we include them here as additional material for the sake of completeness. The color codes for the plots follow the data table, with the exception that proteins lacking any significant abundance change are coded black. Following our normal practice, a LOWESS (locally weighted scatter plot smoothing) curve fit to the scatter about zero abundance change, based on replicate control analyses, is superimposed over the plot, following the method of Cleveland, *Amer Statistician* 1981, **35**:54-54. For Table ST1, a key to the individual data fields is given below. The variable naming conventions follow those of Xia *et al.* in the original paper. The equations for the *t*-test, *G*-test and further details regarding their application can be found in Xia *et al.*, *Proteomics* 2007, **7:**2904-2919 and in the electronic supplement to Bosch *et al.*, *Proteomics* 2008, **8**:3494-3505. The forms given in Bosch *et al.* were the ones applied here, which differ from the equations originally described for the *P. gingivalis* internalization studies in Xia *et al.*, *Int Jour Mass spectrum* 2007, **259**:105-116.

Spectra Count Fields

ORF number. The ORF numbers from the three most common naming conventions are given. The field as a whole seems to be gravitating to the use of PGN numbers as assigned by Naito *et al.* for ATCC 33277 and related strains, thus we have ordered the entries by PGN number.

***q*-value.** A measure of false discovery rate, similar to a *p*-value, see Storey and Tibshirani, *Proc Natl Acad Sci USA* 2003, **100**:9440-9445. This q-value reflects the results of a global *G*-test over all replicates following the method popularized by Sokal and Rohlf referenced in the main text. It is worth noting that the global *G*-test as applied here requires that the data be pre-screened for instances where relative abundance trends for the same annotated ORF go in opposite directions from zero ($\log_2$ scale) within the same set of replicates. Such data are rare, but in the absence of such a pre-screen the *G*-test will not be sensitive to the direction of change from zero, thus leading to a call of significant change for data that should be properly coded as qualitative detection only, no abundance change. As noted in the main text, the *q* cut-off for all work reported in Table ST1 is 0.01.

PP(Norm). Internalized *P. gingivalis* normalized average total spectral counts over all replicates.

PPC(Norm). External control *P. gingivalis* normalized average total spectral counts over all replicates.

$\log_2$Ratio. $\log_2$ ratio of PP(Norm)/PPC(norm).

$\log_2$sum. Log_2 of the sum of PP(Norm) and PPC(norm).

Regulation. A simple color code is given, indicating the direction of abundance change. Red, increased abundance in the internalized *P. gingivalis* cells; green, decreased relative abundance; yellow, qualitative detection in the absence of statistically significant abundance change.

Protein Intensity Fields

***q*-value.** A measure of false discovery rate, similar to a *p*-value, see Storey and Tibshirani as noted above. This *q*-value reflects the results of a paired *t*-test over all replicates, according to the method described in Sokal and Rohlf, as given in the main text.

PP(Norm). Internalized *P. gingivalis* normalized average total signal intensity for all MS^1 ions over all replicates.

PPC(Norm). External control *P. gingivalis* normalized average total signal intensity for all MS^1 ions over all replicates.

$\log_2$Ratio. Log_2 ratio of PP(Norm)/PPC(norm).

$\log_2$sum. Log_2 of the sum of normalized average total intensities for PP(Norm) and PPC(norm).

Regulation. A simple color code indicating the direction of abundance change. Red, increased abundance in the internalized *P. gingivalis* cells; green, decreased relative abundance; yellow, qualitative detection in the absence of statistically significant abundance change.

Consensus.

Readers are urged to consider the spectral count and intensity data separately, but an overall assessment is provided. If all observed trends for a given protein were in the same direction and either spectral counts or intensity were significantly so, then the protein was labeled red or green, if all deviations from zero were greater than 0.1 $\log_2$ units. This rule was *ad hoc* and arbitrary, but yielded results that seemed to fit this dataset reasonably well.

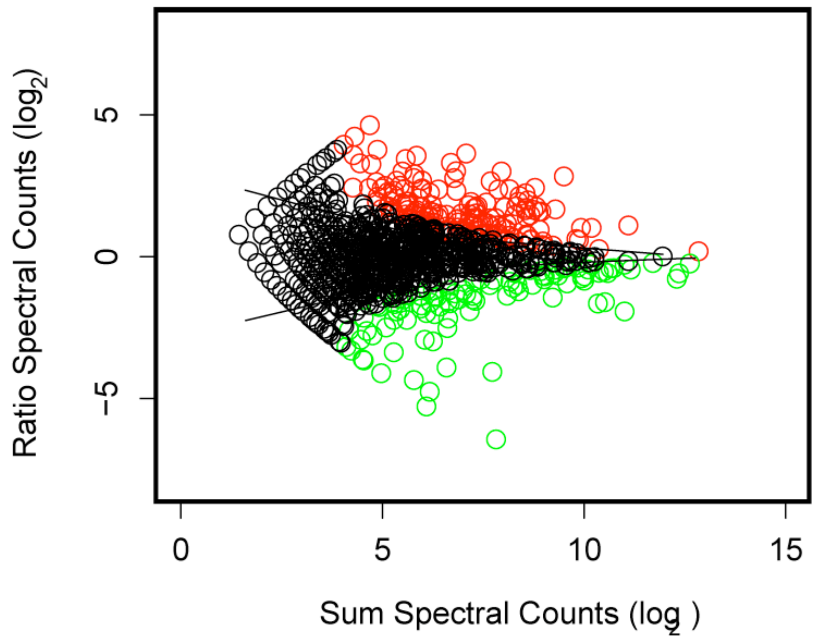

Pseudo M/A plot for the spectral counting data based on the ATCC 33277 annotation. All protein abundance ratios are internalized/control.

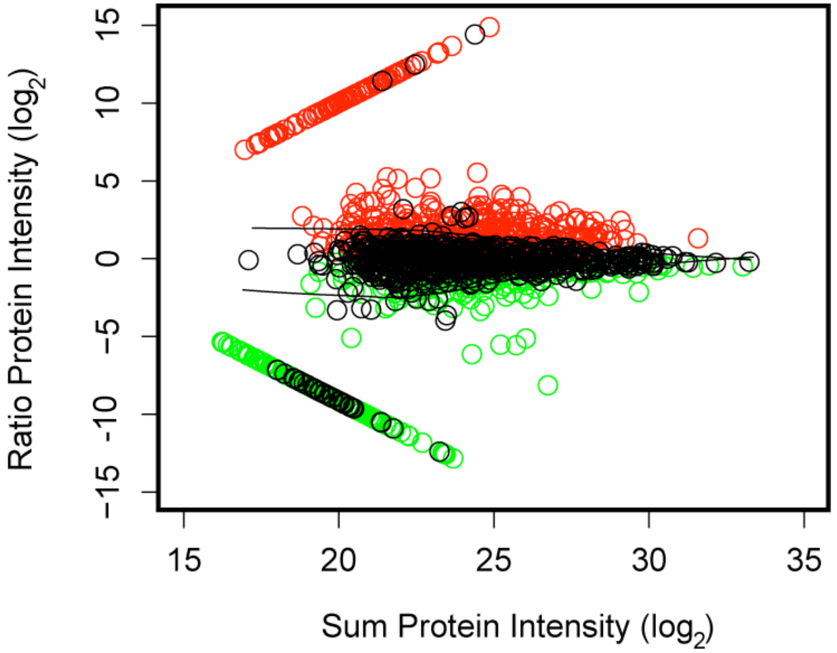

Pseudo M/A plot for the summed signal intensity data based on the ATCC 33277 annotation. All protein abundance ratios are internalized/control.

TIGR		PP PPC / Pg Spectral Count							PP PPC/ Pg Protein Intensity					33277
PGN	*LANL*	*q*-value	PP (Norm)	PPC (Norm)	$\log_2$Ratio	$\log_2$Sum	Regulation		*q*-value	PP (Norm)	PPC (Norm)	$\log_2$Ratio	$\log_2$Sum	Consensus
PG0004		2.98 E-1	13.7	8	0.7751	4.4390	Y	R	1.15 E-6	7.93 E+6	2.37 E+6	1.7408	23.2967	R
PGN0004	*PG0004*	*transcriptional regulator Sir2 family*												
PG0005		6.63 E-2	2.6	10	-1.9619	3.6516	Y	G	4.39 E-5	1.28 E+6	3.3 E+6	-1.3635	22.1298	G
PGN0005	*PG0005*	*conserved hypothetical protein*												
PG0010		5.31 E-2	9.4	2	2.2345	3.5125	Y	R	2.58 E-16	4.51 E+6	8.59 E+5	2.3926	22.3556	R
PGN0008	*PG0009*	*clpC ATP-dependent Clp protease ATP-binding subunit ClpC*												
PG0011		5.28 E-2	31.7	16	0.9846	5.5747	Y	Y	1.08 E-1	1.26 E+7	1.05 E+7	0.2626	24.4651	Y
PGN0009	*PG0010*	*glycosyl hydrolase family 3*												
PG0012		9.24 E-9	54.8	8.5	2.6876	5.9833	R	R	0	2.73 E+7	4.78 E+6	2.5157	24.9367	R
PGN0010	*PG0011*	*L-threonine-O-3-phosphate decarboxylase putative*												
PG0016		2.67 E-1	9.4	4.5	1.0646	3.7983	Y	R	6.34 E-7	2.22 E+6	6.91 E+5	1.6845	21.4735	R
PGN0012	*PG0013*	*sigma-54 dependent DNA-binding response regulator*												
PG0017		6.69 E-1	1.7	1.5	0.1901	1.6832	Y	G	0	1.85 E+3	5.11 E+5	-8.1093	18.9682	Y
PGN0013	*PG0014*	*sensor histidine kinase*												
PG0018		6.69 E-1	1.7	2	-0.2249	1.8919	Y	G	4.95 E-8	1.85 E+3	1.54 E+5	-6.3812	17.2521	G
PGN0014	*PG0015*	*hypothetical protein PG_0018*												
PG0020		6.97 E-1	3.4	3.5	-0.0323	2.7913	Y	Y	1.75 E-1	1.11 E+6	1.29 E+6	-0.2139	21.1945	Y
PGN0015	*PG0017*	*transcriptional regulator MarR family*												
PG0022		4.75 E-1	6.8	4.5	0.6052	3.5040	Y	Y	4.5 E-2	2.49 E+6	1.25 E+6	1.0013	21.8339	Y
PGN0017	*PG0019*	*sulfate permease family protein*												
PG0024		3.76 E-1	6.0	3	0.9975	3.1683	Y	Y	3.33 E-2	4.41 E+6	2.66 E+6	0.7305	22.7544	Y
PGN0020	*PG0020*	*DNA-binding protein putative*												
PG0025		6.97 E-1	3.4	3.5	-0.0323	2.7913	Y	Y	7.32 E-2	7.76 E+5	9.4 E+5	-0.2774	20.7106	Y
PGN0021	*PG0021*	*fumarylacetoacetate hydrolase family protein*												
PG0026		1.36 E-3	17.1	45	-1.3948	5.9568	G	G	6.45 E-4	1.15 E+7	3.55 E+7	-1.6204	25.4865	G
PGN0022	*PG0022*	*hypothetical protein PG_0026*												
PG0027		8.17 E-11	141.2	281.5	-0.9956	8.7234	G	G	1.5 E-8	8.52 E+7	1.65 E+8	-0.9567	27.9014	G
PGN0023	*PG0023*	*hypothetical protein PG_0027*												
PG0028		2.99 E-1	6.0	11	-0.8770	4.0866	Y	G	1.47 E-4	1.45 E+6	4.78 E+6	-1.7233	22.5698	G
PGN0024	*PG0024*	*ispF 2C-methyl-D-erythritol 24-cyclodiphosphate synthase*												
		4.4 E-1	1.7	3.5	-1.0323	2.3816	Y	Y	2.08 E-2	1.85 E+3	3.1 E+5	-7.3867	18.2490	Y
PGN0025														
PG0030		4.14 E-1	6.0	9.5	-0.6655	3.9532	Y	Y	4.25 E-3	3.9 E+6	2.15 E+6	0.8606	22.5266	Y
PGN0026	*PG0026*	*cytidine deaminase*												

TIGR	PGN	LANL	Description	PP PPC / Pg Spectral Count q-value	PP (Norm)	PPC (Norm)	log_2Ratio	log_2Sum	Regulation (Spectral Count)	Regulation (Protein Intensity)	PP PPC/ Pg Protein Intensity q-value	PP (Norm)	PPC (Norm)	log_2Ratio	log_2Sum	Consensus
	PGN0027			1.94 E-2	47.9	25	0.9386	6.1882	Y	Y	5.07 E-3	2.22 E+7	1.22 E+7	0.8604	25.0380	Y
PG0031	PGN0029	PG0027	hypothetical protein PG_0031	3.86 E-7	1.7	29.5	-4.1075	4.9640	G	G	3.46 E-10	1.85 E+3	1.35 E+7	-12.8324	23.6863	G
PG0032	PGN0030	PG0028	beta-mannosidase putative	3.05 E-1	6.8	12	-0.8099	4.2361	Y	Y	2.08 E-3	9.94 E+5	2.11 E+6	-1.0880	21.5672	Y
PG0033	PGN0031	PG0029	RmuC domain protein	6.69 E-1	3.4	3	0.1901	2.6832	Y	R	1.05 E-4	1.31 E+6	6.85 E+5	0.9334	20.9273	R
PG0034	PGN0033	PG0030	trx thioredoxin	6 E-1	81.3	76.5	0.0876	7.3018	Y	Y	9.4 E-2	1.45 E+8	1.17 E+8	0.3030	27.9661	Y
PG0035	PGN0034	PG0031	dnaE DNA polymerase III alpha subunit	3.56 E-2	7.7	20.5	-1.4125	4.8177	Y	Y	2.99 E-3	1.79 E+6	4.3 E+6	-1.2630	22.5391	Y
PG0037	PGN0035	PG0032	rplS ribosomal protein L19	2.1 E-7	52.2	10	2.3839	5.9587	R	R	0	4.45 E+7	8.57 E+6	2.3769	25.6615	R
PG0042	PGN0038	PG0035	glyA serine hydroxymethyltransferase	1.38 E-1	428.7	378	0.1815	9.6559	Y	Y	1.77 E-2	5.22 E+8	6.28 E+8	-0.2653	30.0994	Y
PG0043	PGN0039	PG0036	nahA beta-hexosaminidase	5.43 E-1	53.9	48.5	0.1525	6.6782	Y	Y	1.61 E-1	1.82 E+7	1.9 E+7	-0.0613	25.1489	Y
PG0045	PGN0041	PG0038	htpG heat shock protein HtpG	7.43 E-2	37.6	21.5	0.8083	5.8863	Y	Y	9.26 E-2	2.09 E+7	1.76 E+7	0.2515	25.1993	Y
PG0046	PGN0042	PG0040	cdsA phosphatidate cytidylyltransferase	6.69 E-1	3.4	3	0.1901	2.6832	Y	Y	4.47 E-2	6.51 E+5	4.6 E+5	0.5027	20.0837	Y
PG0047	PGN0043	PG0041	cell division protein FtsH putative	9.98 E-4	29.1	7.5	1.9557	5.1935	R	R	0	1.04 E+7	1.8 E+6	2.5308	23.5437	R
PG0048	PGN0044	PG0042	conserved hypothetical protein TIGR00092	2.15 E-1	8.6	3.5	1.2897	3.5917	Y	Y	1.43 E-1	4.02 E+6	2.81 E+6	0.5157	22.7024	Y
	PGN0053			4.28 E-2	157.4	118	0.4160	8.1056	Y	R	1.45 E-13	9.76 E+7	4.24 E+7	1.2013	27.0607	R
	PGN0054			4.27 E-4	106.1	56.5	0.9091	7.3452	R	R	2.58 E-16	3.95 E+7	1.33 E+7	1.5728	25.6526	R
	PGN0057			5.19 E-1	11.1	8.5	0.3881	4.2945	Y	R	2.18 E-8	3.48 E+6	8.49 E+5	2.0347	22.0457	R
	PGN0058			9.87 E-3	6.8	22.5	-1.7168	4.8751	G	G	2.06 E-6	2.34 E+6	7 E+6	-1.5769	23.1551	G

TIGR		PP PPC / Pg Spectral Count						PP PPC/ Pg Protein Intensity						33277
PGN	*LANL*	*q*-value	PP (Norm)	PPC (Norm)	log_2Ratio	log_2Sum	Regulation		*q*-value	PP (Norm)	PPC (Norm)	log_2Ratio	log_2Sum	Consensus
		1.76 E-1	50.5	35.5	0.5080	6.4260	Y	Y	8.83 E-2	2.63 E+7	2.34 E+7	0.1677	25.5644	Y
PGN0059														
		2.15 E-1	4.3	1	2.0970	2.4001	Y	R	4.17 E-12	2.02 E+6	1 E+3	10.9783	20.9448	R
PGN0062														
		1.13 E-1	7.7	2	1.9450	3.2781	Y	R	0	3.78 E+6	1.09 E+5	5.1176	21.8905	R
PGN0063														
		9.55 E-3	10.3	1	3.3601	3.4941	Y	R	0	3.98 E+6	1 E+3	11.9577	21.9238	R
PGN0064														
		6.61 E-1	13.7	15	-0.1318	4.8425	Y	Y	1.97 E-1	3.61 E+6	3.57 E+6	0.0161	22.7774	Y
PGN0065														
		1.36 E-1	19.7	10	0.9767	4.8914	Y	R	4.42 E-4	6.64 E+6	2.85 E+6	1.2205	23.1769	R
PGN0071														
PG1486		3.82 E-1	1.7	4	-1.2249	2.5138	Y	G	7.61 E-16	1.85 E+3	2.41 E+5	-7.0242	17.8890	G
PGN0073	*PG1301*	*conjugative transposon protein TraA*												
		3.21 E-4	43.6	14	1.6402	5.8490	R	R	6.52 E-6	2.65 E+7	1.09 E+7	1.2792	25.1546	R
PGN0079														
PG1447		2.78 E-1	40.2	29.5	0.4470	6.1234	Y	Y	1.26 E-1	2.01 E+7	2.26 E+7	-0.1693	25.3475	Y
PGN0082	*PG1266*	*transcriptional regulator AraC family*												
		4 E-11	122.4	36.5	1.7451	7.3116	R	R	0	6.72 E+7	1.15 E+7	2.5484	26.2301	R
PGN0083														
		1.52 E-4	59.9	22.5	1.4125	6.3645	R	R	0	2.9 E+7	5.61 E+6	2.3714	25.0463	R
PGN0084														
PG0816		6.69 E-1	1.7	1.5	0.1901	1.6832	Y	G	0	1.85 E+3	2.52 E+5	-7.0866	17.9509	Y
PGN0085	*PG0734*	*hypothetical protein PG_0816*												
		4.55 E-1	5.1	3	0.7751	3.0240	Y	R	6.78 E-4	7.91 E+6	2.19 E+5	5.1765	22.9549	R
PGN0086														
		2.1 E-2	28.2	52	-0.8809	6.3262	Y	G	1.42 E-4	8.26 E+6	1.35 E+7	-0.7116	24.3769	G
PGN0087														
		9.44 E-2	6.0	1	2.5825	2.8052	Y	R	4.46 E-8	3.15 E+6	1 E+3	11.6198	21.5861	R
PGN0091														
PG1113		4.64 E-4	15.4	1	3.9450	4.0358	R	R	1.04 E-6	3.04 E+7	1 E+3	14.8900	24.8558	R
PGN0094	*PG0994*	*integrase*												
PG2189		2.74 E-1	8.6	4	1.0970	3.6504	Y	R	3.75 E-6	3.31 E+6	1.25 E+6	1.4028	22.1203	R
PGN0096	*PG1916*	*lysC aspartate kinase*												

TIGR		PP PPC / Pg Spectral Count							PP PPC/ Pg Protein Intensity					33277
PGN	***LANL***	*q*-value	PP (Norm)	PPC (Norm)	$\log_2$Ratio	$\log_2$Sum	Regulation		*q*-value	PP (Norm)	PPC (Norm)	$\log_2$Ratio	$\log_2$Sum	Consensus
PG2190		6.02 E-1	5.1	4	0.3601	3.1912	Y	Y	1.87 E-1	1.4 E+6	1.45 E+6	-0.0460	21.4423	Y
PGN0097	*PG1917*	*ftsE cell-division ATP-binding protein*												
		2.72 E-1	71.0	87.5	-0.3011	7.3085	Y	Y	1.54 E-2	2.69 E+7	3.57 E+7	-0.4075	25.8999	Y
PGN0100														
		6 E-1	1.7	2.5	-0.5468	2.0743	Y	G	1.02 E-6	1.85 E+3	1.64 E+5	-6.4683	17.3382	G
PGN0104														
PG2182		2.67 E-1	52.2	39.5	0.4021	6.5188	Y	Y	1.99 E-2	4.11 E+7	3.1 E+7	0.4063	26.1036	Y
PGN0114	*PG1911*	*nqrA NADH:ubiquinone oxidoreductase Na translocating A subunit*												
PG2181		4.75 E-1	17.1	21.5	-0.3292	5.2710	Y	Y	1.91 E-2	3.1 E+6	8.65 E+6	-1.4800	23.4858	Y
PGN0115	*PG1910*	*nqrB NADH:ubiquinone oxidoreductase Na translocating B subunit*												
PG2180		4.97 E-1	15.4	12	0.3601	4.7762	Y	Y	1.47 E-3	1.26 E+7	8.66 E+6	0.5397	24.3410	Y
PGN0116	*PG1909*	*nqrC NADH:ubiquinone oxidoreductase Na translocating C subunit*												
PG2178		6.69 E-1	1.7	1.5	0.1901	1.6832	Y	G	0	1.85 E+3	1.3 E+5	-6.1398	17.0138	Y
PGN0118	*PG1907*	*nqrE NADH:ubiquinone oxidoreductase Na translocating E subunit*												
PG2177		4.98 E-2	79.6	53	0.5863	7.0507	Y	Y	4.49 E-2	3.99 E+7	3.27 E+7	0.2865	26.1130	Y
PGN0119	*PG1906*	*nqrF NADH:ubiquinone oxidoreductase Na translocating F subunit*												
PG2175		1.94 E-2	47.9	25	0.9386	6.1882	Y	R	2.79 E-8	5.54 E+7	1.15 E+7	2.2698	25.9949	R
PGN0120	*PG1903*	*conserved hypothetical protein*												
PG2174		3.17 E-2	211.3	163	0.3747	8.5482	Y	Y	1.91 E-1	9.48 E+7	9.38 E+7	0.0146	27.4909	Y
PGN0121	*PG1902*	*hypothetical protein PG_2174*												
PG2173		3.28 E-1	71.9	86	-0.2589	7.3026	Y	Y	9.87 E-3	5.13 E+7	7.82 E+7	-0.6096	26.9478	Y
PGN0122	*PG1901*	*omp28 outer membrane lipoprotein Omp28*												
PG2172		3.05 E-2	48.8	27	0.8531	6.2436	Y	Y	1.22 E-1	4.27 E+7	3.46 E+7	0.3026	26.2049	Y
PGN0123	*PG1899*	*hypothetical protein PG_2172*												
PG2171		6.64 E-1	80.4	77.5	0.0536	7.3032	Y	R	2.57 E-6	1.51 E+7	8.31 E+6	0.8632	24.4816	R
PGN0125	*PG1898*	*D-isomer specific 2-hydroxyacid dehydrogenase family protein*												
PG2170		4.75 E-1	3.4	5.5	-0.6843	3.1575	Y	Y	6.91 E-3	8.21 E+5	3.17 E+6	-1.9506	21.9288	Y
PGN0126	*PG1897*	*sugar transporter*												
PG2167		1.13 E-4	248.1	162	0.6152	8.6800	R	Y	7.12 E-2	3.82 E+8	3.38 E+8	0.1762	29.4243	R
PGN0128	*PG1893*	*immunoreactive 53 kDa antigen PG123*												
PG2168		0	6.0	90	-3.9094	6.5848	G	G	2.01 E-5	1.15 E+6	5.4 E+7	-5.5588	25.7179	G
PGN0129	*PG1894*	*hypothetical protein PG_2168*												
PG2080		4.4 E-1	5.1	8	-0.6399	3.7152	Y	Y	1.99 E-1	1.44 E+6	1.45 E+6	-0.0097	21.4671	Y
PGN0133	*PG1818*	*bioA adenosylmethionine--8-amino-7-oxononanoate aminotransferase*												

TIGR	PGN	LANL	Description	PP PPC / Pg Spectral Count q-value	PP (Norm)	PPC (Norm)	log_2Ratio	log_2Sum	Regulation (Spectral Count)	Regulation (Protein Intensity)	PP PPC/ Pg Protein Intensity q-value	PP (Norm)	PPC (Norm)	log_2Ratio	log_2Sum	33277 Consensus
PG2081	PGN0134	PG1819	*bioB biotin synthetase*	4.4 E-1	1.7	3.5	-1.0323	2.3816	Y	Y	2.07 E-2	1.85 E+3	6.7 E+5	-8.5007	19.3584	Y
PG2082	PGN0135	PG1820	*POT family protein*	1.72 E-1	30.8	45	-0.5468	6.2442	Y	Y	2.61 E-3	1.85 E+7	4.02 E+7	-1.1149	25.8075	Y
PG2083	PGN0136	PG1821	*hypothetical protein PG_2083*	5.82 E-3	35.1	13.5	1.3778	5.6023	R	Y	7.59 E-3	1.28 E+7	7.84 E+6	0.7012	24.2956	R
PG2085	PGN0137	PG1822	*trpS tryptophanyl-tRNA synthetase*	2.53 E-1	33.4	45.5	-0.4473	6.3014	Y	Y	1.17 E-3	1.42 E+7	2.37 E+7	-0.7429	25.1762	Y
PG2086	PGN0138	PG1823	*hypothetical protein PG_2086*	5.38 E-1	71.0	64.5	0.1389	7.0824	Y	Y	1.23 E-2	2.69 E+7	5.82 E+7	-1.1160	26.3425	Y
PG2088	PGN0140	PG1826	*msrA peptide methionine sulfoxide reductase*	6.54 E-1	8.6	7.5	0.1901	4.0051	Y	Y	5.06 E-2	5.46 E+6	3.57 E+6	0.6132	23.1074	Y
PG2089	PGN0141	PG1827	*hypothetical protein PG_2089*	6 E-1	6.0	7.5	-0.3244	3.7538	Y	Y	3.48 E-3	1.04 E+6	2.69 E+6	-1.3625	21.8309	Y
PG2090	PGN0142	PG1828	*cation efflux family protein*	3.82 E-1	1.7	4	-1.2249	2.5138	Y	Y	6.8 E-3	1.85 E+3	1.19 E+6	-9.3244	20.1803	Y
PG2091	PGN0143	PG1829	*folB dihydroneopterin aldolase*	5.77 E-2	3.4	12	-1.8099	3.9470	Y	G	7.86 E-6	2.93 E+5	2.67 E+6	-3.1868	21.4974	G
PG2092	PGN0144	PG1830	*hypothetical protein PG_2092*	2.72 E-2	12.0	28	-1.2249	5.3212	Y	Y	1.97 E-2	3.81 E+6	5.73 E+6	-0.5893	23.1844	Y
PG2094	PGN0145	PG1831	*conserved domain protein*	4.8 E-1	4.3	2.5	0.7751	2.7609	Y	R	0	8.39 E+5	2.43 E+5	1.7854	20.0460	R
PG2095	PGN0147	PG1832	*lipoprotein putative*	6 E-1	1.7	2.5	-0.5468	2.0743	Y	G	8.24 E-4	1.85 E+3	5.76 E+5	-8.2822	19.1406	G
PG2096	PGN0148	PG1833	*conserved domain protein*	3.05 E-1	3.4	1	1.7751	2.1449	Y	R	1.5 E-15	3.77 E+5	1 E+3	8.5586	18.5282	R
PG2097	PGN0149	PG1834	*prsA ribose-phosphate pyrophosphokinase*	1.84 E-1	113.8	91	0.3226	7.6781	Y	Y	9.6 E-2	7.05 E+7	6.24 E+7	0.1756	26.9859	Y
PG2099	PGN0150	PG1835	*ATP-dependent RNA helicase DEAD/DEAH box family*	5.17 E-1	1.7	3	-0.8099	2.2361	Y	Y	1.44 E-1	1.03 E+6	1.21 E+6	-0.2274	21.0942	Y
PG2101	PGN0151	PG1837	*hypothetical protein PG_2101*	9.37 E-6	36.8	6.5	2.5009	5.4361	R	R	0	3.48 E+7	6.63 E+6	2.3920	25.3042	R
PG2102	PGN0152	PG1838	*immunoreactive 61 kDa antigen PG91*	6.61 E-8	690.5	923.5	-0.4194	10.6564	G	Y	1.46 E-3	4.96 E+8	6.88 E+8	-0.4732	30.1411	G

TIGR		PP PPC / Pg Spectral Count							PP PPC/ Pg Protein Intensity					33277
PGN	*LANL*	*q*-value	PP (Norm)	PPC (Norm)	log_2Ratio	log_2Sum	Regulation		*q*-value	PP (Norm)	PPC (Norm)	log_2Ratio	log_2Sum	Consensus
PG2105		1.51 E-1	12.0	5	1.2605	4.0857	Y	R	2.03 E-9	8.61 E+6	1.9 E+6	2.1782	23.3250	R
PGN0154	*PG1839*	*lipoprotein putative*												
PG2106		4.62 E-1	292.6	275	0.0897	9.1488	Y	R	8 E-5	9.57 E+7	5.91 E+7	0.6956	27.2064	R
PGN0156	*PG1840*	*hypothetical protein PG_2106*												
PG2107		6.05 E-2	79.6	54	0.5594	7.0615	Y	Y	2.19 E-2	2.51 E+7	2 E+7	0.3270	25.4290	Y
PGN0157	*PG1841*	*thiH thiH protein*												
PG2108		7.02 E-1	43.6	43.5	0.0046	6.4452	Y	Y	1.08 E-2	2.64 E+7	4.66 E+7	-0.8185	26.1216	Y
PGN0158	*PG1843*	*thiG thiG protein*												
PG2109		6.69 E-1	17.1	16	0.0970	5.0493	Y	Y	5.96 E-2	7.05 E+6	5.41 E+6	0.3839	23.5708	Y
PGN0159	*PG1844*	*hydroxymethylpyrimidine kinase/thiamin-phosphate pyrophosphorylase*												
PG2110		1.61 E-1	83.0	106	-0.3529	7.5622	Y	G	2.88 E-9	3.26 E+7	7.66 E+7	-1.2344	26.7027	G
PGN0160	*PG1845*	*thiC thiamine biosynthesis protein ThiC*												
PG2112		3.5 E-1	4.3	8	-0.9030	3.6180	Y	G	2.81 E-4	3.06 E+5	1.46 E+6	-2.2548	20.7512	G
PGN0162	*PG1848*	*hypothetical protein PG_2112*												
PG2117		4.89 E-2	36.8	19.5	0.9160	5.8149	Y	R	1.82 E-4	3.5 E+7	1.91 E+7	0.8746	25.6870	R
PGN0167	*PG1850*	*rpsP ribosomal protein S16*												
		7.2 E-3	36.8	15	1.2945	5.6947	R	R	2.51 E-11	2.27 E+7	3.61 E+6	2.6548	24.6492	R
PGN0168														
PG2120		3.05 E-1	2.6	6	-1.2249	3.0988	Y	Y	1.28 E-2	1.71 E+5	1.56 E+6	-3.1873	20.7192	Y
PGN0169	*PG1853*	*metallo-beta-lactamase superfamily protein*												
PG2121		1.57 E-2	18.0	5	1.8455	4.5216	Y	R	3.67 E-15	8.3 E+6	2.8 E+6	1.5689	23.4037	R
PGN0170	*PG1854*	*ansA L-asparaginase*												
PG2124		9.97 E-8	735.0	971.5	-0.4025	10.7368	G	Y	1.01 E-2	4.1 E+8	5.19 E+8	-0.3387	29.7905	G
PGN0173	*PG1857*	*gapA glyceraldehyde 3-phosphate dehydrogenase type I*												
PG2126		2.52 E-1	5.1	10.5	-1.0323	3.9666	Y	Y	1.9 E-1	1.85 E+6	1.81 E+6	0.0254	21.8042	Y
PGN0175	*PG1859*	*conserved hypothetical protein TIGR00044*												
PG2127		1.84 E-1	28.2	41.5	-0.5555	6.1238	Y	Y	1.07 E-1	2.13 E+7	2.67 E+7	-0.3246	25.5169	Y
PGN0176	*PG1860*	*hypothetical protein PG_2127*												
PG2130		0	2.6	223.5	-6.4441	7.8206	G	G	3.16 E-7	3.94 E+5	1.11 E+8	-8.1375	26.7318	G
PGN0178	*PG1863*	*hypothetical protein PG_2130*												
PG2131		3.11 E-1	405.6	373	0.1208	9.6047	Y	Y	5.87 E-2	4.57 E+8	4.04 E+8	0.1790	29.6809	Y
PGN0179	*PG1864*	*60 kDa protein*												
PG2132		0	426.1	1623	-1.9293	11.0008	G	G	0	1.59 E+8	6.94 E+8	-2.1299	29.6669	G
PGN0180	*PG1865*	*fimA fimbrilin*												

TIGR		PP PPC / Pg Spectral Count						PP PPC/ Pg Protein Intensity						33277
PGN	***LANL***	*q*-value	PP (Norm)	PPC (Norm)	log_2Ratio	log_2Sum	Regulation		*q*-value	PP (Norm)	PPC (Norm)	log_2Ratio	log_2Sum	Consensus
P13793I EMA_PORGI		0	426.1	1623	-1.9293	11.0008	G	G	0	1.59 E+8	6.94 E+8	-2.1299	29.6669	G
PGN0180		*major fimbrillin A, Porphyromonas gingivalis 33277*												
PG2134		3.03 E-3	9.4	30	-1.6724	5.3006	G	G	4.03 E-10	2.83 E+6	1.72 E+7	-2.6066	24.2566	G
PGN0183	*PG1867*	*lipoprotein putative*												
PG2135		4.08 E-10	7.7	58.5	-2.9253	6.0488	G	G	2.96 E-11	2.94 E+6	2.42 E+7	-3.0426	24.6930	G
PGN0184	*PG1868*	*lipoprotein putative*												
PG2136		1.74 E-6	19.7	66.5	-1.7566	6.4293	G	G	3.72 E-14	8.97 E+6	4.11 E+7	-2.1972	25.5778	G
PGN0185	*PG1869*	*hypothetical protein PG_2136*												
PG2140		9.44 E-2	6.0	1	2.5825	2.8052	Y	R	0	1.38 E+6	1 E+3	10.4275	20.3943	R
PGN0188		*rpmF ribosomal protein L32*												
PG2141		1.97 E-1	73.6	56	0.3940	7.0178	Y	Y	3.91 E-2	4.4 E+7	3.4 E+7	0.3690	26.2168	Y
PGN0189	*PG1872*	*fabH 3-oxoacyl-(acyl-carrier-protein) synthase III*												
PG2142		3.85 E-1	5.1	2.5	1.0381	2.9324	Y	Y	2.94 E-2	3.48 E+6	1.76 E+6	0.9840	22.3192	Y
PGN0190	*PG1873*	*era GTP-binding protein Era*												
PG2143		3.08 E-4	26.5	5	2.4074	4.9784	R	Y	6.55 E-3	4.83 E+6	2.41 E+6	1.0059	22.7871	R
PGN0191	*PG1874*	*GTP-binding protein Era/ThdF family*												
PG2144		6.9 E-1	32.5	32	0.0230	6.0116	Y	Y	1.61 E-1	1.6 E+7	1.52 E+7	0.0706	24.8927	Y
PGN0192	*PG1875*	*hypothetical protein PG_2144*												
PG2145		6.92 E-2	4.3	13	-1.6034	4.1109	Y	G	9.37 E-4	9.14 E+5	1.93 E+6	-1.0774	21.4384	G
PGN0193	*PG1876*	*polysaccharide deacetylase*												
PG2147		4.75 E-1	11.1	8	0.4755	4.2573	Y	Y	3.35 E-2	2.31 E+6	1.65 E+6	0.4832	21.9164	Y
PGN0195	*PG1878*	*xpt xanthine phosphoribosyltransferase*												
PG2150		5.52 E-3	16.3	3	2.4381	4.2673	R	R	0	5.39 E+6	1.26 E+6	2.1004	22.6634	R
PGN0198	*PG1881*	*LysM domain protein*												
PG2155		2.96 E-2	23.1	9	1.3601	5.0046	Y	R	1.14 E-6	3.14 E+7	1.06 E+7	1.5628	25.3260	R
PGN0200	*PG1883*	*lipoprotein putative*												
PG2156		6.69 E-1	1.7	1.5	0.1901	1.6832	Y	G	0	1.85 E+3	7.56 E+4	-5.3524	16.2410	Y
PGN0201	*PG1884*	*conserved hypothetical protein TIGR00046*												
PG2157		6.87 E-1	29.9	30.5	-0.0264	5.9176	Y	Y	1.53 E-2	1.43 E+7	2.01 E+7	-0.4959	25.0344	Y
PGN0202	*PG1885*	*glutamine cyclotransferase-related protein*												
PG2158		2.15 E-1	4.3	1	2.0970	2.4001	Y	R	0	1.75 E+6	1 E+3	10.7730	20.7396	R
PGN0203	*PG1886*	*conserved hypothetical protein*												
PG2159		6.94 E-1	7.7	7.5	0.0381	3.9261	Y	Y	9.92 E-2	1.69 E+6	1.38 E+6	0.2886	21.5495	Y
PGN0204	*PG1887*	*protoporphyrinogen oxidase*												

TIGR		PP PPC / Pg Spectral Count						PP PPC/ Pg Protein Intensity						33277
PGN	LANL	q-value	PP (Norm)	PPC (Norm)	log_2Ratio	log_2Sum	Regulation		q-value	PP (Norm)	PPC (Norm)	log_2Ratio	log_2Sum	Consensus
PG2162		6.54 E-1	2.6	2	0.3601	2.1912	Y	R	1.52 E-5	1.26 E+6	3.44 E+5	1.8728	20.6151	R
PGN0206	PG1889	lpxB lipid A disaccharide synthase												
PG2163		3.76 E-1	15.4	21.5	-0.4812	5.2056	Y	G	8.02 E-8	4.76 E+6	1.14 E+7	-1.2631	23.9486	G
PGN0207	PG1890	surE stationary-phase survival protein SurE												
PG2164		1.02 E-1	14.5	27	-0.8923	5.3766	Y	G	7.53 E-4	8.37 E+6	1.74 E+7	-1.0569	24.6204	G
PGN0208	PG1891	peptidyl-prolyl cis-trans isomerase FKBP-type												
PG2165		5.3 E-8	47.9	7	2.7751	5.7792	R	R	0	3.13 E+7	4 E+6	2.9676	25.0739	R
PGN0209	PG1892	glyS glycyl-tRNA synthetase												
PG2078		2.74 E-1	1.7	5	-1.5468	2.7466	Y	Y	2.08 E-3	2.83 E+5	7.07 E+5	-1.3197	19.9167	Y
PGN0211	PG1816	conserved hypothetical protein												
PG0104		1.5 E-1	1.7	6.5	-1.9253	3.0376	Y	Y	4.34 E-2	7.39 E+5	1.87 E+6	-1.3425	21.3172	Y
PGN0221	PG0091	topB-1 DNA topoisomerase III												
PG0108		5.74 E-1	18.8	16	0.2345	5.1220	Y	Y	1.69 E-2	6.76 E+6	4.66 E+6	0.5372	23.4453	Y
PGN0224	PG0094	epsD UDP-N-acetyl-D-mannosaminuronic acid dehydrogenase												
PG0111		6.41 E-1	6.0	5	0.2605	3.4581	Y	R	1.65 E-8	1.34 E+6	5.62 E+5	1.2493	20.8552	R
PGN0228	PG0097	capsular polysacharride biosynthesis gene putative												
		6.85 E-1	29.1	28.5	0.0297	5.8478	Y	R	3.31 E-10	1.7 E+7	4.65 E+6	1.8679	24.3666	R
PGN0229														
PG0118		6.69 E-1	12.0	11	0.1230	4.5223	Y	G	5.59 E-4	1.53 E+6	3.85 E+6	-1.3282	22.3603	Y
PGN0232	PG0103	glycosyl transferase group 2 family protein												
PG0119		2.55 E-1	7.7	14	-0.8623	4.4397	Y	Y	3.45 E-3	2.81 E+6	5.6 E+6	-0.9958	23.0022	Y
PGN0233	PG0104	glycosyl transferase WecB/TagA/CpsF family												
PG0120		3.31 E-1	20.5	14	0.5527	5.1100	Y	R	5.19 E-8	9.81 E+6	3.73 E+6	1.3954	23.6910	R
PGN0234	PG0105	epsC UDP-N-acetylglucosamine 2-epimerase												
PG0121		0	371.4	69	2.4281	8.7825	R	R	4.01 E-7	3.09 E+8	1.43 E+8	1.1141	28.7494	R
PGN0235	PG0106	hup-1 DNA-binding protein HU												
PG0123		3.21 E-1	1.7	4.5	-1.3948	2.6349	Y	Y	1.72 E-3	1.85 E+3	1.06 E+6	-9.1672	20.0234	Y
PGN0236	PG0107	hypothetical protein PG_0123												
PG0125		4.15 E-1	2.6	5	-0.9619	2.9197	Y	Y	3.52 E-3	9.2 E+4	9.17 E+5	-3.3170	19.9443	Y
PGN0238	PG0109	hypothetical protein PG_0125												
PG0126		6.55 E-1	5.1	6	-0.2249	3.4769	Y	Y	1.59 E-3	1.91 E+6	3.46 E+6	-0.8544	22.3560	Y
PGN0239	PG0110	type I phosphodiesterase/nucleotide pyrophosphatase family protein												
PG0127		6.97 E-1	2.6	2.5	0.0381	2.3411	Y	R	0	2.16 E+6	1.7 E+5	3.6719	21.1547	R
PGN0240	PG0112	hemH ferrochelatase												

TIGR		PP PPC / Pg Spectral Count							PP PPC/ Pg Protein Intensity					33277
PGN	*LANL*	*q*-value	PP (Norm)	PPC (Norm)	log_2Ratio	log_2Sum	Regulation		*q*-value	PP (Norm)	PPC (Norm)	log_2Ratio	log_2Sum	Consensus
PG0128		6.97 E-1	2.6	2.5	0.0381	2.3411	Y	R	0	4.82 E+5	1.13 E+5	2.0969	19.1831	R
PGN0241	*PG0113*	*conserved domain protein*												
PG0129		6.69 E-1	9.4	10	-0.0874	4.2789	Y	Y	1.77 E-1	2.35 E+6	2.2 E+6	0.0972	22.1155	Y
PGN0242	*PG0114*	*mannosyltransferase*												
PG0130		6.37 E-1	157.4	152	0.0507	8.2735	Y	Y	1.88 E-1	1.43 E+8	1.45 E+8	-0.0238	28.0990	Y
PGN0243	*PG0115*	*gpm phosphoglycerate mutase*												
PG0133		6.69 E-1	1.7	1.5	0.1901	1.6832	Y	G	0	1.85 E+3	7.82 E+4	-5.4011	16.2886	Y
PGN0246	*PG0118*	*hypothetical protein PG_0133*												
PG0134		5.17 E-1	3.4	2	0.7751	2.4390	Y	Y	3.88 E-2	9.02 E+5	5.33 E+5	0.7591	20.4530	Y
PGN0247	*PG0119*	*mgtE magnesium transporter*												
PG0136		4.02 E-1	4.3	2	1.0970	2.6504	Y	R	0	1.36 E+6	5.22 E+5	1.3862	20.8468	R
PGN0249	*PG0122*	*hypothetical protein PG_0136*												
PG0137		2.93 E-3	124.1	183	-0.5607	8.2624	G	G	3.51 E-5	5.84 E+7	1.11 E+8	-0.9201	27.3323	G
PGN0250	*PG0123*	*pepD-1 aminoacyl-histidine dipeptidase*												
PG0138		9.03 E-2	82.1	110	-0.4213	7.5860	Y	Y	2.47 E-3	1.12 E+8	1.86 E+8	-0.7243	28.1501	Y
PGN0251	*PG0124*	*fabD malonyl CoA-acyl carrier protein transacylase*												
PG0139		6.78 E-1	6.8	6.5	0.0747	3.7383	Y	Y	4.5 E-2	2.16 E+6	1.12 E+6	0.9507	21.6419	Y
PGN0252	*PG0125*	*membrane-bound lytic murein transglycosylase D putative*												
PG0140		6.69 E-1	1.7	2	-0.2249	1.8919	Y	G	0	1.85 E+3	8.51 E+5	-8.8458	19.7027	G
PGN0253	*PG0127*	*hypothetical protein PG_0140*												
PG0141		6.83 E-1	20.5	20	0.0381	5.3411	Y	Y	3.72 E-2	9.14 E+6	1.19 E+7	-0.3821	24.3281	Y
PGN0254	*PG0128*	*spo0J spoOJ protein*												
PG0142		1.86 E-1	5.1	11.5	-1.1635	4.0561	Y	Y	1.32 E-1	6.95 E+6	5.54 E+6	0.3278	23.5737	Y
PGN0255	*PG0129*	*soj SpoOJ regulator protein*												
PG0143		2.08 E-2	11.1	27.5	-1.3058	5.2714	Y	Y	9.12 E-3	3.17 E+6	5.41 E+6	-0.7727	23.0327	Y
PGN0256	*PG0130*	*hydrolase carbon-nitrogen family*												
PG0144		6.92 E-2	14.5	28.5	-0.9703	5.4278	Y	Y	3.17 E-3	6.22 E+6	1.29 E+7	-1.0533	24.1889	Y
PGN0257	*PG0131*	*conserved hypothetical protein*												
		6.61 E-1	11.1	10	0.1536	4.4008	Y	Y	1.05 E-1	7.21 E+6	5.72 E+6	0.3340	23.6245	Y
PGN0258														
PG0146		6.69 E-1	1.7	1.5	0.1901	1.6832	Y	G	0	1.85 E+3	4.41 E+5	-7.8960	18.7558	Y
PGN0259	*PG0134*	*hypothetical protein PG_0146*												
PG0148		1.45 E-1	5.1	1	2.3601	2.6168	Y	R	0	1.23 E+6	1 E+3	10.2697	20.2367	R
PGN0261	*PG0136*	*sigma-54-dependent transcriptional regulator*												

TIGR / PGN	LANL	PP PPC / Pg Spectral Count q-value	PP (Norm)	PPC (Norm)	log_2Ratio	log_2Sum	Regulation (Spectral Count)	Regulation (Protein Intensity)	PP PPC/ Pg Protein Intensity q-value	PP (Norm)	PPC (Norm)	log_2Ratio	log_2Sum	33277 Consensus
PG0149		6.69 E-1	2.6	3	-0.2249	2.4769	Y	Y	9.69 E-2	7.01 E+5	9.12 E+5	-0.3794	20.6209	Y
PGN0262	PG0137	conserved domain protein												
PG0150		6.69 E-1	1.7	2	-0.2249	1.8919	Y	G	0	1.85 E+3	1.14 E+7	-12.5834	23.4373	G
PGN0263	PG0138	conserved hypothetical protein TIGR01125												
PG0151		1.45 E-1	5.1	1	2.3601	2.6168	Y	R	6.34 E-7	1.45 E+6	1 E+3	10.5009	20.4677	R
PGN0264	PG0139	ftsY signal recognition particle-docking protein FtsY												
PG0152		5.47 E-3	9.4	28.5	-1.5984	5.2446	G	Y	5.67 E-2	2.78 E+6	3.55 E+6	-0.3542	22.5936	G
PGN0265	PG0140	nspC carboxynorspermidine decarboxylase												
PG0153		2.71 E-1	101.8	121.5	-0.2549	7.8030	Y	G	2.14 E-5	5.23 E+7	8.04 E+7	-0.6199	26.9837	G
PGN0266	PG0141	aspS aspartyl-tRNA synthetase												
PG0156		5.17 E-1	1.7	3	-0.8099	2.2361	Y	Y	7.27 E-3	1.85 E+3	6.07 E+5	-8.3570	19.2151	Y
PGN0268	PG0143	modification methylase HemK family												
PG0159		4.61 E-3	516.0	625	-0.2766	10.1560	G	Y	7.25 E-2	4.46 E+8	4.94 E+8	-0.1463	29.8075	G
PGN0271	PG0146	pepO endopeptidase PepO												
PG0160		5.8 E-1	78.7	73	0.1088	7.2453	Y	Y	1.27 E-1	5.52 E+7	6.05 E+7	-0.1319	26.7867	Y
PGN0272	PG0147	conserved domain protein												
PG0163		6.61 E-1	9.4	10.5	-0.1578	4.3156	Y	Y	7.32 E-2	3.75 E+6	2.71 E+6	0.4653	22.6228	Y
PGN0275	PG0150	pfk phosphofructokinase												
PG0166		6 E-1	1.7	2.5	-0.5468	2.0743	Y	G	0	1.85 E+3	4.07 E+5	-7.7796	18.6398	G
PGN0278	PG0153	pth peptidyl-tRNA hydrolase												
PG0167		6.58 E-12	127.5	37	1.7848	7.3619	R	R	0	1.4 E+8	3.03 E+7	2.2031	27.3407	R
PGN0279	PG0154	rplY ribosomal protein L25												
PG0170		7.02 E-1	131.8	131.5	0.0030	8.0404	Y	Y	8.97 E-2	7.56 E+7	6.81 E+7	0.1509	27.0986	Y
PGN0281	PG0156	metG methionyl-tRNA synthetase												
PG0171		1.77 E-1	13.7	23.5	-0.7795	5.2169	Y	Y	1.4 E-1	7.5 E+6	8.41 E+6	-0.1643	23.9237	Y
PGN0282	PG0157	5'-nucleotidase family protein												
PG0172		6.69 E-1	1.7	1.5	0.1901	1.6832	Y	G	0	1.85 E+3	1.78 E+5	-6.5842	17.4529	Y
PGN0283	PG0158	exonuclease												
		1.56 E-1	6.0	1.5	1.9975	2.9049	Y	R	0	2.02 E+6	3.35 E+5	2.5881	21.1659	R
PGN0285														
PG0178		0	2104.9	3146.5	-0.5800	12.3585	G	G	6.61 E-4	1.15 E+9	1.71 E+9	-0.5709	31.4148	G
PGN0287	PG0163													
PG0179		1.53 E-2	162.6	217	-0.4166	8.5682	Y	Y	1.07 E-2	1.06 E+8	1.65 E+8	-0.6375	28.0162	Y
PGN0288	PG0164	hypothetical protein PG_0179												

TIGR	PGN	LANL	Description	PP PPC / Pg Spectral Count q-value	PP (Norm)	PPC (Norm)	log_2Ratio	log_2Sum	Regulation (Spectral Count)	Regulation (Protein Intensity)	PP PPC/ Pg Protein Intensity q-value	PP (Norm)	PPC (Norm)	log_2Ratio	log_2Sum	33277 Consensus
PG0180	PGN0289	PG0165	lipoprotein putative	5.47 E-12	14.5	83	-2.5125	6.6080	G	G	5.28 E-12	7.96 E+6	3.12 E+7	-1.9707	25.2234	G
PG0181	PGN0290	PG0166	immunoreactive 32 kDa antigen PG49	3.03 E-2	76.2	110.5	-0.5371	7.5442	Y	Y	1.09 E-3	5.14 E+7	1.21 E+8	-1.2347	27.3603	Y
PG0182	PGN0291	PG0167	von Willebrand factor type A domain protein	2.44 E-8	109.5	216.5	-0.9831	8.3488	G	G	1.65 E-9	7.77 E+7	1.44 E+8	-0.8873	27.7226	G
	PGN0292			4.23 E-1	2.6	1	1.3601	1.8347	Y	R	0	2.27 E+6	1 E+3	11.1476	21.1141	R
PG0185	PGN0293	PG0170	ragA ragA protein	1.07 E-7	3902.6	3413.5	0.1932	12.8369	R	G	4.08 E-6	3.63 E+9	5.1 E+9	-0.4891	33.0235	Y
PG0186	PGN0294	PG0171	ragB lipoprotein RagB	3.06 E-2	1035.3	1153	-0.1553	11.0956	Y	G	3.25 E-4	1.74 E+9	2.35 E+9	-0.4334	31.9293	G
	PGN0295			4.75 E-1	72.7	81.5	-0.1642	7.2689	Y	Y	3.11 E-2	2.68 E+8	4.13 E+8	-0.6211	29.3435	Y
PG0188	PGN0296	PG0172	lipoprotein putative	6.9 E-1	26.5	27	-0.0256	5.7421	Y	Y	4.87 E-2	1.34 E+7	1.02 E+7	0.3890	24.4898	Y
PG0189	PGN0297	PG0173	hypothetical protein PG_0189	4.82 E-2	1.7	9	-2.3948	3.4211	Y	G	1.56 E-7	1.85 E+3	2.38 E+6	-10.3303	21.1851	G
PG0190	PGN0298	PG0174	uppS undecaprenyl diphosphate synthase	2.52 E-1	3.4	8	-1.2249	3.5138	Y	Y	1.63 E-2	9.27 E+5	2.78 E+6	-1.5864	21.8238	Y
PG0191	PGN0299	PG0175	outer membrane protein putative	3.05 E-1	47.1	59.5	-0.3384	6.7355	Y	Y	8.65 E-2	2.92 E+7	2.61 E+7	0.1625	25.7208	Y
PG0192	PGN0300	PG0176	ompH-1 cationic outer membrane protein OmpH	2.83 E-3	161.7	106.5	0.6026	8.0673	R	Y	1.42 E-2	9.62 E+7	5.88 E+7	0.7106	27.2082	R
PG0193	PGN0301	PG0177	ompH-2 cationic outer membrane protein OmpH	8.96 E-2	103.5	76.5	0.4366	7.4921	Y	Y	1.96 E-1	1.09 E+8	1.1 E+8	-0.0138	27.7060	Y
PG0195	PGN0302	PG0179	rubrerythrin	3.22 E-6	101.0	186	-0.8814	8.1647	G	Y	4.8 E-3	4.49 E+7	9.18 E+7	-1.0300	27.0264	G
PG0196	PGN0303	PG0180	peptidase M16 family	1.57 E-1	366.2	413.5	-0.1752	9.6068	Y	Y	1.04 E-1	2.98 E+8	3.21 E+8	-0.1058	29.2068	Y
PG0198	PGN0306	PG0182	conserved hypothetical protein	6.69 E-1	1.7	2	-0.2249	1.8919	Y	G	0	1.85 E+3	1.04 E+6	-9.1314	19.9877	G
PG0199	PGN0307	PG0183	TatD family protein	5.17 E-1	1.7	3	-0.8099	2.2361	Y	G	0	1.85 E+3	7.23 E+5	-8.6107	19.4681	G

TIGR / *PGN*	*LANL*	PP PPC / Pg Spectral Count *q*-value	PP (Norm)	PPC (Norm)	log_2Ratio	log_2Sum	Regulation		PP PPC/ Pg Protein Intensity *q*-value	PP (Norm)	PPC (Norm)	log_2Ratio	log_2Sum	33277 Consensus
PG0201		3.82 E-1	1.7	4	-1.2249	2.5138	Y	G	4.31 E-7	1.85 E+3	1.84 E+5	-6.6391	17.5073	G
PGN0309	*PG0184*	*rnpA ribonuclease P protein component*												
PG0202		4.23 E-1	6.8	4	0.7751	3.4390	Y	R	2.81 E-5	4.21 E+6	8.42 E+5	2.3210	22.2675	R
PGN0310	*PG0185*	*uroporphyrinogen-III synthase HemD putative*												
PG0205		1.4 E-1	14.5	6.5	1.1621	4.3955	Y	Y	2.31 E-2	5.01 E+6	3.35 E+6	0.5805	22.9964	Y
PGN0313	*PG0188*	*prfC peptide chain release factor 3*												
PG0209		6.69 E-1	1.7	2	-0.2249	1.8919	Y	G	6.68 E-8	1.85 E+3	2.05 E+5	-6.7916	17.6583	G
PGN0314	*PG0189*	*formate/nitrite transporter*												
PG0210		4.23 E-1	2.6	1	1.3601	1.8347	Y	R	0	1.74 E+5	1 E+3	7.4469	17.4210	R
PGN0315	*PG0190*	*precorrin-6x reductase/cobalamin biosynthetic protein CbiD*												
PG0211		6 E-1	5.1	6.5	-0.3404	3.5403	Y	Y	3.93 E-2	2.72 E+6	1.94 E+6	0.4867	22.1539	Y
PGN0316	*PG0191*	*cbiGF cobalamin biosynthesis protein CbiG/precorrin-4 C11-methyltransferase*												
PG0213		2.25 E-1	6.8	2.5	1.4532	3.2242	Y	Y	2.41 E-2	1.29 E+6	5.9 E+5	1.1326	20.8440	Y
PGN0318	*PG0193*	*precorrin-3 methylase/precorrin-8X methylmutase*												
PG0217		1.12 E-2	10.3	28	-1.4473	5.2581	Y	G	1.9 E-4	4.36 E+6	1.24 E+7	-1.5057	23.9962	G
PGN0322	*PG0197*	*hypothetical protein PG_0217*												
PG0218		4.89 E-1	5.1	7.5	-0.5468	3.6592	Y	Y	1.53 E-2	1.13 E+6	2.64 E+6	-1.2264	21.8435	Y
PGN0323	*PG0198*	*hypothetical protein PG_0218*												
		5.74 E-1	4.3	3	0.5121	2.8636	Y	Y	6.07 E-3	1.12 E+7	1.67 E+6	2.7523	23.6223	Y
PGN0326														
PG0226		6.69 E-1	142.0	144.5	-0.0248	8.1626	Y	Y	5.98 E-2	7.61 E+7	8.39 E+7	-0.1408	27.2531	Y
PGN0329	*PG0206*	*transglutaminase-related protein*												
PG0227		3.21 E-1	1.7	4.5	-1.3948	2.6349	Y	Y	1.99 E-3	1.85 E+3	6.91 E+5	-8.5448	19.4023	Y
PGN0330	*PG0207*	*radA DNA repair protein RadA*												
PG0228		6.15 E-1	14.5	16.5	-0.1818	4.9563	Y	Y	1.75 E-1	8.69 E+6	8.38 E+6	0.0528	24.0247	Y
PGN0331	*PG0208*	*DdaH family protein*												
PG0230		1.17 E-1	96.7	124.5	-0.3647	7.7891	Y	Y	6.61 E-3	9.04 E+7	1.45 E+8	-0.6782	27.8085	Y
PGN0333	*PG0209*	*transaldolase TalC putative*												
PG0231		6.69 E-1	3.4	4	-0.2249	2.8919	Y	R	1.15 E-7	4.35 E+5	2.5 E+5	0.7977	19.3876	Y
PGN0334	*PG0211*	*conserved hypothetical protein*												
PG0232		5.87 E-2	201.1	248.5	-0.3055	8.8124	Y	Y	3.28 E-2	1.95 E+8	2.38 E+8	-0.2843	28.6904	Y
PGN0335	*PG0212*	*zinc carboxypeptidase putative*												
		1.32 E-2	2.6	13.5	-2.3948	4.0060	Y	G	2.76 E-5	5.68 E+5	2.2 E+6	-1.9553	21.4030	G
PGN0336														

TIGR		PP PPC / Pg Spectral Count							PP PPC/ Pg Protein Intensity					33277
PGN	*LANL*	*q*-value	PP (Norm)	PPC (Norm)	log_2Ratio	log_2Sum	Regulation		*q*-value	PP (Norm)	PPC (Norm)	log_2Ratio	log_2Sum	Consensus
PG0236		1.12 E-1	7.7	17	-1.1424	4.6265	Y	G	4.24 E-4	6.32 E+6	1.12 E+7	-0.8310	24.0667	G
PGN0341	*PG0215*	*hypothetical protein PG_0236*												
PG0237		3.33 E-1	11.1	17	-0.6119	4.8137	Y	Y	8.91 E-3	2.55 E+6	1.82 E+6	0.4850	22.0614	Y
PGN0342	*PG0216*	*ung uracil-DNA glycosylase*												
PG0240		2.25 E-1	1.7	5.5	-1.6843	2.8503	Y	G	0	1.85 E+3	6.14 E+5	-8.3753	19.2333	G
PGN0344	*PG0218*	*hydrolase haloacid dehalogenase-like family*												
PG0241		3.49 E-2	90.7	60.5	0.5842	7.2403	Y	R	6.52 E-11	1.1 E+8	3.93 E+7	1.4841	27.1535	R
PGN0345	*PG0219*	*lipoprotein putative*												
PG0242		6.68 E-1	20.5	22	-0.0994	5.4106	Y	Y	6.45 E-2	4.97 E+6	3.84 E+6	0.3718	23.0708	Y
PGN0346	*PG0220*	*conserved hypothetical protein TIGR00096*												
PG0243		5.89 E-1	7.7	9.5	-0.3029	4.1044	Y	Y	8.23 E-2	3.61 E+6	4.7 E+6	-0.3820	22.9855	Y
PGN0347	*PG0221*	*hypothetical protein PG_0243*												
PG0245		0	94.1	9.5	3.3085	6.6952	R	R	0	5.73 E+7	3.68 E+6	3.9599	25.8619	R
PGN0348	*PG0222*	*universal stress protein family*												
PG0246		0	220.8	27.5	3.0050	7.9557	R	Y	2.85 E-2	7.62 E+7	5.4 E+7	0.4976	26.9555	R
PGN0349	*PG0223*	*hypothetical protein PG_0246*												
PG0249		3.12 E-4	186.5	117	0.6729	8.2457	R	R	8.92 E-4	1.38 E+8	1.04 E+8	0.4168	27.8516	R
PGN0351	*PG0226*	*oxaloacetate decarboxylase putative*												
PG0253		6.05 E-2	6.8	1	2.7751	2.9718	Y	R	0	9.37 E+5	1 E+3	9.8718	19.8391	R
PGN0353	*PG0228*	*conserved hypothetical protein*												
PG0254		3.2 E-6	120.6	54.5	1.1465	7.4524	R	R	2.97 E-12	5.67 E+7	2.71 E+7	1.0678	26.3198	R
PGN0354	*PG0229*	*N utilization substance protein A putative*												
PG0255		1.57 E-5	71.0	25.5	1.4777	6.5927	R	R	1.14 E-12	3.93 E+7	1.39 E+7	1.4944	25.6648	R
PGN0355	*PG0230*	*infB translation initiation factor IF-2*												
PG0257		6.61 E-1	9.4	10.5	-0.1578	4.3156	Y	Y	1.77 E-1	4.95 E+6	5.29 E+6	-0.0967	23.2882	Y
PGN0357	*PG0232*	*conserved hypothetical protein*												
PG0258		1.22 E-1	41.1	26	0.6596	6.0676	Y	R	6.21 E-6	2.1 E+7	9.51 E+6	1.1442	24.8642	R
PGN0358	*PG0233*	*ABC transporter ATP-binding protein*												
PG0259		7.06 E-1	12.0	12	-0.0025	4.5837	Y	Y	8.29 E-3	7.54 E+6	4.76 E+6	0.6634	23.5526	Y
PGN0359	*PG0235*	*conserved hypothetical protein*												
PG0263		0	101.0	12.5	3.0139	6.8261	R	R	2.11 E-13	4.53 E+7	1.19 E+7	1.9328	25.7689	R
PGN0360	*PG0237*	*tyrS tyrosyl-tRNA synthetase*												
PG0264		3.05 E-1	3.4	1	1.7751	2.1449	Y	R	0	6.82 E+5	1 E+3	9.4130	19.3809	R
PGN0361	*PG0238*	*glycosyl transferase group 2 family protein*												

TIGR		PP PPC / Pg Spectral Count						PP PPC/ Pg Protein Intensity						33277
PGN	*LANL*	*q*-value	PP (Norm)	PPC (Norm)	log_2Ratio	log_2Sum	Regulation		*q*-value	PP (Norm)	PPC (Norm)	log_2Ratio	log_2Sum	Consensus
PG0267		1.62 E-5	83.0	33	1.3306	6.8580	R	R	0	3.98 E+7	1.33 E+7	1.5797	25.6624	R
PGN0365	*PG0239*	*argS arginyl-tRNA synthetase*												
PG0268		1.62 E-1	8.6	3	1.5121	3.5306	Y	Y	1.19 E-1	8.17 E+5	1.16 E+6	-0.5118	20.9184	Y
PGN0366	*PG0240*	*trmU tRNA (5-methylaminomethyl-2-thiouridylate)-methyltransferase*												
PG0269		4.53 E-1	12.0	16	-0.4175	4.8063	Y	Y	6.45 E-2	5.26 E+6	7.11 E+6	-0.4344	23.5614	Y
PGN0367	*PG0241*	*xth exodeoxyribonuclease III*												
PG0270		1.9 E-1	12.0	5.5	1.1230	4.1276	Y	Y	1.6 E-1	3.18 E+6	2.92 E+6	0.1239	22.5416	Y
PGN0368	*PG0242*	*oxyR redox-sensitive transcriptional activator OxyR*												
PG0271		2.32 E-1	12.0	20	-0.7395	4.9991	Y	Y	9.94 E-2	5.13 E+6	6.58 E+6	-0.3595	23.4813	Y
PGN0369	*PG0243*	*ssb single-stranded binding protein*												
PG0272		5.89 E-1	1.7	1	0.7751	1.4390	Y	R	0	3.17 E+5	1 E+3	8.3077	18.2780	R
PGN0370	*PG0244*	*CBS domain protein*												
PG0275		2.74 E-1	29.1	20	0.5406	5.6174	Y	Y	1.85 E-1	2.08 E+7	2.21 E+7	-0.0864	25.3541	Y
PGN0373	*PG0246*	*thioredoxin family protein*												
PG0276		7.85 E-2	39.4	23	0.7751	5.9625	Y	Y	6.61 E-2	1.52 E+7	1.12 E+7	0.4413	24.6518	Y
PGN0374	*PG0248*	*conserved hypothetical protein*												
PG1745		8.8 E-3	27.4	9.5	1.5272	5.2048	R	Y	1.75 E-1	8.83 E+6	1.02 E+7	-0.2038	24.1794	Y
PGN0375	*PG1521*	*phosphoribulokinase family protein*												
PG1743		1.6 E-1	5.1	12	-1.2249	4.0988	Y	G	6.05 E-4	2.39 E+6	7.07 E+6	-1.5654	23.1723	G
PGN0376	*PG1520*	*kdsA 2-dehydro-3-deoxyphosphooctonate aldolase*												
PG1741		3.18 E-3	339.7	258	0.3969	9.2233	R	R	2.14 E-13	2.4 E+8	9.33 E+7	1.3662	28.3140	R
PGN0377	*PG1518*	*aspA aspartate ammonia-lyase*												
PG1739		4.75 E-1	3.4	5.5	-0.6843	3.1575	Y	Y	2.94 E-2	1.16 E+6	6.09 E+5	0.9303	20.7547	Y
PGN0378	*PG1517*	*conserved domain protein*												
PG1735		6.69 E-1	1.7	1.5	0.1901	1.6832	Y	G	0	1.85 E+3	1.72 E+5	-6.5356	17.4048	Y
PGN0382	*PG1513*	*MutT/nudix family protein*												
PG1731		6 E-1	4.3	5.5	-0.3624	3.2896	Y	Y	4.57 E-2	1.51 E+6	3.28 E+6	-1.1224	22.1908	Y
PGN0386	*PG1509*	*aroQ 3-dehydroquinate dehydratase type II*												
PG1729		4.52 E-7	134.3	59.5	1.1749	7.5987	R	R	4.63 E-9	1.55 E+8	6.93 E+7	1.1632	27.7427	R
PGN0388	*PG1507*	*thiol peroxidase*												
PG1727		6.97 E-1	2.6	2.5	0.0381	2.3411	Y	G	0	3.97 E+5	8.82 E+5	-1.1509	20.2870	Y
PGN0390	*PG1505*	*yitL yitL protein*												
PG1726		8.61 E-4	37.6	12	1.6496	5.6337	R	R	0	1.98 E+7	5.58 E+6	1.8301	24.5985	R
PGN0391	*PG1504*	*PDZ domain protein*												

TIGR		PP PPC / Pg Spectral Count							PP PPC/ Pg Protein Intensity					33277
PGN	*LANL*	*q*-value	PP (Norm)	PPC (Norm)	log_2Ratio	log_2Sum	Regulation		*q*-value	PP (Norm)	PPC (Norm)	log_2Ratio	log_2Sum	Consensus
PG1724		2.6 E-2	5.1	17	-1.7274	4.4682	Y	G	4.52 E-5	1.21 E+6	5.03 E+6	-2.0604	22.5733	G
PGN0393	*PG1502*	*gcp O-sialoglycoprotein endopeptidase*												
PG1723		2.67 E-7	3.4	35.5	-3.3747	5.2825	G	G	2.25 E-9	2.78 E+6	1.41 E+7	-2.3388	24.0072	G
PGN0394	*PG1501*	*rpsT ribosomal protein S20*												
PG1721		6.69 E-1	24.8	23.5	0.0785	5.5944	Y	Y	2.97 E-3	1.05 E+7	6.29 E+6	0.7359	23.9987	Y
PGN0396	*PG1499*	*vacB ribonuclease R*												
PG1720		5.43 E-1	3.4	5	-0.5468	3.0743	Y	Y	2.72 E-2	1.07 E+6	1.76 E+6	-0.7251	21.4334	Y
PGN0397	*PG1498*	*conserved domain protein*												
PG1719		3.05 E-1	5.1	2	1.3601	2.8347	Y	R	1.16 E-9	2.25 E+6	5.7 E+5	1.9770	21.4251	R
PGN0398	*PG1497*	*ABC transporter ATP-binding protein MsbA family*												
PG1715		3.67 E-5	13.7	48.5	-1.8248	5.9586	G	G	1.77 E-5	1.01 E+7	2.47 E+7	-1.2883	25.0545	G
PGN0400	*PG1496*	*hypothetical protein PG_1715*												
PG1714		6.23 E-2	6.8	17.5	-1.3542	4.6056	Y	Y	3.5 E-2	1.41 E+7	6.81 E+6	1.0468	24.3148	Y
PGN0403	*PG1495*	*pdxH pyridoxamine-phosphate oxidase*												
PG1712		6.69 E-1	1.7	2	-0.2249	1.8919	Y	R	0	6.44 E+5	3.36 E+5	0.9361	19.9022	Y
PGN0405	*PG1493*	*alpha-12-mannosidase family protein*												
PG1711		3.5 E-1	19.7	27	-0.4562	5.5447	Y	Y	1.6 E-1	6.38 E+6	6.7 E+6	-0.0705	23.6403	Y
PGN0406	*PG1492*	*alpha-12-mannosidase family protein*												
PG1705		3.82 E-1	8.6	5	0.7751	3.7609	Y	R	3.86 E-8	3.32 E+6	8.3 E+5	2.0006	21.9852	R
PGN0410	*PG1486*													
PG1704		1.18 E-1	78.7	57	0.4658	7.0845	Y	R	9.77 E-8	5.96 E+7	3.35 E+7	0.8296	26.4723	R
PGN0411	*PG1485*	*thiol:disulfide interchange protein dsbD putative*												
PG1703		3.09 E-1	9.4	5	0.9126	3.8492	Y	Y	6.59 E-2	3.58 E+6	6.52 E+6	-0.8647	23.2687	Y
PGN0412	*PG1484*	*MazG family protein*												
PG1702		5.15 E-9	118.1	41	1.5261	7.3136	R	R	0	5.42 E+7	1.04 E+7	2.3859	25.9446	R
PGN0413	*PG1483*	*gyrB DNA gyrase B subunit*												
PG1701		2.17 E-2	12.8	30	-1.2249	5.4207	Y	Y	2 E-2	5.36 E+6	7.18 E+6	-0.4210	23.5804	Y
PGN0414	*PG1482*	*glutamine amidotransferase class II/dipeptidase*												
PG1697		2.82 E-1	7.7	3.5	1.1377	3.4855	Y	R	1.03 E-6	2.92 E+6	7.84 E+5	1.8957	21.8197	R
PGN0415	*PG1481*	*type II restriction endonuclease putative*												
PG1694		2.93 E-1	6.8	3	1.1901	3.2994	Y	R	1.24 E-4	1.18 E+6	2.35 E+5	2.3241	20.4303	R
PGN0418	*PG1479*	*conserved hypothetical protein*												
PG1693		6.69 E-1	1.7	2	-0.2249	1.8919	Y	G	0	1.85 E+3	9 E+5	-8.9261	19.7828	G
PGN0419	*PG1478*	*HesA/MoeB/ThiF family protein*												

TIGR	PGN	LANL	Description	PP PPC / Pg Spectral Count q-value	PP (Norm)	PPC (Norm)	log_2Ratio	log_2Sum	Regulation (Spectral Count)	Regulation (Protein Intensity)	PP PPC/ Pg Protein Intensity q-value	PP (Norm)	PPC (Norm)	log_2Ratio	log_2Sum	33277 Consensus
PG1692	PGN0420	PG1477	ABC transporter ATP-binding protein	4.84 E-1	4.3	6.5	-0.6034	3.4301	Y	Y	2.01 E-1	1.27 E+6	1.27 E+6	-0.0059	21.2751	Y
PG1688	PGN0423	PG1474	greA transcription elongation factor GreA	4.74 E-5	24.0	2.5	3.2605	4.7256	R	R	0	2.24 E+7	1.44 E+6	3.9654	24.5076	R
PG1687	PGN0424	PG1473	HIT family protein	3.82 E-1	21.4	15.5	0.4648	5.2052	Y	Y	5.01 E-2	2 E+7	1.17 E+7	0.7759	24.9167	Y
PG1684	PGN0426	PG1471	hypothetical protein PG_1684	0	1.7	66.5	-5.2802	6.0919	G	G	1.14 E-7	1.85 E+3	1.1 E+7	-12.5345	23.3884	G
PG1683	PGN0427	PG1470	conserved hypothetical protein	1.13 E-1	23.1	12	0.9450	5.1335	Y	R	1.05 E-10	1.04 E+7	3.03 E+6	1.7769	23.6781	R
PG1682	PGN0428	PG1469	glycosyl transferase group 1 family protein	5.89 E-1	1.7	1	0.7751	1.4390	Y	R	0	5.82 E+5	1 E+3	9.1839	19.1521	R
PG1681	PGN0429	PG1468	glycogen debranching enzyme archaeal type putative	6.42 E-1	14.5	13	0.1621	4.7838	Y	Y	8.22 E-2	4.66 E+6	3.59 E+6	0.3737	22.9756	Y
	PGN0430			3.05 E-1	2.6	6	-1.2249	3.0988	Y	Y	9.52 E-2	8.98 E+5	1.1 E+6	-0.2988	20.9336	Y
PG1677	PGN0433	PG1465	pgk phosphoglycerate kinase	8.08 E-7	176.3	90	0.9697	8.0567	R	Y	4.49 E-2	1.01 E+8	8.41 E+7	0.2660	27.4651	R
PG1676	PGN0434	PG1464	pckA phosphoenolpyruvate carboxykinase (ATP)	1.57 E-12	965.2	1321.5	-0.4533	11.1590	G	G	2.99 E-4	6.21 E+8	8.35 E+8	-0.4280	30.4391	G
PG1667	PGN0444	PG1455	outer membrane efflux protein	6.57 E-3	13.7	35	-1.3542	5.6056	G	G	3.41 E-6	6.59 E+6	1.66 E+7	-1.3349	24.4689	G
PG1666	PGN0445	PG1454	efflux transporter MFP component RND family	5.74 E-1	4.3	3	0.5121	2.8636	Y	R	0	3.09 E+6	2.25 E+5	3.7845	21.6623	R
PG1665	PGN0446	PG1453	ABC transporter permease protein putative	6.69 E-1	1.7	2	-0.2249	1.8919	Y	G	1.64 E-6	1.85 E+3	2.4 E+5	-7.0216	17.8863	G
PG1663	PGN0448	PG1451	ABC transporter ATP-binding protein	4.23 E-1	2.6	1	1.3601	1.8347	Y	R	0	2.18 E+5	1 E+3	7.7658	17.7382	R
PG1662	PGN0449	PG1450	hypothetical protein PG_1662	6.69 E-1	3.4	3	0.1901	2.6832	Y	G	1.34 E-4	1.74 E+6	3.92 E+6	-1.1697	22.4342	Y
PG1657	PGN0456	PG1446	mutB methylmalonyl-CoA mutase large subunit	2.57 E-1	38.5	27.5	0.4856	6.0445	Y	R	2.69 E-5	2.37 E+7	1.54 E+7	0.6200	25.2192	R
PG1656	PGN0457	PG1445	mutA methylmalonyl-CoA mutase small subunit	3 E-1	40.2	30	0.4228	6.1337	Y	Y	2.63 E-2	1.81 E+7	1.21 E+7	0.5792	24.8475	Y

TIGR	PGN	LANL	Description	PP PPC / Pg Spectral Count q-value	PP (Norm)	PPC (Norm)	log_2Ratio	log_2Sum	Regulation (Spectral Count)	Regulation (Protein Intensity)	PP PPC/ Pg Protein Intensity q-value	PP (Norm)	PPC (Norm)	log_2Ratio	log_2Sum	33277 Consensus
	PGN0458			2.7 E-1	53.9	68.5	-0.3457	6.9355	Y	Y	3.41 E-3	3.74 E+7	6.38 E+7	-0.7719	26.5918	Y
PG1653	PGN0462	PG1443	*conserved hypothetical protein*	7.82 E-4	48.8	18.5	1.3985	6.0719	R	R	4.18 E-5	2.52 E+7	8.59 E+6	1.5532	25.0112	R
PG1652	PGN0463	PG1442	*hypothetical protein PG_1652*	2.4 E-7	15.4	63	-2.0323	6.2928	G	G	1.23 E-14	6.84 E+6	3.69 E+7	-2.4309	25.3829	G
PG1651	PGN0464	PG1441	*TPR domain protein*	5.08 E-2	39.4	21.5	0.8724	5.9274	Y	R	7.12 E-14	2.5 E+7	8.85 E+6	1.4964	25.0109	R
PG1648	PGN0465	PG1440	*RelA/SpoT family protein*	2.82 E-1	7.7	3.5	1.1377	3.4855	Y	R	6.8 E-4	5.56 E+6	2.35 E+5	4.5653	22.4655	R
PG1620	PGN0470	PG1417	*carboxyl-terminal protease-related protein*	2.32 E-1	4.3	9.5	-1.1509	3.7843	Y	Y	1.06 E-2	1.05 E+6	6.92 E+6	-2.7185	22.9263	Y
PG1621	PGN0471	PG1418	*hypothetical protein PG_1621*	6.69 E-1	1.7	1.5	0.1901	1.6832	Y	G	0	1.85 E+3	7.48 E+4	-5.3377	16.2267	Y
PG1622	PGN0472	PG1419	*DNA topoisomerase IV A subunit putative*	3.73 E-1	24.0	31.5	-0.3948	5.7933	Y	Y	3.56 E-2	5.93 E+6	7.74 E+6	-0.3841	23.7046	Y
PG1623	PGN0473	PG1420	*membrane bound regulatory protein putative*	5.43 E-1	6.8	9	-0.3948	3.9860	Y	Y	2.41 E-3	8.78 E+6	4.3 E+6	1.0275	23.6409	Y
PG1625	PGN0476	PG1423	*hypothetical protein PG_1625*	1.79 E-1	3.4	9	-1.3948	3.6349	Y	Y	3.84 E-2	1.29 E+6	1.03 E+6	0.3207	21.1470	Y
PG1626	PGN0477	PG1424	*hypothetical protein PG_1626*	0	2.6	69.5	-4.7589	6.1713	G	G	8.15 E-7	2.87 E+5	2.03 E+7	-6.1440	24.2959	G
PG1632	PGN0483	PG1426	*galM aldose 1-epimerase*	1.03 E-2	14.5	35	-1.2667	5.6307	Y	G	2.09 E-5	4.16 E+6	1.52 E+7	-1.8714	24.2085	G
PG1633	PGN0484	PG1427	*galK galactokinase*	3.71 E-2	41.9	22.5	0.8980	6.0096	Y	Y	1.46 E-1	2.43 E+7	2.99 E+7	-0.3012	25.6909	Y
PG1635	PGN0486	PG1429	*hypothetical protein PG_1635*	3.05 E-1	3.4	1	1.7751	2.1449	Y	R	0	6.04 E+6	1 E+3	12.5614	22.5274	R
PG1636	PGN0487	PG1430	*FtsK/SpoIIIE family protein*	3.49 E-1	15.4	10	0.6231	4.6669	Y	Y	1.53 E-3	7.12 E+6	4.14 E+6	0.7808	23.4247	Y
PG1638	PGN0488	PG1431	*thioredoxin family protein*	6.69 E-1	9.4	8.5	0.1471	4.1629	Y	Y	1.7 E-1	5.26 E+6	4.95 E+6	0.0856	23.2833	Y
PG1641	PGN0491	PG1433	*phosphotyrosine protein phosphatase*	2.25 E-1	1.7	5.5	-1.6843	2.8503	Y	G	8.22 E-4	1.39 E+5	4.19 E+5	-1.5974	19.0897	G

TIGR	PGN	LANL	Description	PP PPC / Pg Spectral Count: q-value	PP (Norm)	PPC (Norm)	log_2Ratio	log_2Sum	Regulation	PP PPC/ Pg Protein Intensity: Regulation	q-value	PP (Norm)	PPC (Norm)	log_2Ratio	log_2Sum	33277 Consensus
	PGN0493			4.76 E-2	8.6	1.5	2.5121	3.3301	Y	R	4 E-4	1.71 E+6	1.39 E+5	3.6226	20.8158	R
PG1616	PGN0496	PG1414	conserved hypothetical protein	5.89 E-1	1.7	1	0.7751	1.4390	Y	R	0	5.02 E+5	1 E+3	8.9711	18.9397	R
PG1615	PGN0497	PG1413	frdA fumarate reductase flavoprotein subunit	1.57 E-1	121.5	96.5	0.3324	7.7682	Y	Y	1.78 E-1	7.65 E+7	7.82 E+7	-0.0313	27.2056	Y
PG1614	PGN0498	PG1412	frdB fumarate reductase iron-sulfur protein	6.69 E-1	41.1	39.5	0.0563	6.3322	Y	Y	4.54 E-2	3.73 E+7	5.12 E+7	-0.4578	26.3989	Y
PG1613	PGN0499	PG1411	glyoxalase family protein	3.93 E-1	129.2	114.5	0.1743	7.9290	Y	R	4.83 E-4	6.69 E+7	3.96 E+7	0.7560	26.6669	R
PG1612	PGN0500	PG1409	mmdA methylmalonyl-CoA decarboxylase alpha subunit	1.47 E-6	241.3	140	0.7854	8.5748	R	R	0	2.94 E+8	9.57 E+7	1.6211	28.5397	R
PG1611	PGN0501	PG1408	hypothetical protein PG_1611	3.84 E-4	35.1	9.5	1.8847	5.4784	R	R	0	2.17 E+7	4.35 E+6	2.3184	24.6351	R
PG1610	PGN0502		hypothetical protein PG_1610	9.44 E-2	6.0	1	2.5825	2.8052	Y	R	4.3 E-7	3.63 E+6	1 E+3	11.8252	21.7914	R
PG1609	PGN0503	PG1407	mmdC methylmalonyl-CoA decarboxylase gamma subunit	8.35 E-3	68.5	37.5	0.8682	6.7273	R	R	1.16 E-7	1.3 E+8	1.57 E+7	3.0440	27.1167	R
PG1608	PGN0504	PG1406	mmdB methylmalonyl-CoA decarboxylase beta subunit	6.85 E-1	4.3	4.5	-0.0729	3.1339	Y	Y	2.02 E-2	9.43 E+5	2.32 E+6	-1.2998	21.6379	Y
PG1605	PGN0508	PG1404	pepC aminopeptidase C	1.91 E-1	104.4	83	0.3308	7.5499	Y	Y	9.88 E-2	9.36 E+7	1.07 E+8	-0.1890	27.5782	Y
PG1604	PGN0509	PG1403	immunoreactive 84 kDa antigen PG93	3.53 E-3	12.0	34	-1.5050	5.5229	G	Y	1.11 E-1	1.79 E+7	1.32 E+7	0.4329	24.8903	Y
PG1603	PGN0510	PG1402	HAM1 protein	6.03 E-1	17.1	15	0.1901	5.0051	Y	Y	7.26 E-2	6.73 E+6	5.46 E+6	0.3021	23.5382	Y
PG1602	PGN0511	PG1401	conserved hypothetical protein	4.4 E-1	1.7	3.5	-1.0323	2.3816	Y	Y	3.83 E-3	1.85 E+3	1.47 E+6	-9.6336	20.4891	Y
PG1601	PGN0512	PG1400	biotin--acetyl-CoA-carboxylase ligase	3.6 E-1	2.6	5.5	-1.0994	3.0120	Y	Y	8.33 E-3	2.02 E+5	1.96 E+6	-3.2761	21.0421	Y
PG1597	PGN0516	PG1396	DnaK suppressor protein putative	6 E-1	16.3	18.5	-0.1864	5.1192	Y	Y	2.03 E-2	1.99 E+7	1.13 E+7	0.8103	24.8942	Y
PG1596	PGN0517	PG1395	isoleucyl-tRNA synthetase putative	2.31 E-15	150.6	40	1.9126	7.5744	R	R	0	7.62 E+7	1.73 E+7	2.1416	26.4787	R

TIGR		PP PPC / Pg Spectral Count						PP PPC/ Pg Protein Intensity						33277
PGN	***LANL***	*q*-value	PP (Norm)	PPC (Norm)	log_2Ratio	log_2Sum	Regulation		*q*-value	PP (Norm)	PPC (Norm)	log_2Ratio	log_2Sum	Consensus
PG1595		9.45 E-12	29.9	113.5	-1.9222	7.1644	G	G	1.29 E-11	2.8 E+6	7.15 E+6	-1.3549	23.2464	G
PGN0518	*PG1394*	*rpe ribulose-phosphate 3-epimerase*												
PG1592		4.53 E-1	9.4	13	-0.4659	4.4862	Y	Y	3.4 E-2	4.31 E+6	3.32 E+6	0.3779	22.8627	Y
PGN0521	*PG1391*	*HDIG domain protein*												
PG1589		6 E-1	4.3	5.5	-0.3624	3.2896	Y	Y	3.81 E-2	2.18 E+6	3.72 E+6	-0.7715	22.4936	Y
PGN0522	*PG1389*	*folP dihydropteroate synthase*												
PG1587		6.69 E-1	6.0	5.5	0.1230	3.5223	Y	Y	1.26 E-2	3.11 E+5	4.18 E+5	-0.4276	19.4761	Y
PGN0524	*PG1387*	*PAP2 superfamily protein*												
PG1586		6.69 E-1	1.7	1.5	0.1901	1.6832	Y	G	0	1.85 E+3	7.26 E+5	-8.6159	19.4733	Y
PGN0525	*PG1386*	*batE batE protein*												
PG1585		6.7 E-1	5.1	5.5	-0.0994	3.4106	Y	Y	8.05 E-3	9.87 E+5	2.77 E+6	-1.4919	21.8428	Y
PGN0526	*PG1385*	*batD batD protein*												
PG1584		1.06 E-1	6.8	1.5	2.1901	3.0610	Y	R	5.87 E-11	3.32 E+6	4.43 E+5	2.9045	21.8419	R
PGN0527	*PG1384*	*batC batC protein*												
PG1582		4.23 E-1	2.6	1	1.3601	1.8347	Y	R	0	1.17 E+6	1 E+3	10.1967	20.1637	R
PGN0529	*PG1382*	*batA batA protein*												
PG1581		2.93 E-1	6.8	3	1.1901	3.2994	Y	Y	1.43 E-1	3.2 E+6	2.44 E+6	0.3905	22.4267	Y
PGN0530	*PG1381*	*hypothetical protein PG_1581*												
PG1580		2.99 E-1	16.3	10	0.7011	4.7147	Y	R	1.55 E-5	2.11 E+6	7.91 E+5	1.4184	21.4694	R
PGN0531	*PG1380*	*conserved hypothetical protein*												
PG1579		4.08 E-3	38.5	15	1.3601	5.7416	R	R	9.22 E-8	2.25 E+7	1.12 E+7	1.0072	25.0038	R
PGN0532	*PG1379*	*ATPase MoxR family*												
PG1578		2.01 E-2	8.6	23.5	-1.4576	5.0025	Y	G	1.15 E-5	1.84 E+6	5.62 E+6	-1.6112	22.8301	G
PGN0533	*PG1378*	*nadA quinolinate synthetase complex subunit A*												
PG1577		5.8 E-4	2.6	19.5	-2.9253	4.4638	G	G	4.51 E-6	1.35 E+6	1.21 E+7	-3.1670	23.6795	G
PGN0534	*PG1377*	*nadC nicotinate-nucleotide pyrophosphorylase*												
PG1576		7.06 E-1	6.0	6	-0.0025	3.5837	Y	Y	1.58 E-1	7.12 E+5	9.2 E+5	-0.3685	20.6381	Y
PGN0535	*PG1376*	*nadB L-aspartate oxidase*												
PG1573		7.56 E-2	1.7	8	-2.2249	3.2797	Y	G	9.68 E-8	1.85 E+3	8.36 E+5	-8.8202	19.6771	G
PGN0537	*PG1375*	*transcriptional regulator Crp family*												
PG1566		3.83 E-2	50.5	29	0.7998	6.3126	Y	R	2.71 E-12	2.23 E+7	8.44 E+6	1.4018	24.8737	R
PGN0543	*PG1371*	*gltX glutamyl-tRNA synthetase*												
PG1565		6.69 E-1	12.0	13	-0.1180	4.6427	Y	R	5.17 E-8	5.91 E+6	7.88 E+5	2.9069	22.6753	Y
PGN0544	*PG1370*	*3-deoxy-D-manno-octulosonic-acid transferase putative*												

TIGR		PP PPC / Pg Spectral Count						PP PPC/ Pg Protein Intensity						33277
PGN	***LANL***	q-value	PP (Norm)	PPC (Norm)	log_2Ratio	log_2Sum	Regulation		q-value	PP (Norm)	PPC (Norm)	log_2Ratio	log_2Sum	Consensus
PG1564		6.97 E-1	2.6	2.5	0.0381	2.3411	Y	Y	1.62 E-1	3.13 E+5	3.51 E+5	-0.1682	19.3410	Y
PGN0545	*PG1369*	*membrane protein putative*												
PG1563		3.82 E-1	21.4	15.5	0.4648	5.2052	Y	Y	6.32 E-2	8.32 E+6	1.04 E+7	-0.3228	24.1585	Y
PGN0546	*PG1368*	*rfbA glucose-1-phosphate thymidylyltransferase*												
PG1562		3.05 E-1	15.4	9.5	0.6971	4.6382	Y	Y	1.99 E-1	4.25 E+6	4.22 E+6	0.0085	23.0145	Y
PGN0547	*PG1367*	*rfbC dTDP-4-dehydrorhamnose 35-epimerase*												
PG1561		6.69 E-1	8.6	8	0.0970	4.0493	Y	Y	5.24 E-2	2.78 E+6	4.19 E+6	-0.5921	22.7327	Y
PGN0548	*PG1366*	*rfbD dTDP-4-dehydrorhamnose reductase*												
PG1560		1.98 E-1	11.1	5	1.1536	4.0111	Y	R	4.83 E-11	5.51 E+6	1.3 E+6	2.0823	22.7002	R
PGN0549	*PG1365*	*rfbB dTDP-glucose 46-dehydratase*												
PG1559		6.64 E-1	181.4	177	0.0354	8.4854	Y	Y	4.14 E-2	1.58 E+8	1.35 E+8	0.2189	28.1258	Y
PGN0550	*PG1364*	*gcvT glycine cleavage system T protein*												
PG1555		9.55 E-3	10.3	1	3.3601	3.4941	Y	R	0	9.92 E+5	1 E+3	9.9547	19.9219	R
PGN0554	*PG1361*	*conserved domain protein*												
PG1553		2.28 E-9	96.7	206.5	-1.0947	8.2441	G	Y	1.15 E-3	5.69 E+7	1.54 E+8	-1.4331	27.6497	G
PGN0556	*PG1359*	*CobN/magnesium chelatase family protein*												
PG1552		2.12 E-12	76.2	196.5	-1.3676	8.0909	G	G	7.85 E-5	2.35 E+7	3.88 E+7	-0.7223	25.8936	G
PGN0557	*PG1358*	*hmuR TonB-dependent receptor HmuR*												
PG1551		1.4 E-1	158.3	191.5	-0.2747	8.4504	Y	Y	2.98 E-3	1.26 E+8	2 E+8	-0.6689	28.2825	Y
PGN0558	*PG1357*	*hmuY hmuY protein*												
		5.44 E-2	14.5	29.5	-1.0201	5.4609	Y	G	1.65 E-6	3.71 E+6	1.17 E+7	-1.6561	23.8771	G
PGN0561														
PG1545		3.06 E-6	195.9	309.5	-0.6595	8.9814	G	Y	1.24 E-1	2.36 E+8	2.61 E+8	-0.1482	28.8874	G
PGN0564	*PG1354*	*sodB superoxide dismutase Fe-Mn*												
PG1543		1.21 E-2	25.7	9	1.5121	5.1156	Y	R	4.83 E-7	9.99 E+6	4.43 E+6	1.1729	23.7812	R
PGN0566	*PG1350*	*thioesterase family protein*												
PG1542		6 E-1	1.7	2.5	-0.5468	2.0743	Y	G	1.27 E-4	1.85 E+3	8.85 E+5	-8.9009	19.7576	G
PGN0567	*PG1349*	*prtC collagenase*												
PG1541		2.93 E-1	8.6	14.5	-0.7610	4.5271	Y	Y	1.42 E-1	3.03 E+6	2.7 E+6	0.1617	22.4501	Y
PGN0568	*PG1348*	*folK 2-amino-4-hydroxy-6-hydroxymethyldihydropteridine pyrophosphokinase*												
PG1540		1.63 E-1	35.1	22.5	0.6408	5.8475	Y	Y	8.49 E-2	1.69 E+7	1.45 E+7	0.2145	24.9039	Y
PGN0569	*PG1347*	*queA S-adenosylmethionine:tRNA ribosyltransferase-isomerase*												
PG1539		3.05 E-1	3.4	1	1.7751	2.1449	Y	R	4.74 E-9	8.59 E+5	1 E+3	9.7459	19.7133	R
PGN0570	*PG1346*													

TIGR / *PGN*	*LANL*	*q*-value	PP (Norm)	PPC (Norm)	log_2Ratio	log_2Sum	Regulation		*q*-value	PP (Norm)	PPC (Norm)	log_2Ratio	log_2Sum	33277 Consensus
		PP PPC / Pg Spectral Count						PP PPC/ Pg Protein Intensity						
PG1536		6.97 E-1	3.4	3.5	-0.0323	2.7913	Y	Y	8.62 E-2	1.38 E+6	9.88 E+5	0.4817	21.1752	Y
PGN0573	*PG1343*	*cell division protein FtsX putative*												
		4.23 E-1	2.6	1	1.3601	1.8347	Y	R	0	6 E+5	1 E+3	9.2289	19.1971	R
PGN0580														
PG1519		1.45 E-1	5.1	1	2.3601	2.6168	Y	R	1.11 E-5	1.06 E+6	1 E+3	10.0564	20.0235	R
PGN0583	*PG1329*	*hypothetical protein PG_1519*												
		3.64 E-1	18.8	13	0.5341	4.9921	Y	R	3.19 E-5	1.01 E+7	4.94 E+6	1.0349	23.8447	R
PGN0590														
PG1286		2.72 E-1	320.9	288.5	0.1534	9.2512	Y	Y	1.73 E-1	4.64 E+8	4.45 E+8	0.0595	29.7603	Y
PGN0604	*PG1133*	*ftn ferritin*												
PG1285		4.4 E-1	1.7	3.5	-1.0323	2.3816	Y	G	1.16 E-8	1.85 E+3	3.18 E+5	-7.4229	18.2850	G
PGN0606	*PG1132*	*glucosamine-6-phosphate isomerase putative*												
PG1283		5.25 E-3	125.8	181.5	-0.5291	8.2634	G	Y	2.29 E-3	6.88 E+7	9.27 E+7	-0.4302	27.2665	G
PGN0607	*PG1131*	*conserved hypothetical protein*												
PG1281		3.52 E-1	12.8	8	0.6820	4.3809	Y	Y	6.91 E-2	4.12 E+6	3.33 E+6	0.3052	22.8284	Y
PGN0609	*PG1129*	*hypothetical protein PG_1281*												
PG1280		0	227.6	82.5	1.4641	8.2766	R	R	0	2.32 E+8	5.83 E+7	1.9932	28.1137	R
PGN0610	*PG1128*	*conserved hypothetical protein*												
PG1279		0	311.5	78.5	1.9883	8.6072	R	R	0	1.74 E+8	3.19 E+7	2.4474	27.6161	R
PGN0611	*PG1127*	*D-isomer specific 2-hydroxyacid dehydrogenase family protein*												
PG1278		2.1 E-10	590.4	845.5	-0.5181	10.4877	G	G	4.9 E-4	6.97 E+8	9.92 E+8	-0.5090	30.6535	G
PGN0612	*PG1126*	*serC phosphoserine aminotransferase*												
PG1277		3.58 E-1	6.8	3.5	0.9677	3.3709	Y	Y	6.85 E-3	3.9 E+6	2.08 E+6	0.9058	22.5114	Y
PGN0613	*PG1125*	*UDP-glucose-6 dehydrogenase putative*												
		6.69 E-1	1.7	2	-0.2249	1.8919	Y	Y	1.26 E-2	1.85 E+3	3.9 E+5	-7.7202	18.5807	Y
PGN0614														
		3.78 E-2	34.2	17	1.0096	5.6788	Y	R	1.35 E-10	7.49 E+6	1.6 E+6	2.2242	23.1164	R
PGN0616														
PG0571		2.28 E-2	51.3	28	0.8746	6.3100	Y	R	5.03 E-5	1.7 E+7	7.84 E+6	1.1132	24.5633	R
PGN0618	*PG0514*	*asd aspartate-semialdehyde dehydrogenase*												
PG0573		2.22 E-1	2.6	7	-1.4473	3.2581	Y	Y	3.28 E-2	1.88 E+6	1.52 E+6	0.3058	21.6972	Y
PGN0620	*PG0515*	*mraW S-adenosyl-methyltransferase MraW*												
PG0575		2.15 E-1	4.3	1	2.0970	2.4001	Y	R	1.22 E-11	9.61 E+5	1 E+3	9.9082	19.8755	R
PGN0622	*PG0517*	*penicillin-binding protein 2 putative*												

TIGR		PP PPC / Pg Spectral Count							PP PPC/ Pg Protein Intensity					33277
PGN	***LANL***	*q*-value	PP (Norm)	PPC (Norm)	log_2Ratio	log_2Sum	Regulation		*q*-value	PP (Norm)	PPC (Norm)	log_2Ratio	log_2Sum	Consensus
PG0576		1.03 E-1	6.8	16	-1.2249	4.5138	Y	Y	1.47 E-3	2.36 E+6	5.79 E+6	-1.2979	22.9581	Y
PGN0623	*PG0518*	*murE UDP-N-acetylmuramoylalanyl-D-glutamyl-2 6-diaminopimelate ligase*												
PG0577		5.17 E-1	1.7	3	-0.8099	2.2361	Y	G	9.22 E-8	1.85 E+3	8.75 E+5	-8.8851	19.7419	G
PGN0624	*PG0519*	*mraY phospho-N-acetylmuramoyl-pentapeptide-transferase*												
PG0578		4.53 E-1	10.3	14	-0.4473	4.6010	Y	Y	2.2 E-3	1.64 E+7	2.56 E+6	2.6837	24.1788	Y
PGN0625	*PG0520*	*murD UDP-N-acetylmuramoylalanine--D-glutamate ligase*												
PG0580		8.52 E-2	3.4	11	-1.6843	3.8503	Y	G	8.6 E-4	6.77 E+5	2.25 E+6	-1.7307	21.4796	G
PGN0627	*PG0522*	*murG UDP-N-acetylglucosamine--N-acetylmuramyl-(pentapeptide) pyrophosphoryl-undecaprenol N-acetylglucosamine transferase*												
PG0581		2.15 E-1	13.7	7	0.9677	4.3709	Y	R	3.84 E-4	7.11 E+6	2.76 E+6	1.3653	23.2345	R
PGN0628	*PG0523*	*murC UDP-N-acetylmuramate--alanine ligase*												
PG0583		8.63 E-2	47.9	30	0.6756	6.2839	Y	Y	2.78 E-2	1.5 E+7	1.13 E+7	0.4144	24.6490	Y
PGN0630	*PG0525*	*ftsA cell division protein FtsA*												
PG0584		4.23 E-1	19.7	25.5	-0.3738	5.4976	Y	Y	1.53 E-1	1.09 E+7	1.16 E+7	-0.0845	24.4208	Y
PGN0631	*PG0526*	*ftsz cell division protein FtsZ*												
PG0585		6.69 E-1	7.7	8.5	-0.1424	4.0180	Y	Y	1.11 E-1	4.89 E+6	4.23 E+6	0.2114	23.1202	Y
PGN0632	*PG0527*	*YqeY family protein*												
PG0588		4.92 E-1	9.4	12.5	-0.4093	4.4537	Y	Y	5.98 E-2	6.53 E+6	1.02 E+7	-0.6379	23.9934	Y
PGN0634	*PG0530*	*panB 3-methyl-2-oxobutanoate hydroxymethyltransferase*												
PG0589		2.19 E-5	82.1	33	1.3157	6.8473	R	R	0	3.97 E+7	1.2 E+7	1.7285	25.6232	R
PGN0635	*PG0531*	*guaA GMP synthase*												
PG0592		8.11 E-5	32.5	6.5	2.3226	5.2859	R	R	3.06 E-13	2.44 E+7	4.06 E+6	2.5866	24.7601	R
PGN0636	*PG0534*	*rpmE ribosomal protein L31*												
PG0593		3.9 E-1	61.6	51.5	0.2585	6.8215	Y	Y	3 E-2	3.75 E+7	2.38 E+7	0.6600	25.8696	Y
PGN0637	*PG0535*	*htrA htrA protein*												
PG0594		2.32 E-4	19.7	2	3.2987	4.4383	R	R	6.99 E-7	3.79 E+6	7.37 E+5	2.3648	22.1116	R
PGN0638	*PG0536*	*rpoD RNA polymerase sigma-70 factor*												
PG0595		5.73 E-11	53.1	4.5	3.5594	5.8468	R	R	0	3.74 E+7	2.17 E+6	4.1047	25.2388	R
PGN0639	*PG0537*	*rpsF ribosomal protein S6*												
PG0596		1.84 E-1	49.6	35	0.5038	6.4031	Y	Y	1.74 E-2	3.69 E+7	2.69 E+7	0.4525	25.9277	Y
PGN0640	*PG0538*	*rpsR ribosomal protein S18*												
PG0597		6.23 E-9	43.6	4	3.4475	5.5740	R	R	2.09 E-8	3.93 E+7	2.62 E+6	3.9081	25.3224	R
PGN0641	*PG0540*	*rplI ribosomal protein L9*												
PG0598		6.69 E-1	1.7	2	-0.2249	1.8919	Y	R	0	4.96 E+5	2.29 E+5	1.1164	19.4670	Y
PGN0642	*PG0541*	*hypothetical protein PG_0598*												

TIGR		PP PPC / Pg Spectral Count						PP PPC/ Pg Protein Intensity						33277
PGN	LANL	q-value	PP (Norm)	PPC (Norm)	log_2Ratio	log_2Sum	Regulation	Regulation	q-value	PP (Norm)	PPC (Norm)	log_2Ratio	log_2Sum	Consensus
PG0599		4.65 E-1	76.2	85.5	-0.1670	7.3368	Y	Y	5.19 E-2	5.44 E+7	7.06 E+7	-0.3765	26.8969	Y
PGN0643	PG0542	ribBA 34-dihydroxy-2-butanone 4-phosphate synthase/GTP cyclohydrolase II												
PG0602		9.33 E-12	8.6	67.5	-2.9798	6.2490	G	G	3.63 E-13	2.19 E+6	2.26 E+7	-3.3685	24.5623	G
PGN0645	PG0543	hypothetical protein PG_0602												
PG0603		9.55 E-3	10.3	1	3.3601	3.4941	Y	R	0	4.66 E+6	1 E+3	12.1866	22.1527	R
PGN0646	PG0544	cmk cytidylate kinase												
PG0604		5.17 E-1	1.7	3	-0.8099	2.2361	Y	G	7.52 E-4	1.85 E+3	3.25 E+5	-7.4575	18.3194	G
PGN0647	PG0545	ispH hydroxymethylbutenyl pyrophosphate reductase												
PG0611		3.82 E-1	53.9	64.5	-0.2589	6.8876	Y	Y	8.76 E-3	2.54 E+7	4.53 E+7	-0.8364	26.0739	Y
PGN0654	PG0550	lipoprotein putative												
PG0613		2.25 E-1	1.7	5.5	-1.6843	2.8503	Y	Y	1.99 E-2	1.85 E+3	1.3 E+6	-9.4556	20.3114	Y
PGN0656	PG0551	hypothetical protein PG_0613												
PG0614		2.6 E-2	6.0	18.5	-1.6270	4.6141	Y	Y	1.5 E-3	4.76 E+6	1.51 E+7	-1.6634	24.2411	Y
PGN0657	PG0552	hypothetical protein PG_0614												
PG0615		5.89 E-1	19.7	17	0.2112	5.1969	Y	Y	7.7 E-2	7.4 E+6	5.82 E+6	0.3454	23.6560	Y
PGN0658	PG0553	typA GTP-binding protein TypA												
PG0616		0	367.1	659.5	-0.8453	10.0036	G	Y	5.12 E-3	5.06 E+8	6.8 E+8	-0.4273	30.1434	G
PGN0659	PG0554	thioredoxin putative												
PG0618		1.38 E-6	140.3	66	1.0883	7.6888	R	R	7.42 E-6	1.84 E+8	1 E+8	0.8746	28.0794	R
PGN0660	PG0556	alkyl hydroperoxide reductase C subunit												
PG0619		9.3 E-2	18.8	8.5	1.1471	4.7721	Y	Y	1.06 E-1	3.66 E+6	4.35 E+6	-0.2499	22.9334	Y
PGN0661	PG0557	alkyl hydroperoxide reductase F subunit												
PG0620		1.17 E-2	73.6	42.5	0.7920	6.8591	Y	R	0	2.84 E+7	7.83 E+6	1.8616	25.1122	R
PGN0662	PG0558	lon ATP-dependent protease La												
PG0621		4.75 E-1	12.8	9.5	0.4341	4.4812	Y	Y	2.39 E-3	4.33 E+6	2.59 E+6	0.7412	22.7223	Y
PGN0663	PG0559	conserved hypothetical protein												
PG0622		3.76 E-1	6.0	3	0.9975	3.1683	Y	Y	1.52 E-1	2.08 E+6	1.81 E+6	0.1990	21.8943	Y
PGN0664	PG0560	hypothetical protein PG_0622												
PG0623		4.23 E-1	361.9	339.5	0.0923	9.4542	Y	G	2.73 E-4	2.01 E+8	3.94 E+8	-0.9721	29.1476	Y
PGN0665	PG0561	tpiA triosephosphate isomerase												
PG0624		9.44 E-2	6.0	1	2.5825	2.8052	Y	R	0	1.7 E+6	1 E+3	10.7313	20.6980	R
PGN0666	PG0562	hypothetical protein PG_0624												
PG0625		4.75 E-1	18.0	14	0.3601	4.9986	Y	Y	6.08 E-2	7.13 E+6	2.12 E+7	-1.5689	24.7536	Y
PGN0667	PG0563	folE GTP cyclohydrolase I												

TIGR	PGN	LANL	Description	PP PPC / Pg Spectral Count q-value	PP (Norm)	PPC (Norm)	log_2Ratio	log_2Sum	Regulation	PP PPC/ Pg Protein Intensity	q-value	PP (Norm)	PPC (Norm)	log_2Ratio	log_2Sum	33277 Consensus
PG0627	PGN0668	PG0565	RNA-binding protein	2.4 E-1	20.5	12.5	0.7162	5.0460	Y	R	1.77 E-10	1.8 E+7	6.25 E+6	1.5220	24.5291	R
	PGN0669			6.54 E-1	2.6	2	0.3601	2.1912	Y	Y	1.17 E-2	1.04 E+6	1.44 E+6	-0.4646	21.2430	Y
PG0629	PGN0670	PG0568	ppnK ATP-NAD kinase	4.75 E-1	3.4	5.5	-0.6843	3.1575	Y	Y	1.36 E-3	8.6 E+5	3.29 E+6	-1.9333	21.9834	Y
PG0630	PGN0671	PG0569	pdxJ pyridoxal phosphate biosynthetic protein PdxJ	5.99 E-2	32.5	53	-0.7049	6.4181	Y	Y	7.1 E-2	1.73 E+7	2 E+7	-0.2131	25.1515	Y
PG0631	PGN0672	PG0570	MotA/TolQ/ExbB proton channel family protein	9.84 E-2	17.1	7.5	1.1901	4.6214	Y	Y	1.44 E-1	8.39 E+6	7.1 E+6	0.2407	23.8844	Y
PG0632	PGN0673	PG0571	biopolymer transport protein ExbD putative	1.31 E-3	13.7	1	3.7751	3.8768	Y	R	0	3.82 E+6	1 E+3	11.8991	21.8653	R
PG0633	PGN0674	PG0573	hypothetical protein PG_0633	6 E-1	1.7	2.5	-0.5468	2.0743	Y	G	2.36 E-4	1.85 E+3	9.73 E+5	-9.0384	19.8948	G
PG0634	PGN0675	PG0575	ThiJ/PfpI family protein	4.86 E-2	33.4	17	0.9730	5.6545	Y	Y	1.23 E-1	1.76 E+7	2.29 E+7	-0.3833	25.2719	Y
PG0637	PGN0678	PG0578	thiL thiamine monophosphate kinase	3.93 E-1	4.3	7.5	-0.8099	3.5581	Y	G	8.35 E-5	2.4 E+5	4.31 E+5	-0.8461	19.3544	G
PG0638	PGN0679	PG0579	lpxK tetraacyldisaccharide 4'-kinase	6 E-1	6.8	5.5	0.3157	3.6259	Y	Y	2.71 E-3	3.97 E+6	4.36 E+5	3.1872	22.0702	Y
PG0639	PGN0680	PG0580	sppA signal peptide peptidase SppA 67K type	4.31 E-1	46.2	54.5	-0.2382	6.6540	Y	Y	2.16 E-3	3.08 E+7	4.56 E+7	-0.5675	26.1865	Y
	PGN0683			2.4 E-2	203.6	260	-0.3525	8.8569	Y	G	6.19 E-5	1.06 E+8	1.69 E+8	-0.6745	28.0328	G
PG0645	PGN0684	PG0583	conserved domain protein	3.82 E-1	3.4	6.5	-0.9253	3.3107	Y	Y	1.51 E-2	1.21 E+6	3.2 E+6	-1.4038	22.0714	Y
PG0648	PGN0687	PG0586	iron compound ABC transporter periplasmic iron compound-binding protein putative	4.4 E-1	1.7	3.5	-1.0323	2.3816	Y	G	2.98 E-9	1.85 E+3	4.24 E+5	-7.8408	18.7008	G
PG0651	PGN0690	PG0589	HDIG domain protein	1.84 E-1	1.7	6	-1.8099	2.9470	Y	G	9.27 E-6	1.85 E+3	1.16 E+6	-9.2937	20.1497	G
PG0652	PGN0691	PG0590	conserved hypothetical protein	1.06 E-1	22.2	37	-0.7339	5.8887	Y	Y	5.24 E-2	4.65 E+6	6.28 E+6	-0.4337	23.3822	Y
PG0653	PGN0692	PG0591	serB phosphoserine phosphatase	7.86 E-6	39.4	7.5	2.3918	5.5503	R	R	5.38 E-8	1.05 E+7	4.15 E+6	1.3363	23.8017	R

TIGR		PP PPC / Pg Spectral Count							PP PPC/ Pg Protein Intensity					33277
PGN	*LANL*	*q*-value	PP (Norm)	PPC (Norm)	log_2Ratio	log_2Sum	Regulation		*q*-value	PP (Norm)	PPC (Norm)	log_2Ratio	log_2Sum	Consensus
PG0654		3.58 E-1	33.4	42.5	-0.3489	6.2455	Y	Y	2.53 E-3	3.24 E+7	6.24 E+7	-0.9451	26.4990	Y
PGN0693	*PG0592*	*hypothetical protein PG_0654*												
PG0658		1.54 E-1	10.3	19.5	-0.9253	4.8957	Y	G	4.01 E-4	3.94 E+6	1.08 E+7	-1.4525	23.8123	G
PGN0696	*PG0594*	*phosphatase YrbI family*												
PG0659		5.17 E-1	1.7	3	-0.8099	2.2361	Y	Y	2.95 E-2	1.85 E+3	7.7 E+5	-8.6999	19.5570	Y
PGN0697	*PG0595*	*conserved hypothetical protein*												
PG0660		3.82 E-1	1.7	4	-1.2249	2.5138	Y	G	2.06 E-6	6.51 E+5	2.99 E+6	-2.2023	21.7978	G
PGN0698	*PG0596*	*nitroreductase family protein*												
PG0664		6.27 E-1	22.2	24.5	-0.1392	5.5468	Y	R	2.9 E-4	8.14 E+6	4.19 E+6	0.9604	23.5555	Y
PGN0700	*PG0597*	*oxidoreductase Gfo/Idh/MocA family*												
PG0665		9.53 E-2	7.7	17.5	-1.1843	4.6554	Y	G	1.36 E-4	8.77 E+5	3.74 E+6	-2.0927	22.1390	G
PGN0701	*PG0598*	*lacZ-1 beta-galactosidase*												
		5.95 E-1	6.8	8.5	-0.3124	3.9397	Y	Y	1.74 E-1	1.84 E+6	1.97 E+6	-0.0991	21.8614	Y
PGN0702														
		4.75 E-1	6.8	4.5	0.6052	3.5040	Y	R	1.29 E-4	2.55 E+6	9.46 E+5	1.4277	21.7355	R
PGN0703														
PG0668		0	261.8	502.5	-0.9405	9.5781	G	G	3.56 E-6	1.05 E+8	1.94 E+8	-0.8816	28.1542	G
PGN0704	*PG0601*	*TonB-dependent receptor*												
PG0669		4.32 E-5	32.5	79.5	-1.2899	6.8075	G	G	7.69 E-11	1.76 E+7	6.67 E+7	-1.9187	26.3297	G
PGN0705	*PG0602*	*fetB heme-binding protein FetB*												
PG0670		4.53 E-1	8.6	12	-0.4879	4.3615	Y	G	5.33 E-4	3.83 E+6	7.16 E+6	-0.9048	23.3898	G
PGN0706	*PG0603*	*lipoprotein putative*												
PG0672		6 E-1	1.7	2.5	-0.5468	2.0743	Y	G	0	1.85 E+3	1.75 E+5	-6.5627	17.4316	G
PGN0708	*PG0605*	*iron compound ABC transporter ATP-binding protein*												
PG0674		6.61 E-8	59.9	12.5	2.2605	6.1778	R	R	0	6.21 E+7	8.06 E+6	2.9456	26.0648	R
PGN0709	*PG0606*	*iorB indolepyruvate ferredoxin oxidoreductase beta subunit*												
PG0675		9.88 E-4	118.1	68	0.7962	7.5398	R	R	7.88 E-8	1.06 E+8	4.93 E+7	1.1068	27.2109	R
PGN0710	*PG0607*	*iorA indolepyruvate ferredoxin oxidoreductase alpha subunit*												
PG0676		3.58 E-1	33.4	42.5	-0.3489	6.2455	Y	Y	5.24 E-3	2.13 E+7	3.25 E+7	-0.6108	25.6798	Y
PGN0711	*PG0608*	*oxidoreductase short chain dehydrogenase/reductase family*												
PG0677		7.02 E-6	47.9	11.5	2.0589	5.8928	R	R	0	3.53 E+7	3.8 E+6	3.2134	25.2192	R
PGN0713	*PG0609*	*LYS1 saccharopine dehydrogenase*												
PG0678		3.82 E-1	1.7	4	-1.2249	2.5138	Y	G	2.74 E-6	1.85 E+3	8.31 E+5	-8.8106	19.6675	G
PGN0714	*PG0610*	*pyrazinamidase/nicotinamidase putative*												

TIGR	PGN	LANL	Description	PP PPC / Pg Spectral Count q-value	PP (Norm)	PPC (Norm)	log_2Ratio	log_2Sum	Regulation	PP PPC/ Pg Protein Intensity q-value	PP (Norm)	PPC (Norm)	log_2Ratio	log_2Sum	Regulation	33277 Consensus
	PGN0721			6.69 E-1	3.4	3	0.1901	2.6832	Y	2.08 E-11	3.46 E+6	1.42 E+6	1.2812	22.2181	R	R
PG0686	PGN0722	PG0620	conserved hypothetical protein	1.45 E-1	60.8	43	0.4986	6.6970	Y	1.63 E-8	2.37 E+7	1.14 E+7	1.0583	25.0612	R	R
PG0687	PGN0723	PG0621	sucD succinate-semialdehyde dehydrogenase	8.11 E-5	926.7	1123.5	-0.2779	11.0015	G	3.86 E-2	7.79 E+8	6.94 E+8	0.1684	30.4560	Y	Y
PG0689	PGN0724	PG0622	4hbD NAD-dependent 4-hydroxybutyrate dehydrogenase	2.67 E-1	475.7	435.5	0.1275	9.8317	Y	2.01 E-1	4.04 E+8	4.04 E+8	-0.0012	29.5904	Y	Y
PG0690	PGN0725	PG0623	abfT-1 4-hydroxybutyrate CoA-transferase	1.43 E-1	280.7	324	-0.2072	9.2400	Y	1.94 E-2	2.12 E+8	2.72 E+8	-0.3595	28.8498	Y	Y
PG0691	PGN0726	PG0624	NifU-related protein	4.65 E-1	2.6	4.5	-0.8099	2.8211	Y	1.74 E-2	7.31 E+5	1.33 E+6	-0.8602	20.9721	Y	Y
PG0692	PGN0727	PG0625	abfD 4-hydroxybutyryl-CoA dehydratase	7.34 E-5	1542.7	1795.5	-0.2189	11.7049	G	2.34 E-2	1.18 E+9	1.39 E+9	-0.2342	31.2597	Y	G
PG0694	PGN0728	PG0626	immunoreactive 42 kDa antigen PG33	0	363.7	1101.5	-1.5988	10.5168	G	3.7 E-4	5.59 E+8	7.66 E+8	-0.4550	30.3032	G	G
PG0695	PGN0729	PG0627	immunoreactive 43 kDa antigen PG32	0	313.2	982	-1.6488	10.3389	G	5.77 E-5	4.91 E+8	8.23 E+8	-0.7442	30.2917	G	G
PG0698	PGN0732	PG0628	lipoprotein putative	6.05 E-2	6.8	1	2.7751	2.9718	Y	3.06 E-4	2.59 E+6	1 E+3	11.3400	21.3063	R	R
PG0699	PGN0733	PG0629	malP maltodextrin phosphorylase	2.18 E-7	41.9	109.5	-1.3850	7.2425	G	2.75 E-10	1.94 E+7	4.72 E+7	-1.2809	25.9892	G	G
PG0701	PGN0735	PG0631	cobU cobinamide kinase/cobinamide phosphate guanylyltransferase	5.43 E-1	6.0	8	-0.4175	3.8063	Y	5.81 E-2	1.46 E+6	1.21 E+6	0.2684	21.3497	Y	Y
PG0702	PGN0736	PG0632	nicotinate-nucleotide--dimethylbenzimidazole phosphoribosyltransferase putative	6 E-1	1.7	2.5	-0.5468	2.0743	Y	2.23 E-5	1.85 E+3	5.31 E+5	-8.1650	19.0237	G	G
PG0704	PGN0738	PG0634	phosphoglycerate mutase family protein	5.17 E-1	1.7	3	-0.8099	2.2361	Y	1.98 E-4	1.85 E+3	7.54 E+5	-8.6712	19.5285	G	G
PG0705	PGN0739	PG0635	murI glutamate racemase	6.78 E-1	25.7	25	0.0381	5.6630	Y	2.4 E-11	1.73 E+7	7.32 E+6	1.2406	24.5537	R	R
PG0706	PGN0740	PG0636	hypothetical protein PG_0706	4.93 E-1	7.7	10.5	-0.4473	4.1859	Y	1.08 E-1	4.71 E+6	5.68 E+6	-0.2716	23.3091	Y	Y
PG0707	PGN0741	PG0637	TonB-dependent receptor putative	0	160.9	436.5	-1.4402	9.2225	G	7.61 E-16	6.17 E+7	2.34 E+8	-1.9211	28.1370	G	G

TIGR		PP PPC / Pg Spectral Count						PP PPC/ Pg Protein Intensity						33277
PGN	*LANL*	*q*-value	PP (Norm)	PPC (Norm)	log_2Ratio	log_2Sum	Regulation		*q*-value	PP (Norm)	PPC (Norm)	log_2Ratio	log_2Sum	Consensus
PG0708		6.89 E-8	253.3	401	-0.6629	9.3537	G	Y	1.2 E-2	8.98 E+7	1.53 E+8	-0.7657	27.8540	G
PGN0742	*PG0639*	*peptidyl-prolyl cis-trans isomerase FKBP-type*												
PG0709		1.31 E-1	44.5	29	0.6176	6.1996	Y	Y	3.21 E-2	6.24 E+7	4.35 E+7	0.5201	26.6591	Y
PGN0743	*PG0640*	*fkpA peptidyl-prolyl cis-trans isomerase FkpA FKBP-type*												
PG0710		2.31 E-4	24.8	4	2.6331	4.8487	R	R	5.17 E-9	1.26 E+7	2.46 E+6	2.3504	23.8409	R
PGN0744	*PG0641*	*peptidyl-prolyl cis-trans isomerase FKBP-type*												
PG0712		4.91 E-1	10.3	13.5	-0.3948	4.5709	Y	Y	7.61 E-2	4.4 E+6	3.59 E+6	0.2942	22.9300	Y
PGN0746	*PG0642*	*hypothetical protein PG_0712*												
PG0713		4.4 E-1	1.7	3.5	-1.0323	2.3816	Y	G	5.69 E-6	1.85 E+3	3.9 E+5	-7.7184	18.5790	G
PGN0747	*PG0643*	*trpG anthranilate synthase component II*												
		5.17 E-1	73.6	66.5	0.1461	7.1302	Y	Y	4.8 E-2	3.72 E+7	3.31 E+7	0.1698	26.0665	Y
PGN0748														
PG0714		2.84 E-2	1.7	10	-2.5468	3.5498	Y	Y	3.74 E-2	7.93 E+5	1.03 E+6	-0.3812	20.8009	Y
PGN0750	*PG0644*	*cutC copper homeostasis protein CutC*												
PG0715		4.23 E-1	2.6	1	1.3601	1.8347	Y	R	0	9.82 E+5	1 E+3	9.9391	19.9064	R
PGN0751	*PG0645*	*transporter*												
PG0724		1.44 E-2	167.7	223.5	-0.4143	8.6118	Y	G	2.34 E-5	9.48 E+7	1.59 E+8	-0.7500	27.9209	G
PGN0756	*PG0652*	*prolyl oligopeptidase family protein*												
PG0725		1.45 E-1	5.1	1	2.3601	2.6168	Y	R	2.92 E-10	2.6 E+6	1 E+3	11.3430	21.3093	R
PGN0757	*PG0653*	*hydrolase haloacid dehalogenase-like family*												
PG0726		1.52 E-4	22.2	59.5	-1.4193	6.3531	G	Y	1.22 E-3	2.61 E+7	7.08 E+7	-1.4379	26.5303	G
PGN0758	*PG0654*	*lipoprotein putative*												
PG0728		6.69 E-1	9.4	10	-0.0874	4.2789	Y	Y	4.54 E-2	3.86 E+6	2.55 E+6	0.5950	22.6119	Y
PGN0759	*PG0655*	*conserved hypothetical protein*												
PG0729		6.92 E-2	3.4	11.5	-1.7485	3.8994	Y	G	1.95 E-4	8.58 E+5	3.95 E+6	-2.2028	22.1968	G
PGN0760	*PG0656*	*ddlA D-alanine--D-alanine ligase*												
PG0733		1.92 E-1	38.5	26	0.5665	6.0113	Y	R	8.36 E-4	2.24 E+7	1.31 E+7	0.7717	25.0825	R
PGN0764	*PG0661*	*ribE riboflavin synthase alpha subunit*												
PG0734		6.45 E-2	18.0	33.5	-0.8987	5.6856	Y	Y	1.51 E-2	7.66 E+6	1.24 E+7	-0.6925	24.2557	Y
PGN0765	*PG0662*	*nitroreductase family protein*												
PG0735		4.4 E-1	21.4	27	-0.3359	5.5967	Y	R	2.24 E-6	8.25 E+6	3.93 E+6	1.0690	23.5379	Y
PGN0766	*PG0663*	*aminotransferase class V*												
PG0737		1.45 E-1	5.1	1	2.3601	2.6168	Y	R	2.13 E-13	1.23 E+6	1 E+3	10.2692	20.2361	R
PGN0768	*PG0665*	*hypothetical protein PG_0737*												

TIGR		PP PPC / Pg Spectral Count						PP PPC/ Pg Protein Intensity						33277
PGN	***LANL***	*q*-value	PP (Norm)	PPC (Norm)	log_2Ratio	log_2Sum	Regulation		*q*-value	PP (Norm)	PPC (Norm)	log_2Ratio	log_2Sum	Consensus
PG0738		6.69 E-1	1.7	2	-0.2249	1.8919	Y	G	7.02 E-8	1.85 E+3	1.98 E+5	-6.7403	17.6075	G
PGN0769	*PG0666*	*cytidine/deoxycytidylate deaminase family protein*												
PG0739		3.71 E-1	9.4	5.5	0.7751	3.8984	Y	Y	1.08 E-1	1.24 E+6	1.72 E+6	-0.4731	21.4951	Y
PGN0770	*PG0667*	*metallo-beta-lactamase family protein*												
PG0745		3.64 E-1	5.1	9	-0.8099	3.8211	Y	Y	8.85 E-2	3.05 E+6	2.2 E+6	0.4697	22.3231	Y
PGN0773	*PG0673*	*lactoylglutathione lyase putative*												
PG0747		6.33 E-1	7.7	6.5	0.2446	3.8279	Y	R	1.59 E-6	1.73 E+6	5.41 E+5	1.6811	21.1173	R
PGN0775	*PG0675*	*sigma-54 dependent DNA-binding response regulator*												
PG0750		5.17 E-1	3.4	2	0.7751	2.4390	Y	R	1.5 E-4	1.15 E+6	6.66 E+5	0.7863	20.7917	R
PGN0777	*PG0676*	*glycosyl transferase group 2 family protein*												
PG0751		6.69 E-5	1.7	21	-3.6172	4.5053	G	G	5.43 E-6	1.85 E+3	6.74 E+6	-11.8297	22.6838	G
PGN0778	*PG0677*	*porT porT protein*												
PG0752		8.33 E-5	14.5	48	-1.7224	5.9668	G	G	8.31 E-6	1.03 E+7	2.63 E+7	-1.3469	25.1273	G
PGN0779	*PG0678*	*uracil phosphoribosyltransferase putative*												
PG0753		4.21 E-1	3.4	1.5	1.1901	2.2994	Y	R	9.64 E-4	1.26 E+6	1.62 E+5	2.9580	20.4364	R
PGN0780	*PG0679*	*prtQ protease*												
PG0754		1.21 E-2	25.7	9	1.5121	5.1156	Y	R	2.26 E-5	6.49 E+6	2.82 E+6	1.2007	23.1502	R
PGN0781	*PG0680*	*topA DNA topoisomerase I*												
		5.43 E-1	12.0	9.5	0.3345	4.4249	Y	Y	3.11 E-3	4.81 E+6	2.71 E+6	0.8296	22.8422	Y
PGN0783														
		3.43 E-1	24.0	17	0.4950	5.3561	Y	Y	4.1 E-3	8.27 E+6	4.87 E+6	0.7641	23.6483	Y
PGN0784														
PG0756		1.61 E-1	6.8	2	1.7751	3.1449	Y	Y	6.07 E-2	9.57 E+5	6.69 E+5	0.5163	20.6324	Y
PGN0786	*PG0682*	*conserved hypothetical protein*												
PG0758		6.96 E-1	68.5	69	-0.0115	7.1028	Y	Y	1.82 E-1	4.38 E+7	4.47 E+7	-0.0289	26.3996	Y
PGN0788	*PG0684*	*dcp-1 peptidyl-dipeptidase Dcp*												
PG0759		6.69 E-1	26.5	28	-0.0781	5.7689	Y	Y	6.34 E-2	6.25 E+6	7.75 E+6	-0.3112	23.7392	Y
PGN0789	*PG0685*	*TPR domain protein*												
PG0762		5.43 E-1	112.9	105	0.1052	7.7678	Y	R	6.82 E-10	1.12 E+8	5.52 E+7	1.0260	27.3216	R
PGN0791	*PG0687*	*trigger factor putative*												
PG0766		1.45 E-1	287.5	331	-0.2033	9.2726	Y	Y	1.96 E-1	2.58 E+8	2.59 E+8	-0.0068	28.9453	Y
PGN0792	*PG0690*	*pnpA polyribonucleotide nucleotidyltransferase*												
PG0767		1.26 E-2	19.7	42	-1.0937	5.9467	Y	Y	4.68 E-3	5.49 E+6	9.28 E+6	-0.7587	23.8161	Y
PGN0793	*PG0691*	*malQ 4-alpha-glucanotransferase*												

TIGR		PP PPC / Pg Spectral Count						PP PPC/ Pg Protein Intensity						33277
PGN	***LANL***	*q*-value	PP (Norm)	PPC (Norm)	log_2Ratio	log_2Sum	Regulation		*q*-value	PP (Norm)	PPC (Norm)	log_2Ratio	log_2Sum	Consensus
PG0768		2.14 E-1	3.4	8.5	-1.3124	3.5756	Y	Y	1.92 E-3	9.63 E+5	4.37 E+6	-2.1807	22.3461	Y
PGN0794	*PG0692*	*conserved hypothetical protein*												
PG0769		6.18 E-2	12.8	26.5	-1.0459	5.2977	Y	Y	1.35 E-1	1.57 E+7	1.23 E+7	0.3486	24.7410	Y
PGN0795	*PG0693*	*fibronectin type III domain protein*												
PG0774		6.69 E-1	1.7	1.5	0.1901	1.6832	Y	G	0	1.85 E+3	3.27 E+5	-7.4660	18.3279	Y
PGN0798	*PG0695*	*hypothetical protein PG_0774*												
PG0775		6.34 E-3	160.0	109	0.5538	8.0715	R	Y	1.86 E-3	1.12 E+8	7.09 E+7	0.6559	27.4451	R
PGN0799	*PG0696*	*acyl-CoA dehydrogenase family protein*												
PG0776		4.76 E-7	126.6	54.5	1.2164	7.5009	R	R	0	7.17 E+7	2.27 E+7	1.6614	26.4909	R
PGN0800	*PG0697*	*etfA-1 electron transfer flavoprotein alpha subunit*												
PG0777		0	124.9	10	3.6430	7.0760	R	R	0	6.12 E+7	1.21 E+7	2.3394	26.1277	R
PGN0801	*PG0698*	*etfB-1 electron transfer flavoprotein beta subunit*												
PG0778		6 E-1	1.7	2.5	-0.5468	2.0743	Y	G	1.46 E-5	1.85 E+3	8.05 E+5	-8.7657	19.6227	G
PGN0802	*PG0699*	*conserved hypothetical protein*												
PG0779		1.64 E-2	51.3	27	0.9271	6.2917	Y	R	1.37 E-4	4.49 E+7	2.27 E+7	0.9833	26.0118	R
PGN0803	*PG0700*	*hypothetical protein PG_0779*												
PG0780		3.29 E-4	89.0	43.5	1.0326	7.0497	R	R	2.45 E-9	8.66 E+7	3.59 E+7	1.2711	26.8673	R
PGN0804	*PG0701*	*hypothetical protein PG_0780*												
PG0782		5.89 E-6	242.1	145.5	0.7349	8.5986	R	Y	2.6 E-2	5.61 E+8	4.42 E+8	0.3449	29.9015	R
PGN0806	*PG0703*	*MotA/TolQ/ExbB proton channel family protein*												
PG0784		4.23 E-1	14.5	19.5	-0.4228	5.0894	Y	Y	5.26 E-3	6.58 E+6	3.97 E+6	0.7303	23.3306	Y
PGN0808	*PG0705*	*polyprenyl synthetase*												
PG0785		6.81 E-6	35.1	5.5	2.6732	5.3428	R	R	0	5.22 E+7	5.7 E+6	3.1953	25.7880	R
PGN0809	*PG0707*	*tonB protein putative*												
PG0788		1.76 E-2	6.0	19.5	-1.7030	4.6718	Y	Y	3.64 E-3	1.87 E+6	3.14 E+6	-0.7443	22.2578	Y
PGN0811	*PG0709*	*hypothetical protein PG_0788*												
PG0789		4.23 E-1	2.6	1	1.3601	1.8347	Y	R	0	4.27 E+6	1 E+3	12.0608	22.0269	R
PGN0812	*PG0710*	*conserved hypothetical protein*												
PG0790		6.05 E-2	6.8	1	2.7751	2.9718	Y	R	0	3.05 E+6	1 E+3	11.5760	21.5422	R
PGN0813	*PG0711*	*obg GTP-binding protein Obg*												
PG0791		2.92 E-4	37.6	10.5	1.8422	5.5894	R	R	0	1.62 E+7	2.34 E+6	2.7936	24.1481	R
PGN0814	*PG0712*	*adk adenylate kinase*												
PG0792		2.37 E-2	3.4	14	-2.0323	4.1229	Y	G	1.81 E-7	1.62 E+6	6.28 E+6	-1.9504	22.9142	G
PGN0815	*PG0713*	*hpt hypoxanthine phosphoribosyltransferase*												

TIGR		PP PPC / Pg Spectral Count							PP PPC/ Pg Protein Intensity					33277
PGN	*LANL*	*q*-value	PP (Norm)	PPC (Norm)	log_2Ratio	log_2Sum	Regulation		*q*-value	PP (Norm)	PPC (Norm)	log_2Ratio	log_2Sum	Consensus
PG0793		3.9 E-3	95.0	55	0.7882	7.2286	R	R	1.89 E-14	4.82 E+7	2.27 E+7	1.0861	26.0783	R
PGN0816	*PG0714*	*fbp fructose-16-bisphosphatase*												
PG0794		6.1 E-5	18.8	1	4.2345	4.3092	R	R	1.87 E-8	1.31 E+7	1 E+3	13.6786	23.6445	R
PGN0817	*PG0715*	*penicillin-binding protein 1A putative*												
PG0796		1.16 E-2	51.3	26	0.9815	6.2731	Y	R	5.14 E-16	2.15 E+7	7.05 E+6	1.6071	24.7660	R
PGN0819	*PG0717*	*leuS leucyl-tRNA synthetase*												
PG0800		3.82 E-1	3.4	6.5	-0.9253	3.3107	Y	Y	1.12 E-2	1.39 E+6	8.25 E+5	0.7522	21.0790	Y
PGN0823	*PG0719*	*conserved hypothetical protein*												
PG0801		6.55 E-1	5.1	6	-0.2249	3.4769	Y	Y	5.72 E-2	8.71 E+5	1.17 E+6	-0.4264	20.9616	Y
PGN0824	*PG0720*	*polyA polymerase family protein*												
PG0802		1.19 E-2	124.9	175	-0.4863	8.2285	Y	G	2.42 E-4	4.43 E+7	7.91 E+7	-0.8355	26.8792	G
PGN0826	*PG0721*	*pdhD alpha keto acid dehydrogenase complex E3 component lipoamide dehydrogenase*												
PG0803		4.21 E-1	6.8	10.5	-0.6172	4.1165	Y	Y	5.41 E-2	5.07 E+6	1.19 E+7	-1.2276	24.0147	Y
PGN0827	*PG0722*	*nagB glucosamine-6-phosphate isomerase*												
PG0804		2.31 E-1	94.1	75.5	0.3181	7.4062	Y	Y	7.86 E-2	4.52 E+7	5.47 E+7	-0.2757	26.5745	Y
PGN0828	*PG0723*	*flavodoxin*												
PG0805		2.74 E-1	1.7	5	-1.5468	2.7466	Y	G	4.21 E-7	3.43 E+5	2.81 E+6	-3.0377	21.5896	G
PGN0829	*PG0724*	*lgt prolipoprotein diacylglyceryl transferase*												
PG0806		3.05 E-1	20.5	29	-0.4979	5.6304	Y	Y	4.32 E-3	9.83 E+6	1.56 E+7	-0.6688	24.6012	Y
PGN0830	*PG0725*	*oxidoreductase Gfo/Idh/MocA family*												
PG0807		5.8 E-1	16.3	19	-0.2249	5.1399	Y	Y	1.73 E-1	8.13 E+6	8.59 E+6	-0.0794	23.9947	Y
PGN0831	*PG0726*	*NusB family protein*												
PG0809		2.27 E-4	21.4	57	-1.4139	6.2926	G	G	2.26 E-6	9.17 E+6	1.99 E+7	-1.1148	24.7904	G
PGN0832	*PG0727*	*hypothetical protein PG_0809*												
PG0811		3.4 E-1	12.8	19	-0.5659	4.9925	Y	Y	1.03 E-1	6.31 E+6	7.13 E+6	-0.1774	23.6798	Y
PGN0833	*PG0729*	*ruvA Holliday junction DNA helicase RuvA*												
PG1493		4.81 E-2	4.3	14	-1.7103	4.1921	Y	G	2.59 E-5	1.41 E+6	5.51 E+6	-1.9689	22.7221	G
PGN0836	*PG1308*	*hypothetical protein PG_1493*												
PG1492		3.05 E-1	3.4	1	1.7751	2.1449	Y	R	0	2.49 E+6	1 E+3	11.2818	21.2481	R
PGN0837	*PG1307*	*hypothetical protein PG_1492*												
PG1433		2.72 E-1	4.3	9	-1.0729	3.7310	Y	Y	1.03 E-2	8.47 E+5	5.09 E+6	-2.5867	22.5014	Y
PGN0840	*PG1256*	*hydrolase*												
PG1434		2.74 E-1	8.6	4	1.0970	3.6504	Y	R	3.57 E-4	2.41 E+6	1.15 E+6	1.0742	21.7623	R
PGN0841	*PG1257*	*ispD 4-diphosphocytidyl-2C-methyl-D-erythritol synthase*												

TIGR	PGN	LANL	Description	PP PPC / Pg Spectral Count q-value	PP (Norm)	PPC (Norm)	log_2Ratio	log_2Sum	Regulation (Spectral Count)	Regulation (Protein Intensity)	PP PPC/ Pg Protein Intensity q-value	PP (Norm)	PPC (Norm)	log_2Ratio	log_2Sum	33277 Consensus
PG1374	PGN0852	PG1208	*immunoreactive 47 kDa antigen PG97*	6.41 E-1	63.3	60	0.0777	6.9462	Y	Y	1.27 E-3	2.82 E+7	5.91 E+7	-1.0680	26.3798	Y
PG1378	PGN0856	PG1209	*mutY A/G-specific adenine glycosylase*	6.69 E-1	1.7	2	-0.2249	1.8919	Y	Y	9.81 E-3	1.85 E+3	6.2 E+5	-8.3882	19.2462	Y
PG1379	PGN0857	PG1210	*ABC transporter periplasmic substrate-binding protein putative*	1.06 E-1	12.8	24.5	-0.9327	5.2224	Y	G	1.11 E-4	6.02 E+6	1.67 E+7	-1.4690	24.4348	G
PG1382	PGN0860	PG1213	*hypothetical protein PG_1382*	3.15 E-9	40.2	118	-1.5530	7.3057	G	G	1.29 E-8	1.83 E+7	9.53 E+7	-2.3827	26.7587	G
	PGN0862			2.25 E-1	6.8	2.5	1.4532	3.2242	Y	Y	3.14 E-3	1.25 E+6	4.39 E+5	1.5065	20.6860	Y
	PGN0863			5.74 E-1	4.3	3	0.5121	2.8636	Y	Y	1.28 E-2	2.28 E+6	8.8 E+5	1.3727	21.5910	Y
PG1397	PGN0865	PG1228	*purH phosphoribosylaminoimidazolecarboxamide formyltransferase/IMP cyclohydrolase*	6.94 E-1	18.8	18.5	0.0251	5.2220	Y	Y	1.37 E-1	1.03 E+7	1.15 E+7	-0.1526	24.3782	Y
PG1396	PGN0866	PG1227	*mreB cell shape-determining protein MreB*	6.36 E-1	62.5	66	-0.0795	7.0052	Y	Y	1.12 E-1	3.17 E+7	3.68 E+7	-0.2171	26.0298	Y
PG1393	PGN0869	PG1224	*penicillin-binding protein 2 putative*	6.69 E-1	1.7	1.5	0.1901	1.6832	Y	G	0	1.85 E+3	1.63 E+5	-6.4615	17.3315	Y
PG1389	PGN0872	PG1221	*DNA-binding protein histone-like family*	3.82 E-1	7.7	12	-0.6399	4.3002	Y	Y	1.11 E-1	1.54 E+7	1.17 E+7	0.3980	24.6938	Y
PG1388	PGN0873	PG1220	*hypothetical protein PG_1388*	2.15 E-1	4.3	1	2.0970	2.4001	Y	Y	1.69 E-3	2.19 E+7	1 E+3	14.4218	24.3877	Y
PG1387	PGN0874	PG1218	*hypothetical protein PG_1387*	9.48 E-2	1.7	7.5	-2.1318	3.2034	Y	G	1.34 E-4	1.85 E+3	5.04 E+6	-11.4116	22.2658	G
PG1386	PGN0875	PG1217	*gyrA DNA gyrase A subunit*	3.7 E-7	111.2	44	1.3380	7.2783	R	R	7.39 E-10	7.03 E+7	2.79 E+7	1.3322	26.5491	R
PG1385	PGN0876	PG1216	*TPR domain protein*	0	282.4	90.5	1.6416	8.5425	R	R	0	3.44 E+8	9.58 E+7	1.8437	28.7120	R
	PGN0877			3.05 E-1	6.8	12	-0.8099	4.2361	Y	Y	1.24 E-1	3.08 E+6	2.61 E+6	0.2428	22.4397	Y
PG1401	PGN0880	PG1232	*beta-eliminating lyase*	5.92 E-1	566.4	581	-0.0366	10.1642	Y	Y	1.72 E-1	5.95 E+8	5.83 E+8	0.0309	30.1339	Y
PG1402	PGN0881	PG1233	*AP endonuclease domain protein*	5.17 E-1	1.7	3	-0.8099	2.2361	Y	G	0	1.85 E+3	3.73 E+5	-7.6556	18.5164	G

TIGR		PP PPC / Pg Spectral Count							PP PPC/ Pg Protein Intensity					33277
PGN	*LANL*	*q*-value	PP (Norm)	PPC (Norm)	log_2Ratio	log_2Sum	Regulation		*q*-value	PP (Norm)	PPC (Norm)	log_2Ratio	log_2Sum	Consensus
PG1405		5.47 E-2	2.6	10.5	-2.0323	3.7079	Y	G	2.6 E-6	6.84 E+5	3.46 E+6	-2.3395	21.9835	G
PGN0884	*PG1236*	*hypothetical protein PG_1405*												
PG1407		1.84 E-1	1.7	6	-1.8099	2.9470	Y	Y	5.66 E-2	6.04 E+5	7.94 E+5	-0.3948	20.4154	Y
PGN0885	*PG1237*	*nitroimidazole resistance protein putative*												
PG1408		6 E-1	1.7	2.5	-0.5468	2.0743	Y	G	0	1.85 E+3	6.38 E+5	-8.4285	19.2864	G
PGN0886	*PG1238*	*heavy metal efflux pump CzcD family*												
PG1411		6.49 E-1	4.3	3.5	0.2897	2.9594	Y	Y	3.45 E-2	8.25 E+5	3.75 E+5	1.1375	20.1939	Y
PGN0889	*PG1240*	*potassium uptake protein TrkA putative*												
PG1414		1.03 E-8	189.1	327.5	-0.7924	9.0129	G	G	7.27 E-4	1.27 E+8	1.85 E+8	-0.5458	28.2151	G
PGN0890	*PG1242*	*hypothetical protein PG_1414*												
PG1416		5.93 E-1	59.0	54.5	0.1154	6.8271	Y	Y	8.74 E-2	3.48 E+7	4.33 E+7	-0.3156	26.2174	Y
PGN0891	*PG1243*	*fabK enoyl-(acyl-carrier-protein) reductase II*												
PG1417		7.41 E-3	95.8	58	0.7245	7.2652	R	Y	1.42 E-2	5.69 E+7	4.39 E+7	0.3734	26.5873	R
PGN0893	*PG1244*	*fumB fumarate hydratase class I anaerobic*												
PG1418		6 E-1	3.4	2.5	0.4532	2.5662	Y	R	0	5.89 E+5	1.52 E+5	1.9536	19.4981	R
PGN0894	*PG1245*	*dnaX DNA polymerase III gamma and tau subunits*												
PG1422		6.69 E-1	10.3	9.5	0.1121	4.3051	Y	Y	1.68 E-1	1.92 E+6	1.74 E+6	0.1435	21.8006	Y
PGN0896	*PG1248*	*dacB D-alanyl-D-alanine carboxypeptidase*												
PG1424		6.78 E-15	367.1	623	-0.7632	9.9514	G	G	9.11 E-8	2.79 E+8	5.34 E+8	-0.9397	29.5983	G
PGN0898	*PG1249*	*peptidylarginine deiminase*												
PG1427		7.99 E-4	3.4	21	-2.6172	4.6101	G	G	1.04 E-6	1.24 E+6	8.43 E+6	-2.7619	23.2055	G
PGN0900	*PG1251*	*thiol protease/hemagglutinin PrtT precursor putative*												
PG1428		5.17 E-1	7.7	5.5	0.4856	3.7226	Y	Y	9.57 E-3	6.51 E+6	3.57 E+6	0.8681	23.2653	Y
PGN0901	*PG1252*	*ribH riboflavin synthase beta subunit*												
PG1430		6.05 E-2	118.1	86.5	0.4490	7.6765	Y	Y	1.4 E-2	1.01 E+8	7.41 E+7	0.4522	27.3861	Y
PGN0902	*PG1253*	*TPR domain protein*												
PG1431		6.64 E-1	4.3	5	-0.2249	3.2139	Y	Y	2 E-2	4.89 E+5	3.21 E+6	-2.7146	21.8186	Y
PGN0903	*PG1254*	*DNA-binding response regulator LuxR family*												
PG1432		6 E-1	4.3	5.5	-0.3624	3.2896	Y	R	3.11 E-6	2.61 E+6	4.95 E+5	2.4009	21.5682	Y
PGN0904	*PG1255*	*sensor histidine kinase*												
PG1065		9.03 E-2	14.5	27.5	-0.9188	5.3939	Y	G	7.8 E-4	5.5 E+6	1.22 E+7	-1.1450	24.0747	G
PGN0905	*PG0947*	*pyrD dihydroorotate dehydrogenase*												
PG1064		2.72 E-1	4.3	9	-1.0729	3.7310	Y	Y	1.83 E-2	7.77 E+5	1.46 E+6	-0.9097	21.0930	Y
PGN0906	*PG0946*	*dihydroorotate dehydrogenase putative*												

TIGR		PP PPC / Pg Spectral Count							PP PPC/ Pg Protein Intensity					33277
PGN	***LANL***	*q*-value	PP (Norm)	PPC (Norm)	log_2Ratio	log_2Sum	Regulation		*q*-value	PP (Norm)	PPC (Norm)	log_2Ratio	log_2Sum	Consensus
PG1063		6.97 E-1	3.4	3.5	-0.0323	2.7913	Y	R	3.67 E-4	1.57 E+6	7.42 E+5	1.0790	21.1399	Y
PGN0907	*PG0945*	*transcriptional regulator putative*												
PG1213		6.54 E-1	2.6	2	0.3601	2.1912	Y	R	6.77 E-4	1.28 E+6	3.75 E+5	1.7737	20.6618	R
PGN0911	*PG1074*	*rnhA ribonuclease H*												
PG1212		5.81 E-2	18.8	7.5	1.3276	4.7183	Y	Y	3.21 E-3	1.49 E+7	2.44 E+6	2.6109	24.0499	Y
PGN0912	*PG1073*	*TPR domain protein*												
PG1211		4.4 E-1	1.7	3.5	-1.0323	2.3816	Y	G	6.8 E-4	1.85 E+3	1.61 E+6	-9.7690	20.6244	G
PGN0913	*PG1072*	*hexapeptide transferase family protein*												
PG1210		3.92 E-4	259.3	357	-0.4615	9.2674	G	G	5.79 E-5	2.04 E+8	3.1 E+8	-0.6063	28.9384	G
PGN0914	*PG1071*	*peptidase M24 family*												
PG1209		4.23 E-1	35.9	43.5	-0.2755	6.3117	Y	Y	1.34 E-2	1.42 E+7	1.08 E+7	0.4021	24.5753	Y
PGN0915	*PG1070*	*hypothetical protein PG_1209*												
PG1208		3.05 E-1	393.6	427.5	-0.1192	9.6814	Y	Y	4.13 E-2	2.73 E+8	2.38 E+8	0.2006	28.9277	Y
PGN0916	*PG1069*	*dnaK dnaK protein*												
		7.31 E-2	18.0	33	-0.8770	5.6715	Y	Y	6.83 E-2	1.24 E+7	1.51 E+7	-0.2872	24.7149	Y
PGN0928														
		4.4 E-1	26.5	21	0.3370	5.5706	Y	R	4.95 E-5	1.52 E+7	4.78 E+6	1.6651	24.2487	R
PGN0929														
		3.82 E-1	1.7	4	-1.2249	2.5138	Y	G	2.19 E-10	1.85 E+3	2.59 E+6	-10.4507	21.3054	G
PGN0930														
		2.72 E-1	14.5	22.5	-0.6293	5.2112	Y	Y	9.57 E-3	4.48 E+6	7.28 E+6	-0.7004	23.4873	Y
PGN0933														
PG1189		2.04 E-3	142.9	90	0.6669	7.8635	R	R	5.53 E-6	1.12 E+8	6.89 E+7	0.7059	27.4347	R
PGN0935	*PG1059*	*hypothetical protein PG_1189*												
PG1190		3.85 E-1	5.1	2.5	1.0381	2.9324	Y	R	0	2.22 E+6	4.86 E+5	2.1899	21.3675	R
PGN0936	*PG1060*	*hprA glycerate dehydrogenase*												
PG1195		6.15 E-1	9.4	11	-0.2249	4.3514	Y	Y	1.52 E-1	2.47 E+6	2.3 E+6	0.1058	22.1867	Y
PGN0938	*PG1061*	*bioF-2 8-amino-7-oxononanoate synthase*												
		5.17 E-1	1.7	3	-0.8099	2.2361	Y	G	0	1.85 E+3	3.02 E+5	-7.3508	18.2133	G
PGN0945														
		4.15 E-1	2.6	5	-0.9619	2.9197	Y	Y	1.08 E-1	1.26 E+6	1.18 E+6	0.0969	21.2172	Y
PGN0946														
		6.52 E-2	6.0	16	-1.4175	4.4587	Y	G	8.04 E-4	1.79 E+6	4.39 E+6	-1.2910	22.5600	G
PGN0948														

TIGR	PGN	LANL	Description	PP PPC / Pg Spectral Count q-value	PP (Norm)	PPC (Norm)	log_2Ratio	log_2Sum	Regulation	PP PPC/ Pg Protein Intensity Regulation	q-value	PP (Norm)	PPC (Norm)	log_2Ratio	log_2Sum	33277 Consensus
	PGN0949			4.5 E-1	6.8	10	-0.5468	4.0743	Y	Y	3.81 E-2	3.35 E+6	2.56 E+6	0.3900	22.4939	Y
	PGN0950			5.43 E-1	3.4	5	-0.5468	3.0743	Y	Y	2.62 E-3	2.25 E+6	1.27 E+6	0.8280	21.7476	Y
	PGN0952			6.36 E-5	55.6	18.5	1.5880	6.2117	R	R	1.93 E-11	3.15 E+7	1.14 E+7	1.4730	25.3548	R
PG0992	PGN0962	PG0888	*thrS threonyl-tRNA synthetase*	0	201.1	60.5	1.7328	8.0311	R	R	0	7.67 E+7	2.86 E+7	1.4236	26.6491	R
PG0991	PGN0963	PG0887	*infC translation initiation factor IF-3*	2.68 E-4	18.0	1.5	3.5825	4.2831	R	R	0	2.4 E+7	2.28 E+6	3.3970	24.6467	R
PG0990	PGN0964		*rpmI ribosomal protein L35*	6.49 E-1	4.3	3.5	0.2897	2.9594	Y	R	2.56 E-4	4.08 E+6	5.58 E+5	2.8698	22.1441	R
PG0989	PGN0965	PG0886	*rplT ribosomal protein L20*	3.61 E-1	31.7	40.5	-0.3553	6.1731	Y	Y	3.5 E-2	2.49 E+7	1.88 E+7	0.4051	25.3812	Y
PG0982	PGN0972	PG0878	*TPR domain protein*	1.85 E-3	54.8	24	1.1901	6.2994	R	R	1.63 E-12	1.61 E+7	6.56 E+6	1.2962	24.4346	R
PG0980	PGN0973	PG0877	*hypothetical protein PG_0980*	6.69 E-1	2.6	3	-0.2249	2.4769	Y	Y	4.22 E-2	3.42 E+5	2.63 E+5	0.3774	19.2080	Y
PG0978	PGN0974	PG0875	*aroE shikimate 5-dehydrogenase*	6 E-1	1.7	2.5	-0.5468	2.0743	Y	Y	2.16 E-3	1.85 E+3	2.62 E+5	-7.1444	18.0083	Y
PG0977	PGN0975	PG0874	*ubiE ubiquinone/menaquinone biosynthesis methyltransferase UbiE*	2.92 E-1	3.4	7.5	-1.1318	3.4492	Y	Y	5.61 E-3	1.07 E+6	2.23 E+6	-1.0603	21.6516	Y
PG0976	PGN0976	PG0873	*phosphoribosylaminoimidazole-succinocarboxamide synthase putative*	1.85 E-3	65.0	31	1.0688	6.5854	R	R	6.02 E-4	3.99 E+7	2.41 E+7	0.7307	25.9314	R
PG0975	PGN0977	PG0872	*PhoH family protein*	4.88 E-1	8.6	6	0.5121	3.8636	Y	Y	1.83 E-2	1.23 E+6	5.75 E+5	1.0933	20.7820	Y
PG0973	PGN0980	PG0871	*alpha-12-mannosidase family protein*	4.23 E-1	2.6	1	1.3601	1.8347	Y	R	0	2.27 E+5	1 E+3	7.8236	17.7957	R
PG0968	PGN0982	PG0866	*mrr Mrr restriction system protein*	6.85 E-1	4.3	4.5	-0.0729	3.1339	Y	R	5.11 E-11	3.75 E+6	7.22 E+5	2.3753	22.0920	Y
PG0965	PGN0984	PG0865	*phosphatidylserine decarboxylase-related protein*	1.76 E-2	11.1	28	-1.3318	5.2900	Y	Y	1.69 E-3	3.13 E+6	8.99 E+6	-1.5225	23.5310	Y
PG0962	PGN0987	PG0861	*proS prolyl-tRNA synthetase*	5.47 E-4	52.2	20	1.3839	6.1738	R	R	5.03 E-5	2.38 E+7	1.08 E+7	1.1342	25.0439	R

TIGR		PP PPC / Pg Spectral Count						PP PPC/ Pg Protein Intensity						33277
PGN	*LANL*	*q*-value	PP (Norm)	PPC (Norm)	log_2Ratio	log_2Sum	Regulation		*q*-value	PP (Norm)	PPC (Norm)	log_2Ratio	log_2Sum	Consensus
PG0961		4.55 E-1	5.1	3	0.7751	3.0240	Y	Y	1.88 E-3	3.12 E+6	1.37 E+6	1.1821	22.0996	Y
PGN0988	*PG0860*	*hypothetical protein PG_0961*												
PG0960		3.05 E-1	3.4	1	1.7751	2.1449	Y	R	0	1.18 E+6	1 E+3	10.2070	20.1740	R
PGN0989	*PG0859*	*conserved hypothetical protein*												
PG0959		1.74 E-1	18.0	9.5	0.9195	4.7797	Y	Y	5.71 E-2	8.94 E+6	6.04 E+6	0.5651	23.8365	Y
PGN0990	*PG0858*	*mrp ATP-binding protein Mrp/Nbp35 family*												
PG0958		2.74 E-1	6.8	12.5	-0.8688	4.2739	Y	Y	2.25 E-2	1.21 E+6	2.82 E+6	-1.2263	21.9404	Y
PGN0991	*PG0857*	*ribonuclease BN putative*												
PG0957		3.05 E-1	2.6	6	-1.2249	3.0988	Y	Y	7.25 E-2	8.35 E+5	1.01 E+6	-0.2691	20.8130	Y
PGN0992	*PG0856*	*ribF riboflavin biosynthesis protein RibF*												
PG0956		3.05 E-1	3.4	1	1.7751	2.1449	Y	R	0	6.42 E+5	1 E+3	9.3272	19.2953	R
PGN0993	*PG0855*	*peptidase M23/M37 family putative*												
PG0955		2.93 E-1	6.8	3	1.1901	3.2994	Y	Y	8.01 E-3	2.52 E+6	1.1 E+6	1.1955	21.7892	Y
PGN0994	*PG0854*	*hypothetical protein PG_0955*												
PG0954		4.23 E-1	2.6	1	1.3601	1.8347	Y	R	0	1.7 E+6	1 E+3	10.7318	20.6984	R
PGN0996	*PG0853*	*TPR domain protein*												
PG0953		2.96 E-2	3.4	13.5	-1.9798	4.0809	Y	Y	4.96 E-3	6.72 E+5	1.07 E+7	-3.9871	23.4343	Y
PGN0997	*PG0852*	*dut deoxyuridine 5'-triphosphate nucleotidohydrolase*												
PG0952		2.97 E-2	16.3	34	-1.0644	5.6513	Y	Y	4.15 E-3	5.11 E+6	1.51 E+7	-1.5652	24.2696	Y
PGN0998	*PG0851*	*ispG 1-hydroxy-2-methyl-2-(E)-butenyl 4-diphosphate synthase*												
PG0951		4.4 E-1	18.0	13.5	0.4125	4.9758	Y	Y	5.2 E-2	1.24 E+7	9.09 E+6	0.4533	24.3596	Y
PGN0999	*PG0850*	*purE phosphoribosylaminoimidazole carboxylase PurE protein*												
PG0950		7.04 E-1	35.1	35	0.0034	6.1310	Y	Y	6.41 E-2	4.69 E+7	7.06 E+7	-0.5898	26.8086	Y
PGN1000	*PG0848*	*gcvH glycine cleavage system H protein*												
PG0949		4.4 E-1	1.7	3.5	-1.0323	2.3816	Y	G	4.51 E-6	1.85 E+3	4.57 E+5	-7.9485	18.8080	G
PGN1001	*PG0847*	*conserved hypothetical protein*												
PG0948		4.23 E-1	2.6	1	1.3601	1.8347	Y	R	0	6.44 E+5	1 E+3	9.3310	19.2990	R
PGN1002	*PG0846*	*AMP nucleosidase putative*												
PG0946		3.31 E-1	16.3	10.5	0.6307	4.7419	Y	R	4.42 E-4	1.19 E+7	6.43 E+6	0.8946	24.1314	R
PGN1004	*PG0844*	*ABC transporter ATP-binding protein*												
PG0945		5.71 E-1	18.0	21	-0.2249	5.2842	Y	Y	1.6 E-2	1.19 E+7	1.82 E+7	-0.6120	24.8448	Y
PGN1005	*PG0843*	*ABC transporter permease protein putative*												
PG0937		5.76 E-5	18.0	55	-1.6139	6.1892	G	Y	3.54 E-3	8.59 E+6	2.19 E+7	-1.3487	24.8612	G
PGN1010	*PG0836*	*hypothetical protein PG_0937*												

TIGR		PP PPC / Pg Spectral Count						PP PPC/ Pg Protein Intensity						33277
PGN	***LANL***	*q*-value	PP (Norm)	PPC (Norm)	log_2Ratio	log_2Sum	Regulation		*q*-value	PP (Norm)	PPC (Norm)	log_2Ratio	log_2Sum	Consensus
PG0936		3.06 E-1	4.3	1.5	1.5121	2.5306	Y	R	1.34 E-6	7.29 E+6	6.74 E+5	3.4350	22.9253	R
PGN1011	*PG0835*	*xanthine/uracil permease family protein*												
PG0935		2.25 E-1	1.7	5.5	-1.6843	2.8503	Y	Y	3.57 E-3	1.85 E+3	1.07 E+6	-9.1776	20.0338	Y
PGN1012	*PG0834*	*ispE 4-diphosphocytidyl-2C-methyl-D-erythritol kinase*												
PG0934		6.69 E-1	1.7	1.5	0.1901	1.6832	Y	G	0	1.85 E+3	2.12 E+5	-6.8385	17.7048	Y
PGN1013	*PG0833*	*radical SAM domain protein*												
PG0933		8.74 E-2	579.3	650.5	-0.1673	10.2642	Y	Y	9.55 E-3	6.3 E+8	5.42 E+8	0.2170	30.1257	Y
PGN1014	*PG0832*	*translation elongation factor G putative*												
PG0932		6.64 E-1	6.8	6	0.1901	3.6832	Y	R	5.08 E-5	2.51 E+6	1.05 E+6	1.2602	21.7620	R
PGN1015	*PG0831*	*DNA polymerase III delta prime subunit putative*												
		4.21 E-1	3.4	1.5	1.1901	2.2994	Y	R	0	1.25 E+6	1.1 E+5	3.4982	20.3727	R
PGN1018														
PG0928		6.96 E-1	33.4	33	0.0161	6.0525	Y	R	4.23 E-12	1.91 E+7	8.15 E+6	1.2263	24.6974	R
PGN1019	*PG0827*	*response regulator*												
PG0927		6.69 E-1	1.7	2	-0.2249	1.8919	Y	G	0	1.85 E+3	3.65 E+5	-7.6226	18.4837	G
PGN1020	*PG0826*	*conserved hypothetical protein TIGR00150*												
PG0925		4.4 E-1	1.7	3.5	-1.0323	2.3816	Y	G	2.01 E-9	6.33 E+4	5.62 E+5	-3.1502	19.2535	G
PGN1022	*PG0825*	*tmk thymidine kinase*												
PG0924		2.02 E-1	6.0	12.5	-1.0614	4.2086	Y	Y	4.4 E-3	9.59 E+5	2.76 E+6	-1.5247	21.8264	Y
PGN1023	*PG0824*	*5'-nucleotidase lipoprotein e(P4) family*												
PG0923		1.52 E-2	9.4	1	3.2345	3.3802	Y	R	0	3.75 E+6	1 E+3	11.8714	21.8375	R
PGN1024	*PG0823*	*rbfA ribosome-binding factor A*												
PG0920		1.62 E-1	7.7	2.5	1.6231	3.3506	Y	R	9.19 E-10	1.01 E+7	1.91 E+6	2.3952	23.5130	R
PGN1026	*PG0820*	*glycosyl transferase group 2 family protein*												
PG0919		5.67 E-4	9.4	34	-1.8529	5.4400	G	G	2.62 E-9	3.65 E+6	1.33 E+7	-1.8658	24.0136	G
PGN1027	*PG0819*	*pyrC dihydroorotase*												
PG0918		6 E-1	1.7	2.5	-0.5468	2.0743	Y	G	2.26 E-6	1.85 E+3	1.73 E+6	-9.8649	20.7202	G
PGN1028	*PG0818*	*hypothetical protein PG_0918*												
PG0914		1.49 E-1	12.0	22	-0.8770	5.0866	Y	G	8.18 E-4	4.47 E+6	9.4 E+6	-1.0730	23.7255	G
PGN1032	*PG0815*	*hypothetical protein PG_0914*												
PG0909		6.41 E-1	6.0	5	0.2605	3.4581	Y	Y	2.91 E-2	2.53 E+6	1.61 E+6	0.6478	21.9808	Y
PGN1035	*PG0811*	*conserved hypothetical protein*												
PG0906		8.7 E-2	40.2	24	0.7447	6.0049	Y	Y	1.62 E-1	1.01 E+8	1.11 E+8	-0.1363	27.6637	Y
PGN1037	*PG0809*	*lipoprotein putative*												

TIGR		PP PPC / Pg Spectral Count						PP PPC/ Pg Protein Intensity						33277
PGN	*LANL*	*q*-value	PP (Norm)	PPC (Norm)	log_2Ratio	log_2Sum	Regulation		*q*-value	PP (Norm)	PPC (Norm)	log_2Ratio	log_2Sum	Consensus
PG0903		1.84 E-1	1.7	6	-1.8099	2.9470	Y	G	3.46 E-8	1.85 E+3	4.19 E+6	-11.1447	21.9991	G
PGN1038	*PG0806*	*conserved hypothetical protein*												
PG0900		5.43 E-1	8.6	6.5	0.3966	3.9123	Y	Y	2.38 E-2	3.23 E+6	5.28 E+6	-0.7096	23.0200	Y
PGN1041	*PG0803*	*cydA cytochrome d ubiquinol oxidase subunit I*												
PG0898		6.02 E-1	5.1	4	0.3601	3.1912	Y	R	0	1.86 E+6	4.46 E+5	2.0585	21.1360	R
PGN1043	*PG0801*	*conserved hypothetical protein*												
PG0897		7.56 E-2	1.7	8	-2.2249	3.2797	Y	G	1.11 E-5	1.85 E+3	1.12 E+6	-9.2457	20.1018	G
PGN1044	*PG0800*	*alpha-amylase family protein*												
PG0890		1.76 E-2	78.7	117	-0.5717	7.6126	Y	G	4.89 E-7	4.45 E+7	9.07 E+7	-1.0267	27.0112	G
PGN1049	*PG0796*	*alkaline phosphatase putative*												
PG0889		5.14 E-1	16.3	20	-0.2989	5.1802	Y	Y	1.5 E-1	8.64 E+6	8.13 E+6	0.0868	23.9996	Y
PGN1050	*PG0795*	*peptidase M24 family*												
PG0886		6.69 E-1	1.7	1.5	0.1901	1.6832	Y	G	0	1.85 E+3	8.61 E+4	-5.5399	16.4243	Y
PGN1052	*PG0794*	*hypothetical protein PG_0886*												
PG0885		6.69 E-1	3.4	3	0.1901	2.6832	Y	Y	1.85 E-1	8 E+5	7.55 E+5	0.0836	20.5677	Y
PGN1053	*PG0793*	*phospho-2-dehydro-3-deoxyheptonate aldolase/chorismate mutase*												
PG0884		4.23 E-1	3.4	6	-0.8099	3.2361	Y	Y	1.91 E-1	9.95 E+5	9.77 E+5	0.0264	20.9115	Y
PGN1054	*PG0792*	*hypothetical protein PG_0884*												
PG0883		6 E-1	1.7	2.5	-0.5468	2.0743	Y	G	3.18 E-5	3.21 E+5	8.31 E+5	-1.3720	20.1353	G
PGN1055	*PG0791*	*hypothetical protein PG_0883*												
PG0882		5.17 E-1	1.7	3	-0.8099	2.2361	Y	G	2.39 E-4	1.85 E+3	6.08 E+5	-8.3605	19.2186	G
PGN1056	*PG0790*	*hypothetical protein PG_0882*												
PG0881		2.7 E-2	77.9	49	0.6682	6.9871	Y	Y	2.77 E-3	6.39 E+7	4.19 E+7	0.6106	26.6562	Y
PGN1057	*PG0789*	*recA recA protein*												
PG0880		5.94 E-2	11.1	3	1.8906	3.8200	Y	R	7.03 E-9	3.81 E+6	1.04 E+6	1.8686	22.2090	R
PGN1058	*PG0788*	*bcp bacterioferritin comigratory protein*												
PG0877		4.02 E-1	4.3	2	1.0970	2.6504	Y	R	1.22 E-5	2 E+6	1 E+6	0.9958	21.5197	R
PGN1061	*PG0787*	*hypothetical protein PG_0877*												
PG0876		2.52 E-1	3.4	8	-1.2249	3.5138	Y	Y	9.23 E-2	1.31 E+6	1.67 E+6	-0.3485	21.5051	Y
PGN1062	*PG0786*	*thdF thiophene and furan oxidation protein ThdF*												
		4.75 E-1	23.1	18.5	0.3205	5.3786	Y	Y	8.26 E-2	2.98 E+6	3.51 E+6	-0.2374	22.6300	Y
PGN1071														
		5.89 E-1	1.7	1	0.7751	1.4390	Y	R	0	1.29 E+6	1 E+3	10.3300	20.2969	R
PGN1072														

TIGR		PP PPC / Pg Spectral Count						PP PPC/ Pg Protein Intensity						33277
PGN	*LANL*	*q*-value	PP (Norm)	PPC (Norm)	log_2Ratio	log_2Sum	Regulation		*q*-value	PP (Norm)	PPC (Norm)	log_2Ratio	log_2Sum	Consensus
		5.17 E-1	3.4	2	0.7751	2.4390	Y	Y	6.07 E-2	1.67 E+6	9.2 E+5	0.8606	21.3053	Y
PGN1074														
PG1288		2.49 E-2	26.5	49	-0.8854	6.2389	Y	G	9.78 E-7	1.15 E+7	2.42 E+7	-1.0788	25.0883	G
PGN1078	*PG1134*	*gmd GDP-mannose 46-dehydratase*												
PG1289		3.57 E-3	1.7	14	-3.0323	3.9737	Y	G	2.05 E-7	5.5 E+5	3.45 E+6	-2.6495	21.9321	G
PGN1079	*PG1135*	*fcl GDP-fucose synthetase*												
PG1290		4.75 E-1	516.8	539.5	-0.0620	10.0448	Y	Y	2.15 E-2	3.43 E+8	4.73 E+8	-0.4652	29.6044	Y
PGN1080	*PG1136*	*ilvE branched-chain amino acid aminotransferase*												
PG1291		1.3 E-3	21.4	4	2.4190	4.6663	R	R	1.04 E-12	8.14 E+6	9.67 E+5	3.0730	23.1188	R
PGN1081	*PG1137*	*hypothetical protein PG_1291*												
PG1294		3.5 E-1	4.3	8	-0.9030	3.6180	Y	R	5.04 E-4	1.52 E+6	7.22 E+5	1.0755	21.0966	Y
PGN1085	*PG1138*	*feoB-2 ferrous iron transport protein B*												
PG1297		0	350.8	117.5	1.5781	8.8713	R	R	2.26 E-10	3.27 E+8	1.5 E+8	1.1240	28.8315	R
PGN1088	*PG1140*	*rpsA ribosomal protein S1*												
PG1300		6.69 E-1	1.7	1.5	0.1901	1.6832	Y	G	0	1.85 E+3	9.21 E+4	-5.6372	16.5196	Y
PGN1089	*PG1141*	*conserved hypothetical protein*												
PG1301		5.43 E-1	2.6	1.5	0.7751	2.0240	Y	R	0	8.98 E+5	3.06 E+5	1.5543	20.1984	R
PGN1090	*PG1142*	*hypothetical protein PG_1301*												
PG1302		8.78 E-3	73.6	41.5	0.8263	6.8466	R	Y	8.7 E-2	7.57 E+7	6.12 E+7	0.3066	27.0293	R
PGN1091	*PG1143*	*hypothetical protein PG_1302*												
PG1304		4.4 E-1	1.7	3.5	-1.0323	2.3816	Y	Y	5.41 E-2	8.81 E+5	1.89 E+6	-1.0994	21.4010	Y
PGN1093	*PG1145*	*hypothetical protein PG_1304*												
PG1305		2.13 E-1	426.1	471	-0.1445	9.8091	Y	G	1.2 E-5	3.84 E+8	5.51 E+8	-0.5220	29.7992	G
PGN1094	*PG1146*	*gcvP glycine cleavage system P protein*												
PG1306		3.85 E-1	5.1	2.5	1.0381	2.9324	Y	R	6.7 E-6	3.58 E+6	1.06 E+6	1.7588	22.1431	R
PGN1095	*PG1147*	*metallo-beta-lactamase family protein*												
PG1307		1.32 E-1	7.7	16.5	-1.0994	4.5970	Y	Y	1.42 E-1	2.43 E+6	2.09 E+6	0.2192	22.1090	Y
PGN1096	*PG1148*	*gidB glucose-inhibited division protein B*												
PG1308		2.74 E-1	10.3	17	-0.7274	4.7691	Y	G	5.03 E-5	2.24 E+6	5.9 E+6	-1.3945	22.9564	G
PGN1097	*PG1149*	*hypothetical protein PG_1308*												
PG1310		2 E-1	4.3	10	-1.2249	3.8357	Y	Y	1.12 E-1	1.29 E+6	1.48 E+6	-0.1998	21.4041	Y
PGN1098	*PG1150*	*exsB protein*												
PG1311		2.22 E-2	1.7	10.5	-2.6172	3.6101	Y	G	6.39 E-8	2.85 E+5	1.2 E+6	-2.0783	20.5063	G
PGN1099	*PG1151*	*conserved hypothetical protein*												

TIGR		PP PPC / Pg Spectral Count							PP PPC/ Pg Protein Intensity					33277
PGN	*LANL*	*q*-value	PP (Norm)	PPC (Norm)	log_2Ratio	log_2Sum	Regulation		*q*-value	PP (Norm)	PPC (Norm)	log_2Ratio	log_2Sum	Consensus
PG1312		3.05 E-1	3.4	1	1.7751	2.1449	Y	R	0	9.18 E+5	1 E+3	9.8416	19.8090	R
PGN1100	*PG1152*	*capA protein putative*												
PG1313		5.73 E-5	110.4	54	1.0314	7.3609	R	R	5.24 E-11	8.82 E+7	4.23 E+7	1.0606	26.9594	R
PGN1103	*PG1153*	*dipeptidase-related protein*												
PG1315		6.56 E-1	33.4	35.5	-0.0892	6.1058	Y	Y	1.71 E-1	2.64 E+7	2.78 E+7	-0.0751	25.6905	Y
PGN1105	*PG1155*	*slyD peptidyl-prolyl cis-trans isomerase SlyD FKBP-type*												
PG1321		1.85 E-1	185.7	217.5	-0.2282	8.6553	Y	Y	1.17 E-3	6.51 E+7	1.02 E+8	-0.6491	27.3166	Y
PGN1111	*PG1160*	*fhs formate--tetrahydrofolate ligase*												
PG1323		2.18 E-7	40.2	5	3.0078	5.4988	R	R	0	2.18 E+7	2.58 E+6	3.0811	24.5422	R
PGN1112	*PG1161*	*PhoH family protein*												
PG1324		6.69 E-1	1.7	2	-0.2249	1.8919	Y	G	5.04 E-6	1.85 E+3	5.38 E+5	-8.1826	19.0413	G
PGN1113	*PG1162*	*ruvC crossover junction endodeoxyribonuclease RuvC*												
PG1325		6.69 E-1	2.6	3	-0.2249	2.4769	Y	Y	5.31 E-2	3.18 E+5	1.12 E+6	-1.8196	20.4582	Y
PGN1114	*PG1163*	*hypothetical protein PG_1325*												
PG1326		8.79 E-2	6.8	16.5	-1.2693	4.5451	Y	Y	1.67 E-3	2.97 E+6	6.31 E+6	-1.0867	23.1469	Y
PGN1115	*PG1164*	*hemagglutinin putative*												
PG1327		3.49 E-1	220.8	244	-0.1444	8.8603	Y	G	3.09 E-6	1.56 E+8	2.76 E+8	-0.8260	28.6874	G
PGN1116	*PG1165*	*aminotransferase putative*												
PG1328		3.06 E-9	146.3	57.5	1.3475	7.6711	R	R	0	1.36 E+8	5.13 E+7	1.4108	27.4836	R
PGN1117	*PG1166*	*CoA ligase family protein*												
PG1330		1.04 E-4	5.1	29	-2.4979	5.0931	G	G	4.27 E-5	2.87 E+6	1.26 E+7	-2.1276	23.8790	G
PGN1119	*PG1167*	*mscL large conductance mechanosensitive channel protein*												
		2.77 E-3	722.2	599	0.2698	10.3676	R	Y	5.43 E-2	9.62 E+8	8.59 E+8	0.1636	30.7617	R
PGN1120														
PG1332		5.2 E-1	20.5	24.5	-0.2547	5.4930	Y	Y	7.14 E-3	1.94 E+7	4.07 E+7	-1.0657	25.8420	Y
PGN1122	*PG1170*	*pntB NAD(P) transhydrogenase beta subunit*												
PG1333		9.48 E-2	1.7	7.5	-2.1318	3.2034	Y	G	3.65 E-10	1.85 E+3	1.33 E+6	-9.4908	20.3465	G
PGN1123	*PG1171*	*hypothetical protein PG_1333*												
PG1334		1.47 E-2	63.3	35.5	0.8348	6.6267	Y	R	5.29 E-7	3.86 E+7	1.96 E+7	0.9766	25.7936	R
PGN1124	*PG1172*	*band 7/Mec-2 family protein*												
PG1335		1.6 E-1	11.1	20.5	-0.8820	4.9829	Y	Y	1.38 E-2	7.71 E+6	1.47 E+7	-0.9343	24.4190	Y
PGN1125	*PG1173*	*membrane protein putative*												
PG1340		3.82 E-1	1.7	4	-1.2249	2.5138	Y	Y	3.09 E-3	1.85 E+3	8.35 E+5	-8.8175	19.6744	Y
PGN1128	*PG1176*	*L-lactate permease*												

TIGR		PP PPC / Pg Spectral Count							PP PPC/ Pg Protein Intensity						33277
PGN	*LANL*	*q*-value	PP (Norm)	PPC (Norm)	log_2Ratio	log_2Sum	Regulation		*q*-value	PP (Norm)	PPC (Norm)	log_2Ratio	log_2Sum		Consensus
PG1341		1.32 E-10	109.5	31	1.8209	7.1347	R	R	4.37 E-15	2.21 E+8	5.72 E+7	1.9481	28.0508		R
PGN1129	*PG1177*	*hypothetical protein PG_1341*													
PG1342		5.43 E-1	2.6	1.5	0.7751	2.0240	Y	R	0	1.03 E+6	2.18 E+5	2.2357	20.2453		R
PGN1130	*PG1178*	*murB UDP-N-acetylenolpyruvoylglucosamine reductase*													
PG1343		1.5 E-1	1.7	6.5	-1.9253	3.0376	Y	G	6.34 E-7	1.85 E+3	7.6 E+5	-8.6812	19.5384		G
PGN1131	*PG1179*	*lipB lipoate-protein ligase B*													
PG1345		7.79 E-2	6.0	15.5	-1.3717	4.4256	Y	Y	2.15 E-2	1.07 E+6	3.06 E+6	-1.5140	21.9772		Y
PGN1134	*PG1180*	*glycosyl transferase group 1 family protein*													
PG1346		6.7 E-1	5.1	5.5	-0.0994	3.4106	Y	Y	1.49 E-2	1.24 E+6	7.45 E+5	0.7384	20.9228		Y
PGN1135	*PG1181*	*glycosyl transferase group 1 family protein*													
PG1347		5.77 E-2	5.1	15	-1.5468	4.3316	Y	G	3.6 E-4	2.13 E+6	6.87 E+6	-1.6882	23.1016		G
PGN1136	*PG1182*	*conserved hypothetical protein*													
PG1348		1.56 E-1	6.0	1.5	1.9975	2.9049	Y	Y	5.04 E-2	2.14 E+6	2.51 E+6	-0.2343	22.1478		Y
PGN1137	*PG1183*	*conserved hypothetical protein TIGR00147*													
PG1351		6 E-1	1.7	2.5	-0.5468	2.0743	Y	G	2.33 E-4	1.85 E+3	1.4 E+6	-9.5583	20.4139		G
PGN1138	*PG1186*	*hypothetical protein PG_1351*													
PG1352		4.1 E-2	21.4	8.5	1.3315	4.9017	Y	R	8.92 E-4	1.35 E+7	5.97 E+6	1.1790	24.2162		R
PGN1139	*PG1187*	*hypothetical protein PG_1352*													
PG1353		3.11 E-2	65.0	39.5	0.7192	6.7078	Y	R	2.33 E-4	4.19 E+7	2.41 E+7	0.8012	25.9757		R
PGN1140	*PG1188*	*pyrE orotate phosphoribosyltransferase*													
PG1354		2.25 E-1	1.7	5.5	-1.6843	2.8503	Y	Y	8.62 E-3	1.85 E+3	1.06 E+6	-9.1576	20.0138		Y
PGN1141	*PG1189*	*hydrolase carbon-nitrogen family*													
PG1356		2.99 E-1	38.5	50	-0.3769	6.4677	Y	Y	3.49 E-3	3 E+7	1.71 E+7	0.8125	25.4882		Y
PGN1143	*PG1191*	*hypothetical protein PG_1356*													
PG1360		6 E-1	62.5	67	-0.1012	7.0164	Y	Y	1.11 E-2	3.94 E+7	5.05 E+7	-0.3593	26.4222		Y
PGN1148	*PG1195*	*purD phosphoribosylamine--glycine ligase*													
PG1361		3.21 E-1	111.2	129	-0.2138	7.9083	Y	Y	2.21 E-3	6.9 E+7	9.21 E+7	-0.4154	27.2637		Y
PGN1149	*PG1196*	*dipeptidyl aminopeptidase IV putative*													
PG1362		6.69 E-1	6.8	7.5	-0.1318	3.8425	Y	R	2.6 E-7	4.57 E+6	9.76 E+5	2.2270	22.4031		Y
PGN1150	*PG1197*	*conserved hypothetical protein*													
PG1364		4.4 E-1	5.1	8	-0.6399	3.7152	Y	Y	1.15 E-1	1.39 E+6	1.76 E+6	-0.3372	21.5856		Y
PGN1151	*PG1199*	*dxr 1-deoxy-D-xylulose 5-phosphate reductoisomerase*													
PG1365		3.21 E-1	1.7	4.5	-1.3948	2.6349	Y	G	1.22 E-6	1.85 E+3	6.23 E+5	-8.3956	19.2536		G
PGN1152	*PG1200*	*16S rRNA processing protein RimM putative*													

TIGR		PP PPC / Pg Spectral Count							PP PPC/ Pg Protein Intensity					33277
PGN	*LANL*	*q*-value	PP (Norm)	PPC (Norm)	log_2Ratio	log_2Sum	Regulation		*q*-value	PP (Norm)	PPC (Norm)	log_2Ratio	log_2Sum	Consensus
PG1366		4.41 E-1	34.2	28	0.2897	5.9594	Y	Y	1.66 E-1	1.93 E+7	2.01 E+7	-0.0608	25.2293	Y
PGN1153	*PG1201*	*murA UDP-N-acetylglucosamine 1-carboxyvinyltransferase*												
PG1367		2.27 E-2	18.0	5.5	1.7080	4.5527	Y	R	2.66 E-6	6.74 E+6	1.1 E+6	2.6099	22.9034	R
PGN1154	*PG1202*	*hypothetical protein PG_1367*												
PG1368		6.54 E-1	34.2	36.5	-0.0928	6.1442	Y	Y	2.82 E-3	9.17 E+6	1.32 E+7	-0.5255	24.4148	Y
PGN1155	*PG1203*	*pgi glucose-6-phosphate isomerase*												
PG1369		3.76 E-1	6.0	3	0.9975	3.1683	Y	G	0	1.86 E+6	3.18 E+6	-0.7727	22.2636	Y
PGN1156	*PG1204*	*gpsA glycerol-3-phosphate dehydrogenase (NAD(P)+)*												
PG1370		1.88 E-1	54.8	72.5	-0.4048	6.9917	Y	G	5.47 E-4	2.92 E+7	5.15 E+7	-0.8196	26.2649	G
PGN1157	*PG1205*	*lysS lysyl-tRNA synthetase*												
PG1371		7.24 E-2	47.1	70	-0.5728	6.8711	Y	Y	1.48 E-1	1.71 E+7	1.82 E+7	-0.0900	25.0748	Y
PGN1158	*PG1206*	*phosphorylase family protein*												
PG1372		2.67 E-1	9.4	4.5	1.0646	3.7983	Y	R	1.67 E-4	6.14 E+6	2.13 E+6	1.5305	22.9797	R
PGN1159	*PG1207*	*hypothetical protein PG_1372*												
PG1066		5.36 E-1	24.8	21	0.2408	5.5177	Y	Y	9.62 E-2	3.47 E+7	4.06 E+7	-0.2267	26.1644	Y
PGN1162	*PG0948*	*ctfA butyrate-acetoacetate CoA-transferase subunit A*												
PG1067		3.99 E-1	20.5	15	0.4532	5.1512	Y	Y	1.6 E-1	1.52 E+7	1.42 E+7	0.0997	24.8099	Y
PGN1163	*PG0949*	*conserved hypothetical protein*												
PG1068		3.82 E-1	80.4	93.5	-0.2172	7.4424	Y	G	3.58 E-5	3.88 E+7	7.33 E+7	-0.9195	26.7405	G
PGN1164	*PG0950*	*conserved hypothetical protein*												
PG1069		2.18 E-7	111.2	43	1.3712	7.2690	R	R	1.11 E-12	7.63 E+7	2.82 E+7	1.4337	26.6393	R
PGN1165	*PG0951*	*alcohol dehydrogenase zinc-containing putative*												
PG1070		6.69 E-1	33.4	35	-0.0688	6.0953	Y	Y	1.13 E-1	1.31 E+7	1.73 E+7	-0.4076	24.8572	Y
PGN1166	*PG0952*	*kamA L-lysine 23-aminomutase*												
PG1071		6.69 E-1	1.7	1.5	0.1901	1.6832	Y	G	0	4.51 E+5	5.38 E+5	-0.2553	19.9161	Y
PGN1167	*PG0953*	*conserved hypothetical protein*												
PG1072		6.69 E-1	1.7	1.5	0.1901	1.6832	Y	G	0	1.85 E+3	1.1 E+5	-5.8885	16.7663	Y
PGN1168	*PG0954*	*MutS family protein*												
PG1073		5.77 E-2	8.6	20.5	-1.2605	4.8608	Y	Y	3.32 E-3	5.91 E+6	9.76 E+6	-0.7246	23.9019	Y
PGN1169	*PG0955*	*kamD D-lysine 56-aminomutase alpha subunit*												
PG1074		6.64 E-1	13.7	12.5	0.1312	4.7110	Y	Y	5.2 E-2	5.22 E+6	7.11 E+6	-0.4448	23.5562	Y
PGN1170	*PG0956*	*kamE D-lysine 56-aminomutase beta subunit*												
PG1075		3.93 E-1	10.3	15	-0.5468	4.6592	Y	Y	3.48 E-3	6.92 E+6	1.17 E+7	-0.7557	24.1498	Y
PGN1171	*PG0957*	*coenzyme A transferase beta subunit*												

TIGR		PP PPC / Pg Spectral Count						PP PPC/ Pg Protein Intensity						33277
PGN	*LANL*	*q*-value	PP (Norm)	PPC (Norm)	$\log_2$Ratio	$\log_2$Sum	Regulation		*q*-value	PP (Norm)	PPC (Norm)	$\log_2$Ratio	$\log_2$Sum	Consensus
PG1076		3.14 E-4	397.9	519	-0.3834	9.8406	G	Y	4.32 E-3	2.66 E+8	3.44 E+8	-0.3717	29.1853	G
PGN1172	*PG0958*	*acdA acyl-CoA dehydrogenase short-chain specific*												
PG1077		1.72 E-2	338.0	413.5	-0.2909	9.5536	Y	Y	5.28 E-2	3.16 E+8	3.78 E+8	-0.2591	29.3706	Y
PGN1173	*PG0959*	*etfB-2 electron transfer flavoprotein beta subunit*												
PG1078		6.74 E-1	611.8	615.5	-0.0087	10.2613	Y	Y	3.13 E-2	3.45 E+8	3.13 E+8	0.1433	29.2933	Y
PGN1174	*PG0960*	*etfA-2 electron transfer flavoprotein alpha subunit*												
PG1079		4.02 E-1	24.0	18	0.4125	5.3909	Y	Y	1.62 E-1	1.7 E+7	1.82 E+7	-0.0919	25.0691	Y
PGN1175	*PG0961*	*enoyl-CoA hydratase/isomerase family protein*												
PG1080		4.82 E-1	46.2	53	-0.1979	6.6323	Y	Y	2.1 E-2	3.14 E+7	4.96 E+7	-0.6595	26.2719	Y
PGN1176	*PG0962*	*3-hydroxyacyl-CoA dehydrogenase family protein*												
PG1081		2.7 E-1	99.3	81.5	0.2843	7.4979	Y	Y	1.46 E-1	6.37 E+7	6.02 E+7	0.0827	26.8843	Y
PGN1178	*PG0963*	*ackA acetate kinase*												
PG1082		0	274.7	62	2.1473	8.3952	R	R	5.28 E-12	2.24 E+8	8.88 E+7	1.3337	28.2202	R
PGN1179	*PG0964*	*pta phosphotransacetylase*												
PG1083		6.69 E-1	1.7	2	-0.2249	1.8919	Y	G	0	1.85 E+3	2.26 E+6	-10.2524	21.1072	G
PGN1180	*PG0965*	*hypothetical protein PG_1083*												
PG1084		3.36 E-2	251.6	199	0.3381	8.8156	Y	R	3.22 E-15	3.07 E+8	1.4 E+8	1.1329	28.7368	R
PGN1181	*PG0966*	*thioredoxin family protein*												
PG1085		1.18 E-9	70.2	13.5	2.3778	6.3865	R	R	3.48 E-4	1.78 E+8	7.21 E+7	1.3031	27.8982	R
PGN1182	*PG0967*	*hypothetical protein PG_1085*												
PG1087		6.69 E-1	3.4	4	-0.2249	2.8919	Y	Y	1.87 E-1	7.01 E+5	7.16 E+5	-0.0302	20.4346	Y
PGN1184	*PG0968*	*radical SAM protein TIGR01212 family*												
PG1089		9.91 E-3	61.6	33	0.9006	6.5639	R	R	5.67 E-4	3.62 E+7	2.21 E+7	0.7135	25.7961	R
PGN1186	*PG0970*	*rprY DNA-binding response regulator RprY*												
PG1091		2.74 E-1	15.4	9	0.7751	4.6089	Y	Y	2.95 E-2	2.64 E+6	4.12 E+6	-0.6404	22.6897	Y
PGN1187	*PG0971*	*DHH subfamily 1 protein*												
PG1093		5.46 E-4	8.6	32.5	-1.9253	5.3595	G	G	2.22 E-5	6.73 E+6	2.11 E+7	-1.6508	24.7311	G
PGN1188	*PG0972*	*hypothetical protein PG_1093*												
PG1094		1.62 E-1	65.0	85.5	-0.3948	7.2339	Y	Y	1.98 E-2	4.29 E+7	5.78 E+7	-0.4291	26.5864	Y
PGN1189	*PG0973*	*pgm phosphomannomutase*												
PG1095		3.64 E-1	14.5	9.5	0.6146	4.5877	Y	Y	4.25 E-3	3.15 E+6	1.75 E+6	0.8447	22.2239	Y
PGN1190	*PG0974*	*RNA methyltransferase TrmA family*												
PG1096		6.49 E-1	4.3	3.5	0.2897	2.9594	Y	Y	1.4 E-1	6.49 E+5	8.48 E+5	-0.3846	20.5140	Y
PGN1193	*PG0975*	*hypothetical protein PG_1096*												

TIGR		PP PPC / Pg Spectral Count							PP PPC/ Pg Protein Intensity					33277
PGN	*LANL*	*q*-value	PP (Norm)	PPC (Norm)	log_2Ratio	log_2Sum	Regulation		*q*-value	PP (Norm)	PPC (Norm)	log_2Ratio	log_2Sum	Consensus
PG1097		4.4 E-1	5.1	8	-0.6399	3.7152	Y	Y	4.36 E-2	9.17 E+5	1.45 E+6	-0.6611	21.1750	Y
PGN1194	*PG0976*	*Mur ligase domain protein/alanine racemase*												
PG1098		4.65 E-1	2.6	4.5	-0.8099	2.8211	Y	Y	1.32 E-3	2.3 E+5	1.88 E+5	0.2896	18.6758	Y
PGN1195	*PG0977*	*hypothetical protein PG_1098*												
PG1100		6.69 E-1	1.7	2	-0.2249	1.8919	Y	G	0	1.85 E+3	1.06 E+6	-9.1593	20.0156	G
PGN1197	*PG0979*	*hypothetical protein PG_1100*												
PG1497		6.69 E-1	3.4	4	-0.2249	2.8919	Y	Y	5.33 E-2	5.54 E+5	4.67 E+5	0.2458	19.9622	Y
PGN1199	*PG1312*	*DNA-binding protein histone-like family*												
PG1103		6.64 E-1	6.8	6	0.1901	3.6832	Y	Y	1.5 E-1	2.55 E+6	2.25 E+6	0.1824	22.1952	Y
PGN1200	*PG0982*	*ATPase AAA family*												
PG1105		4.4 E-1	1.7	3.5	-1.0323	2.3816	Y	Y	6.1 E-3	1.85 E+3	7.49 E+5	-8.6617	19.5190	Y
PGN1202	*PG0984*	*rpoN RNA polymerase sigma-54 factor*												
PG1106		1.16 E-2	12.8	32	-1.3180	5.4865	Y	Y	2.64 E-2	4.09 E+6	7.88 E+6	-0.9459	23.5134	Y
PGN1203	*PG0985*	*murF UDP-N-acetylmuramoylalanyl-D-glutamyl-26-diaminopimelate--D-alanyl-D-alanyl ligase*												
PG1114		4.67 E-3	1.7	13.5	-2.9798	3.9271	Y	Y	1.24 E-3	8.71 E+5	1.08 E+7	-3.6359	23.4799	Y
PGN1204	*PG0995*	*panD aspartate-1-decarboxylase*												
PG1115		6.16 E-4	41.9	14	1.5825	5.8055	R	R	4.14 E-15	1.84 E+7	8.32 E+6	1.1470	24.6724	R
PGN1205	*PG0996*	*ffh signal recognition particle protein*												
PG1116		2.35 E-10	118.1	36.5	1.6938	7.2722	R	R	0	8.31 E+7	2.37 E+7	1.8102	26.6707	R
PGN1206	*PG0997*	*folD methylenetetrahydrofolate dehydrogenase/methenyltetrahydrofolate cyclohydrolase*												
PG1118		3.96 E-7	91.6	32	1.5166	6.9490	R	R	0	3.76 E+7	9.97 E+6	1.9155	25.5034	R
PGN1208	*PG1000*	*clpB clpB protein*												
PG1119		3.79 E-4	30.8	70	-1.1843	6.6554	G	G	4.4 E-5	1.4 E+7	4.18 E+7	-1.5732	25.7345	G
PGN1209	*PG1001*	*flavodoxin putative*												
		6.54 E-1	2.6	2	0.3601	2.1912	Y	Y	8.41 E-2	7.54 E+5	5.45 E+5	0.4671	20.3091	Y
PGN1211														
		3.06 E-1	4.3	1.5	1.5121	2.5306	Y	R	2.58 E-16	5.46 E+5	3.96 E+5	0.4612	19.8456	R
PGN1212														
		6.69 E-1	8.6	8	0.0970	4.0493	Y	Y	1.23 E-2	4.12 E+6	2.51 E+6	0.7165	22.6608	Y
PGN1213														
		2.74 E-1	1.7	5	-1.5468	2.7466	Y	Y	2.48 E-3	1.85 E+3	9.1 E+5	-8.9420	19.7986	Y
PGN1214														
PG1121		1.76 E-2	165.1	119	0.4727	8.1505	Y	Y	2.89 E-3	1.17 E+8	8.29 E+7	0.4924	27.5718	Y
PGN1218	*PG1002*	*asnS asparaginyl-tRNA synthetase*												

TIGR / PGN	LANL	PP PPC / Pg Spectral Count q-value	PP (Norm)	PPC (Norm)	log_2Ratio	log_2Sum	Regulation		PP PPC/ Pg Protein Intensity q-value	PP (Norm)	PPC (Norm)	log_2Ratio	log_2Sum	33277 Consensus
PG1122		6.02 E-5	48.8	14.5	1.7500	5.9835	R	R	0	2.4 E+7	3.83 E+6	2.6481	24.7324	R
PGN1219	*PG1003*													
PG1123		6.85 E-1	98.4	99.5	-0.0160	7.6286	Y	Y	1.76 E-2	4.94 E+7	6.33 E+7	-0.3562	26.7484	Y
PGN1220	*PG1004*	*purB adenylosuccinate lyase*												
PG1124		2.15 E-1	13.7	7	0.9677	4.3709	Y	Y	6.66 E-2	2.05 E+6	1.51 E+6	0.4482	21.7638	Y
PGN1221	*PG1005*	*ATP:cob(I)alamin adenosyltransferase putative*												
PG1125		3.05 E-1	3.4	1	1.7751	2.1449	Y	R	0	2.72 E+5	1 E+3	8.0880	18.0591	R
PGN1222	*PG1006*	*hypothetical protein PG_1125*												
PG1126		4.75 E-1	3.4	5.5	-0.6843	3.1575	Y	Y	5.14 E-2	1.67 E+6	5.12 E+6	-1.6201	22.6948	Y
PGN1223	*PG1007*	*uraA uracil permease*												
PG1127		2.15 E-3	12.8	1	3.6820	3.7902	Y	R	0	5.27 E+6	1 E+3	12.3644	22.3305	R
PGN1224	*PG1008*	*transcriptional regulator AsnC Family*												
PG1128		5.17 E-1	1.7	3	-0.8099	2.2361	Y	Y	1.29 E-3	1.85 E+3	4.27 E+5	-7.8515	18.7115	Y
PGN1225	*PG1009*	*xseA exodeoxyribonuclease VII large subunit*												
PG1129		0	239.6	46	2.3808	8.1578	R	R	0	9.05 E+7	1.88 E+7	2.2654	26.7041	R
PGN1226	*PG1010*	*nrd ribonucleotide reductase*												
PG1130		4.23 E-1	9.4	6	0.6496	3.9460	Y	Y	2.51 E-2	1.98 E+6	1.21 E+6	0.7077	21.6030	Y
PGN1227	*PG1011*	*TPR domain protein*												
PG1132		9.4 E-6	99.3	42.5	1.2237	7.1473	R	R	0	7.12 E+7	2.26 E+7	1.6547	26.4842	R
PGN1229	*PG1013*	*valS valyl-tRNA synthetase*												
PG1133		3.87 E-2	7.7	1	2.9450	3.1212	Y	R	0	1.96 E+6	1 E+3	10.9386	20.9051	R
PGN1230	*PG1014*	*hypothetical protein PG_1133*												
PG1134		1.27 E-2	35.9	64.5	-0.8438	6.6502	Y	Y	5.9 E-2	3.85 E+7	5.29 E+7	-0.4606	26.4456	Y
PGN1232	*PG1016*	*trxB thioredoxin reductase*												
PG1135		2.55 E-1	10.3	5	1.0381	3.9324	Y	Y	1.5 E-1	2.94 E+6	2.44 E+6	0.2681	22.3592	Y
PGN1233	*PG1017*	*bacterial sugar transferase*												
PG1137		6.69 E-1	1.7	1.5	0.1901	1.6832	Y	G	0	1.85 E+3	4.92 E+5	-8.0541	18.9133	Y
PGN1235	*PG1019*	*porS porS protein*												
PG1138		6 E-1	3.4	4.5	-0.3948	2.9860	Y	Y	4.74 E-2	5.85 E+5	1.15 E+6	-0.9710	20.7232	Y
PGN1236	*PG1020*	*porR pigmentation and extracellular proteinase regulator*												
PG1139		8.55 E-2	11.1	3.5	1.6682	3.8702	Y	Y	6.61 E-2	2 E+6	2.95 E+6	-0.5584	22.2380	Y
PGN1238	*PG1021*	*hypothetical protein PG_1139*												
PG1140		6.69 E-1	3.4	3	0.1901	2.6832	Y	R	1.93 E-4	8.72 E+5	4.96 E+5	0.8130	20.3832	R
PGN1239	*PG1022*	*glycosyl transferase group 2 family protein*												

TIGR		PP PPC / Pg Spectral Count						PP PPC/ Pg Protein Intensity						33277
PGN	***LANL***	*q*-value	PP (Norm)	PPC (Norm)	log_2Ratio	log_2Sum	Regulation		*q*-value	PP (Norm)	PPC (Norm)	log_2Ratio	log_2Sum	Consensus
PG1141		4.45 E-2	18.0	6.5	1.4670	4.6129	Y	Y	6.43 E-3	3.48 E+6	1.91 E+6	0.8692	22.3618	Y
PGN1240	*PG1023*	*glycosyl transferase group 1 family protein*												
PG1143		6.77 E-1	4.3	4	0.0970	3.0493	Y	Y	3.21 E-3	1.56 E+6	1.23 E+6	0.3455	21.4144	Y
PGN1243	*PG1026*	*sugar dehydrogenase UDP-glucose/GDP-mannose dehydrogenase family*												
PG1144		2.52 E-1	5.1	10.5	-1.0323	3.9666	Y	Y	7 E-2	1.33 E+6	2.09 E+6	-0.6498	21.7047	Y
PGN1244	*PG1027*													
PG1145		6.94 E-1	10.3	10.5	-0.0323	4.3763	Y	Y	6.5 E-3	4.19 E+6	6.92 E+6	-0.7220	23.4057	Y
PGN1245	*PG1028*	*long-chain-fatty-acid--CoA ligase putative*												
		4.8 E-1	4.3	2.5	0.7751	2.7609	Y	R	3.27 E-13	2.67 E+6	1.2 E+5	4.4767	21.4143	R
PGN1246														
PG1149		6.7 E-1	5.1	5.5	-0.0994	3.4106	Y	R	0	2.45 E+6	6.26 E+5	1.9708	21.5540	Y
PGN1251	*PG1029*	*glycosyl transferase group 1 family protein*												
PG1151		3.24 E-3	35.1	12.5	1.4888	5.5723	R	R	1.01 E-15	1.74 E+7	4.5 E+6	1.9540	24.3877	R
PGN1252	*PG1031*	*alcohol dehydrogenase iron-containing*												
		1.41 E-1	69.3	50	0.4711	6.8985	Y	Y	5.05 E-3	2.75 E+7	4.87 E+7	-0.8234	26.1844	Y
PGN1254														
PG1155		6 E-1	1.7	2.5	-0.5468	2.0743	Y	Y	9.35 E-3	1.85 E+3	9.13 E+5	-8.9458	19.8024	Y
PGN1255	*PG1034*	*ADP-heptose--LPS heptosyltransferase putative*												
PG1156		6.69 E-1	1.7	2	-0.2249	1.8919	Y	Y	6.01 E-3	1.85 E+3	1.43 E+6	-9.5918	20.4473	Y
PGN1256	*PG1035*	*S4 domain protein*												
PG1160		6.54 E-1	2.6	2	0.3601	2.1912	Y	Y	9.55 E-3	6.76 E+4	7.16 E+4	-0.0840	17.0865	Y
PGN1259	*PG1037*	*L-threonine-O-3-phosphate decarboxylase putative*												
PG1163		2.67 E-1	2.6	6.5	-1.3404	3.1806	Y	Y	1.14 E-2	1.45 E+6	9.85 E+5	0.5601	21.2161	Y
PGN1262	*PG1040*	*cbiA cobyrinic acid ac-diamide synthase*												
PG1164		3.82 E-1	19.7	14	0.4913	5.0738	Y	Y	1.98 E-1	4.18 E+6	4.21 E+6	-0.0103	23.0010	Y
PGN1263	*PG1041*	*hypothetical protein PG_1164*												
PG1165		6.69 E-1	1.7	2	-0.2249	1.8919	Y	G	0	1.85 E+3	1.56 E+5	-6.3995	17.2702	G
PGN1264	*PG1042*	*hypothetical protein PG_1165*												
PG1171		4.89 E-1	35.9	30.5	0.2367	6.0539	Y	Y	6.11 E-3	1.66 E+7	9.96 E+6	0.7414	24.6649	Y
PGN1268	*PG1044*	*oxidoreductase putative*												
PG1172		2.25 E-4	3.4	23.5	-2.7795	4.7507	G	G	3.1 E-8	1.97 E+6	9.27 E+6	-2.2320	23.4221	G
PGN1269	*PG1045*	*iron-sulfur cluster binding protein putative*												
PG1173		2.96 E-1	18.8	12	0.6496	4.9460	Y	Y	1.13 E-1	9.14 E+6	7.77 E+6	0.2357	24.0113	Y
PGN1270	*PG1046*	*YkgG family protein*												

TIGR		PP PPC / Pg Spectral Count						PP PPC/ Pg Protein Intensity						33277
PGN	*LANL*	*q*-value	PP (Norm)	PPC (Norm)	log_2Ratio	log_2Sum	Regulation	*q*-value	PP (Norm)	PPC (Norm)	log_2Ratio	log_2Sum	Regulation	Consensus
PG1174		2.8 E-1	11.1	18	-0.6944	4.8641	Y	3.45 E-3	2.08 E+6	3.3 E+6	-0.6674	22.3596	Y	Y
PGN1271	*PG1047*	*thioesterase family protein*												
PG1058		1.46 E-2	6.8	21.5	-1.6512	4.8250	Y	2.35 E-3	5.3 E+6	8.98 E+6	-0.7603	23.7679	Y	Y
PGN1296	*PG0941*	*OmpA family protein*												
PG1057		6.55 E-1	5.1	6	-0.2249	3.4769	Y	1.04 E-1	1.86 E+6	1.22 E+6	0.6019	21.5557	Y	Y
PGN1297	*PG0940*	*conserved hypothetical protein*												
PG1056		4.75 E-1	3.4	5.5	-0.6843	3.1575	Y	3.63 E-2	1.02 E+6	1.78 E+6	-0.8099	21.4180	Y	Y
PGN1298	*PG0939*	*conserved hypothetical protein*												
PG1050		1.49 E-2	12.0	2	2.5825	3.8052	Y	0	2.99 E+6	7.99 E+4	5.2262	21.5501	R	R
PGN1303	*PG0934*	*hypothetical protein PG_1050*												
PG1049		1.6 E-1	18.0	29.5	-0.7152	5.5689	Y	8.5 E-8	7.44 E+6	2.06 E+7	-1.4691	24.7408	G	G
PGN1304	*PG0933*	*conserved hypothetical protein*												
PG1048		6.69 E-1	1.7	2	-0.2249	1.8919	Y	0	3.92 E+5	7.32 E+5	-0.9010	20.0993	G	G
PGN1305	*PG0932*	*N-acetylmuramoyl-L-alanine amidase family 3*												
PG1044		9.48 E-2	1.7	7.5	-2.1318	3.2034	Y	6.6 E-6	1.85 E+3	2.34 E+6	-10.3030	21.1579	G	G
PGN1308	*PG0931*	*iron dependent repressor putative*												
PG1042		6.69 E-1	1.7	1.5	0.1901	1.6832	Y	0	1.85 E+3	1.32 E+5	-6.1601	17.0338	G	Y
PGN1310	*PG0929*	*glycogen synthase putative*												
PG1041		6.69 E-1	1.7	1.5	0.1901	1.6832	Y	0	1.85 E+3	2.1 E+6	-10.1462	21.0012	G	Y
PGN1311	*PG0928*	*K+-dependent Na+/Ca+ exchanger related-protein*												
PG1039		5.43 E-1	3.4	5	-0.5468	3.0743	Y	1.6 E-1	1.11 E+6	1.19 E+6	-0.1029	21.1349	Y	Y
PGN1313	*PG0926*	*integral membrane protein*												
PG1038		1.53 E-1	2.6	8	-1.6399	3.4015	Y	1.76 E-2	6.84 E+5	1.55 E+6	-1.1791	21.0913	Y	Y
PGN1314	*PG0925*	*ATP-dependent DNA helicase UvrD/PcrA/Rep Family*												
PG1037		0	298.6	107.5	1.4740	8.6658	R	0	2.47 E+8	9.1 E+7	1.4385	28.3314	R	R
PGN1315	*PG0924*	*hypothetical protein PG_1037*												
PG1036		6.97 E-1	5.1	5	0.0381	3.3411	Y	6.54 E-3	9.28 E+5	1.65 E+6	-0.8342	21.2999	Y	Y
PGN1316	*PG0923*	*uvrA-1 excinuclease ABC A subunit*												
PG1035		1.42 E-1	6.8	15	-1.1318	4.4492	Y	8.08 E-6	2.35 E+6	7.45 E+6	-1.6636	23.2237	G	G
PGN1317	*PG0922*	*hypothetical protein PG_1035*												
PG1034		4.65 E-1	2.6	4.5	-0.8099	2.8211	Y	8.69 E-8	1.72 E+6	5.92 E+5	1.5355	21.1387	R	Y
PGN1318	*PG0921*	*ABC transporter ATP-binding protein*												
PG1030		4.28 E-1	25.7	32	-0.3180	5.8497	Y	2.87 E-2	1.22 E+7	2.05 E+7	-0.7472	24.9626	Y	Y
PGN1321	*PG0917*	*hypothetical protein PG_1030*												

TIGR		PP PPC / Pg Spectral Count							PP PPC/ Pg Protein Intensity					33277
PGN	*LANL*	*q*-value	PP (Norm)	PPC (Norm)	log_2Ratio	log_2Sum	Regulation		*q*-value	PP (Norm)	PPC (Norm)	log_2Ratio	log_2Sum	Consensus
PG1029		5.89 E-3	11.1	1	3.4755	3.5997	Y	R	2.77 E-14	5.24 E+5	1 E+3	9.0343	19.0028	R
PGN1322		*hypothetical protein PG_1029*												
PG1028		4.61 E-3	199.4	140	0.5100	8.4067	R	Y	1.5 E-1	1.65 E+8	1.72 E+8	-0.0574	28.3278	Y
PGN1323	*PG0916*	*TPR domain protein*												
		1.59 E-1	8.6	17	-0.9904	4.6756	Y	Y	2.94 E-2	2.82 E+6	5.08 E+6	-0.8505	22.9126	Y
PGN1326														
		2.8 E-2	31.7	14.5	1.1266	5.5285	Y	R	5.01 E-9	1.27 E+7	3.6 E+6	1.8181	23.9586	R
PGN1327														
		6.41 E-1	73.6	70	0.0721	7.1658	Y	Y	3.8 E-2	3.83 E+7	3.08 E+7	0.3144	26.0414	Y
PGN1328														
PG0685		6.97 E-1	2.6	2.5	0.0381	2.3411	Y	G	0	8.52 E+5	1 E+6	-0.2386	20.8242	Y
PGN1329	*PG0618*	*ABC transporter ATP-binding protein*												
PG0628		4.23 E-1	2.6	1	1.3601	1.8347	Y	R	0	1.96 E+6	1 E+3	10.9341	20.9006	R
PGN1330	*PG0566*	*ABC transporter ATP-binding protein*												
PG1022		6.69 E-1	1.7	2	-0.2249	1.8919	Y	Y	6.86 E-2	1.85 E+3	1.35 E+6	-9.5127	20.3684	Y
PGN1334	*PG0911*	*hypothetical protein PG_1022*												
PG1020		5.43 E-1	6.8	5	0.4532	3.5662	Y	Y	2.89 E-3	1.52 E+6	9.58 E+5	0.6683	21.2423	Y
PGN1335	*PG0910*	*hypothetical protein PG_1020*												
PG1017		1.11 E-8	559.6	373.5	0.5833	9.8659	R	R	0	4.87 E+8	1.35 E+8	1.8526	29.2117	R
PGN1338	*PG0907*	*ppdK pyruvate phosphate dikinase*												
PG1014		6 E-1	39.4	36	0.1287	6.2357	Y	Y	1.03 E-1	1.39 E+7	1.21 E+7	0.1995	24.6366	Y
PGN1340	*PG0906*	*TPR domain protein*												
PG1013		6.54 E-7	199.4	321.5	-0.6894	9.0248	G	Y	1.25 E-2	2.34 E+8	3 E+8	-0.3581	28.9931	G
PGN1341	*PG0905*	*acetyl-CoA hydrolase/transferase family protein*												
PG1012		1.05 E-1	8.6	18.5	-1.1124	4.7579	Y	Y	7.65 E-2	3.92 E+6	6.02 E+6	-0.6170	23.2450	Y
PGN1342	*PG0904*	*tRNA-i(6)A37 modification enzyme MiaB*												
PG1010		6.69 E-1	12.0	13	-0.1180	4.6427	Y	Y	2.7 E-3	6.08 E+6	1.43 E+7	-1.2319	24.2791	Y
PGN1343	*PG0903*	*ABC transporter ATP-binding protein*												
PG1006		0	12.0	200	-4.0614	7.7278	G	G	0	1.9 E+6	6.65 E+7	-5.1296	26.0275	G
PGN1347	*PG0899*	*hypothetical protein PG_1006*												
PG1004		4.33 E-1	325.1	304.5	0.0947	9.2984	Y	Y	1.61 E-1	2.88 E+8	2.95 E+8	-0.0368	29.1185	Y
PGN1349	*PG0897*	*prolyl oligopeptidase family protein*												
PG1003		6.05 E-2	1.7	8.5	-2.3124	3.3521	Y	G	3.34 E-5	1.85 E+3	1.59 E+6	-9.7430	20.5984	G
PGN1350	*PG0896*	*conserved hypothetical protein*												

TIGR		PP PPC / Pg Spectral Count						PP PPC/ Pg Protein Intensity						33277
PGN	***LANL***	*q*-value	PP (Norm)	PPC (Norm)	log_2Ratio	log_2Sum	Regulation		*q*-value	PP (Norm)	PPC (Norm)	log_2Ratio	log_2Sum	Consensus
PG1001		4.75 E-1	6.8	4.5	0.6052	3.5040	Y	Y	7.29 E-2	1.4 E+6	8.21 E+6	-2.5553	23.1961	Y
PGN1352	*PG0894*	*conserved hypothetical protein*												
PG1215		3.5 E-1	7.7	12.5	-0.6988	4.3363	Y	Y	2.18 E-3	2.33 E+6	4.08 E+6	-0.8107	22.6113	Y
PGN1354	*PG1076*	*lipoprotein protein putative*												
PG1217		2.52 E-6	27.4	2	3.7751	4.8768	R	R	1.31 E-9	2.26 E+7	4.88 E+5	5.5336	24.4614	R
PGN1356	*PG1077*	*hypothetical protein PG_1217*												
PG1219		3.93 E-1	42.8	34.5	0.3104	6.2721	Y	Y	9.75 E-2	4.34 E+7	5.27 E+7	-0.2822	26.5181	Y
PGN1358	*PG1079*	*hypothetical protein PG_1219*												
PG1220		4.94 E-3	2.6	15.5	-2.5941	4.1753	G	G	3.05 E-5	1.57 E+6	5.42 E+6	-1.7833	22.7376	G
PGN1359	*PG1081*	*erythronate-4-phosphate dehydrogenase putative*												
PG1224		6.69 E-1	1.7	2	-0.2249	1.8919	Y	G	8.9 E-10	1.85 E+3	1.72 E+5	-6.5412	17.4103	G
PGN1362	*PG1083*													
PG1225		5.89 E-1	1.7	1	0.7751	1.4390	Y	R	0	3.1 E+5	1 E+3	8.2764	18.2469	R
PGN1363	*PG1084*	*ABC transporter ATP-binding protein*												
PG1226		6.69 E-1	3.4	3	0.1901	2.6832	Y	Y	3.48 E-2	7.49 E+5	1.11 E+6	-0.5637	20.8237	Y
PGN1364	*PG1085*	*peptidyl-prolyl cis-trans isomerase cyclophilin-type*												
PG1230		7 E-1	36.8	37	-0.0081	6.2054	Y	Y	1.55 E-1	1.72 E+7	1.85 E+7	-0.1043	25.0871	Y
PGN1366	*PG1089*	*hypothetical protein PG_1230*												
PG1232		1.3 E-9	2892.1	3405	-0.2355	12.6205	G	Y	1.42 E-2	4.72 E+9	5.38 E+9	-0.1904	33.2331	G
PGN1367	*PG1090*	*gdh glutamate dehydrogenase NAD-specific*												
PG1235		3.42 E-7	166.0	80.5	1.0441	7.9454	R	R	0	1.34 E+8	5.42 E+7	1.3060	27.4866	R
PGN1370	*PG1091*	*epimerase/reductase putative*												
PG1236		5.25 E-3	51.3	24	1.0970	6.2353	R	R	8.23 E-9	1.35 E+7	5.87 E+6	1.2074	24.2103	R
PGN1372	*PG1092*	*hypothetical protein PG_1236*												
PG1237		6.85 E-1	4.3	4.5	-0.0729	3.1339	Y	Y	1.25 E-1	1.44 E+6	1.86 E+6	-0.3709	21.6520	Y
PGN1373	*PG1093*	*transcriptional regulator LuxR family*												
PG1239		7.32 E-8	106.1	38	1.4814	7.1709	R	R	4.07 E-11	1 E+8	3.3 E+7	1.6034	26.9912	R
PGN1375	*PG1095*	*fabG 3-oxoacyl-(acyl-carrier-protein) reductase*												
PG1240		5.17 E-1	7.7	5.5	0.4856	3.7226	Y	Y	6.1 E-3	2.39 E+6	1.35 E+6	0.8292	21.8350	Y
PGN1376	*PG1096*	*transcriptional regulator tetR family*												
PG1241		4.07 E-2	12.0	3	1.9975	3.9049	Y	R	0	7.46 E+6	7.81 E+5	3.2555	22.9748	R
PGN1377	*PG1097*	*lepA GTP-binding protein Lepa*												
PG1242		9.44 E-2	6.0	1	2.5825	2.8052	Y	R	0	1.44 E+6	1 E+3	10.4938	20.4606	R
PGN1378	*PG1098*	*dnaB replicative DNA helicase*												

TIGR		PP PPC / Pg Spectral Count							PP PPC/ Pg Protein Intensity					33277
PGN	***LANL***	*q*-value	PP (Norm)	PPC (Norm)	log_2Ratio	log_2Sum	Regulation		*q*-value	PP (Norm)	PPC (Norm)	log_2Ratio	log_2Sum	Consensus
PG1246		1.13 E-4	178.8	107	0.7410	8.1590	R	R	9.96 E-11	1.14 E+8	6.31 E+7	0.8558	27.4016	R
PGN1381	*PG1101*	*alaS alanyl-tRNA synthetase*												
PG1247		5.17 E-1	1.7	3	-0.8099	2.2361	Y	G	2.67 E-7	1.85 E+3	2.98 E+5	-7.3331	18.1957	G
PGN1382	*PG1102*	*aroB 3-dehydroquinate synthase*												
PG1248		6.54 E-1	8.6	7.5	0.1901	4.0051	Y	Y	1.53 E-2	2.48 E+6	1.47 E+6	0.7539	21.9141	Y
PGN1383	*PG1103*	*conserved hypothetical protein*												
PG1249		4.21 E-1	3.4	1.5	1.1901	2.2994	Y	R	0	1.93 E+6	1.51 E+5	3.6762	20.9920	R
PGN1384	*PG1104*	*1-acyl-sn-glycerol-3-phosphate acetyltransferase putative*												
PG1252		6.69 E-1	1.7	1.5	0.1901	1.6832	Y	G	0	1.85 E+3	7.65 E+4	-5.3694	16.2576	Y
PGN1387	*PG1106*	*membrane protein putative*												
PG1253		5.43 E-1	3.4	5	-0.5468	3.0743	Y	Y	6.08 E-3	2.31 E+5	1.05 E+6	-2.1830	20.2874	Y
PGN1388	*PG1107*	*ligA DNA ligase NAD-dependent*												
PG1255		6.69 E-1	1.7	1.5	0.1901	1.6832	Y	G	0	1.85 E+3	1.12 E+5	-5.9249	16.8021	Y
PGN1390	*PG1109*	*recR recombination protein RecR*												
PG1256		7.01 E-1	17.1	17	0.0096	5.0923	Y	R	2.2 E-4	6.47 E+6	3.09 E+6	1.0679	23.1876	R
PGN1391	*PG1110*	*ribonuclease Rne/Rng family*												
PG1258		3.25 E-1	34.2	25.5	0.4246	5.9003	Y	Y	1.73 E-1	5.37 E+7	5.07 E+7	0.0847	26.6371	Y
PGN1393	*PG1111*	*hup-2 DNA-binding protein HU*												
PG1260		1.67 E-1	292.6	254	0.2043	9.0944	Y	Y	9.02 E-3	1.97 E+8	2.48 E+8	-0.3301	28.7278	Y
PGN1396	*PG1113*	*anaerobic ribonucleoside-triphosphate reductase putative*												
PG1269		6.69 E-1	309.7	315	-0.0243	9.2871	Y	Y	1.34 E-2	4 E+8	5 E+8	-0.3237	29.7450	Y
PGN1401	*PG1120*	*pruA delta-1-pyrroline-5-carboxylate dehydrogenase*												
PG1270		4.62 E-3	29.1	9.5	1.6146	5.2702	R	R	0	2.36 E+7	3.07 E+6	2.9445	24.6687	R
PGN1402	*PG1121*	*conserved hypothetical protein*												
PG1271		1.55 E-3	325.1	422.5	-0.3779	9.5462	G	Y	8.93 E-3	2.8 E+8	3.64 E+8	-0.3794	29.2632	G
PGN1403	*PG1122*	*acetylornithine aminotransferase putative*												
PG0562		4.23 E-1	2.6	1	1.3601	1.8347	Y	R	0	7.42 E+5	1 E+3	9.5344	19.5021	R
PGN1408	*PG0508*	*potassium uptake protein TrkA putative*												
PG0561		5.8 E-1	182.3	173.5	0.0710	8.4747	Y	Y	2.95 E-2	1.65 E+8	2.02 E+8	-0.2949	28.4534	Y
PGN1409	*PG0507*	*peptidase M20/M25/M40 family*												
PG0559		1.53 E-1	2.6	8	-1.6399	3.4015	Y	G	4.39 E-6	1 E+6	6.45 E+6	-2.6859	22.8292	G
PGN1411	*PG0506*	*chlorohydrolase family protein*												
PG0558		1.21 E-5	67.6	135	-0.9979	7.6625	G	Y	1.81 E-3	3.32 E+7	6.1 E+7	-0.8758	26.4901	G
PGN1412	*PG0505*	*purine nucleoside phosphorylase I inosine and guanosine-specific*												

TIGR		PP PPC / Pg Spectral Count						PP PPC/ Pg Protein Intensity							33277
PGN	*LANL*	*q*-value	PP (Norm)	PPC (Norm)	log_2Ratio	log_2Sum	Regulation		*q*-value	PP (Norm)	PPC (Norm)	log_2Ratio	log_2Sum	Consensus	
PG0555		5.47 E-3	4.3	19	-2.1509	4.5409	G	Y	2.65 E-2	7.41 E+5	1.15 E+6	-0.6364	20.8531	G	
PGN1415	*PG0502*	*DNA-binding protein histone-like family*													
PG0553		5.63 E-4	312.3	416	-0.4136	9.5084	G	Y	1.59 E-3	1.89 E+8	2.67 E+8	-0.4976	28.7643	G	
PGN1416	*PG0501*	*extracellular protease putative*													
PG0548		1.1 E-1	328.6	281.5	0.2231	9.2528	Y	R	5.48 E-4	2.27 E+8	1.62 E+8	0.4895	28.5337	R	
PGN1418	*PG0498*	*pyruvate ferredoxin/flavodoxin oxidoreductase family protein*													
PG0547		1.34 E-7	53.1	10	2.4074	5.9784	R	R	3.5 E-6	3.56 E+7	5.8 E+6	2.6176	25.3031	R	
PGN1419	*PG0497*	*conserved hypothetical protein*													
		3.82 E-1	1.7	4	-1.2249	2.5138	Y	Y	5.45 E-3	1.85 E+3	8.99 E+5	-8.9245	19.7812	Y	
PGN1426															
PG0541		6.94 E-1	7.7	7.5	0.0381	3.9261	Y	Y	1.93 E-1	3.56 E+6	3.42 E+6	0.0568	22.7352	Y	
PGN1429	*PG0494*	*hypothetical protein PG_0541*													
		3.05 E-1	18.0	11.5	0.6439	4.8811	Y	Y	1.49 E-1	9.48 E+6	8.52 E+6	0.1540	24.1020	Y	
PGN1430															
PG0539		1.98 E-1	15.4	8	0.9450	4.5485	Y	R	8.61 E-5	7.47 E+6	3.69 E+6	1.0155	23.4123	R	
PGN1431	*PG0492*	*efflux transporter MFP component RND family*													
PG0538		1.86 E-2	10.3	26.5	-1.3679	5.2004	Y	G	3.38 E-4	3.93 E+6	8.69 E+6	-1.1472	23.5890	G	
PGN1432	*PG0490*	*outer membrane efflux protein*													
PG0537		2.5 E-13	616.1	917	-0.5738	10.5822	G	Y	1.06 E-2	5.45 E+8	6.53 E+8	-0.2614	30.1571	G	
PGN1434	*PG0489*	*pepD-2 aminoacyl-histidine dipeptidase*													
PG0535		4.23 E-1	6.8	4	0.7751	3.4390	Y	Y	3.5 E-2	6.05 E+6	3.28 E+6	0.8844	23.1522	Y	
PGN1436	*PG0488*	*conserved hypothetical protein*													
PG0534		1.76 E-5	8.6	40	-2.2249	5.6016	G	G	1.62 E-12	3.3 E+6	1.49 E+7	-2.1780	24.1202	G	
PGN1437	*PG0487*	*hypothetical protein PG_0534*													
PG0532		4.4 E-1	1.7	3.5	-1.0323	2.3816	Y	Y	2.84 E-2	1.85 E+3	1.15 E+6	-9.2795	20.1355	Y	
PGN1440	*PG0486*	*conserved domain protein*													
PG0531		7.86 E-5	82.1	35.5	1.2103	6.8783	R	Y	6.61 E-2	3.18 E+7	2.34 E+7	0.4388	25.7181	R	
PGN1441	*PG0485*	*nadE glutamine-dependent NAD+ synthetase*													
PG0530		7.93 E-2	26.5	13.5	0.9744	5.3228	Y	R	5.58 E-5	1.82 E+7	7.72 E+6	1.2355	24.6259	R	
PGN1443	*PG0484*	*carB carbamoyl-phosphate synthase large subunit*													
PG0529		1.04 E-2	2.6	14	-2.4473	4.0502	Y	G	6.88 E-6	9.86 E+5	7.82 E+6	-2.9879	23.0700	G	
PGN1444	*PG0482*	*carA carbamoyl-phosphate synthase small subunit*													
PG0528		5.08 E-2	28.2	13.5	1.0646	5.3832	Y	R	3.1 E-5	1.04 E+7	4.24 E+6	1.2882	23.7996	R	
PGN1445	*PG0481*	*amidophosphoribosyltransferase putative*													

TIGR		PP PPC / Pg Spectral Count							PP PPC/ Pg Protein Intensity					33277
PGN	*LANL*	*q*-value	PP (Norm)	PPC (Norm)	log_2Ratio	log_2Sum	Regulation		*q*-value	PP (Norm)	PPC (Norm)	log_2Ratio	log_2Sum	Consensus
PG0526		1.41 E-6	24.8	1	4.6331	4.6901	R	R	0	9.83 E+6	1 E+3	13.2635	23.2294	R
PGN1446	*PG0480*	*membrane protein putative*												
PG0525		4.5 E-1	29.1	23.5	0.3080	5.7168	Y	R	1.33 E-6	1.33 E+7	5.2 E+6	1.3581	24.1441	R
PGN1447	*PG0479*	*pyrG CTP synthase*												
PG0523		1.21 E-2	127.5	85.5	0.5764	7.7347	Y	Y	8.93 E-3	4.09 E+7	5.47 E+7	-0.4212	26.5107	Y
PGN1449	*PG0477*	*guaB inosine-5'-monophosphate dehydrogenase*												
PG0521		3.86 E-1	70.2	82	-0.2249	7.2495	Y	Y	1.46 E-1	1.5 E+8	1.64 E+8	-0.1314	28.2279	Y
PGN1451	*PG0475*	*groES chaperonin 10 kDa*												
PG0520		0	779.5	384.5	1.0196	10.1849	R	R	1.01 E-15	5.51 E+8	2.76 E+8	1.0004	29.6230	R
PGN1452	*PG0474*	*groEL chaperonin 60 kDa*												
PG0517		3.05 E-1	3.4	1	1.7751	2.1449	Y	R	0	7.61 E+5	1 E+3	9.5712	19.5389	R
PGN1455	*PG0471*	*hypothetical protein PG_0517*												
PG0516		1.45 E-1	5.1	1	2.3601	2.6168	Y	R	0	9.33 E+5	1 E+3	9.8654	19.8327	R
PGN1456	*PG0470*	*conserved hypothetical protein*												
PG0515		6.22 E-1	18.0	16	0.1674	5.0861	Y	R	3.86 E-4	1.2 E+7	3.61 E+6	1.7289	23.8942	R
PGN1457	*PG0469*	*conserved hypothetical protein*												
PG0514		2.95 E-10	160.9	62.5	1.3639	7.8032	R	R	0	8.47 E+7	2.1 E+7	2.0100	26.6555	R
PGN1458	*PG0468*	*secA preprotein translocase SecA subunit*												
PG0513		9.48 E-2	1.7	7.5	-2.1318	3.2034	Y	G	1.34 E-4	1.85 E+3	1.11 E+6	-9.2318	20.0879	G
PGN1459	*PG0467*	*conserved hypothetical protein TIGR00255*												
PG0510		6.69 E-1	1.7	1.5	0.1901	1.6832	Y	G	0	1.85 E+3	2 E+5	-6.7581	17.6250	Y
PGN1462	*PG0464*	*conserved hypothetical protein*												
PG0506		6.68 E-1	1976.6	1962.5	0.0103	11.9436	Y	Y	3.96 E-2	1.11 E+9	1.29 E+9	-0.2168	31.1552	Y
PGN1466	*PG0461*	*prtRII arginine-specific cysteine proteinase*												
PG0504		3.21 E-1	1.7	4.5	-1.3948	2.6349	Y	G	3.01 E-4	1.85 E+3	6.86 E+5	-8.5348	19.3924	G
PGN1468	*PG0460*	*lipA lipoate synthase*												
PG0503		5.76 E-5	39.4	89	-1.1771	7.0041	G	G	1.33 E-4	2.26 E+7	4.65 E+7	-1.0434	26.0422	G
PGN1469	*PG0459*	*dpp dipeptidyl aminopeptidase IV*												
PG0502		1.45 E-1	5.1	1	2.3601	2.6168	Y	R	1.44 E-5	4.13 E+5	1 E+3	8.6913	18.6606	R
PGN1470	*PG0458*	*smpB SsrA-binding protein*												
PG0500		1.7 E-1	14.5	7	1.0552	4.4294	Y	Y	5.26 E-2	4.2 E+6	2.77 E+6	0.6012	22.7333	Y
PGN1472	*PG0456*	*tgt queuine tRNA-ribosyltransferase*												
PG0498		6.69 E-1	5.1	4.5	0.1901	3.2681	Y	Y	1.85 E-1	1.27 E+6	1.3 E+6	-0.0435	21.2933	Y
PGN1474	*PG0454*	*luxS autoinducer-2 production protein LuxS*												

TIGR		PP PPC / Pg Spectral Count							PP PPC/ Pg Protein Intensity					33277
PGN	***LANL***	*q*-value	PP (Norm)	PPC (Norm)	log_2Ratio	log_2Sum	Regulation		*q*-value	PP (Norm)	PPC (Norm)	log_2Ratio	log_2Sum	Consensus
PG0497		6.69 E-1	1.7	2	-0.2249	1.8919	Y	G	1.57 E-7	1.85 E+3	2.51 E+5	-7.0840	17.9483	G
PGN1475	*PG0453*	*mtn 5'-methylthioadenosine/S-adenosylhomocysteine nucleosidase*												
PG0495		8.44 E-2	4.3	12.5	-1.5468	4.0685	Y	G	2.09 E-5	1.15 E+6	3.85 E+6	-1.7364	22.2534	G
PGN1476	*PG0452*	*hypothetical protein PG_0495*												
PG0491		2.44 E-1	353.4	391.5	-0.1478	9.5409	Y	Y	1.81 E-1	3.53 E+8	3.47 E+8	0.0230	29.3819	Y
PGN1479	*PG0449*	*conserved hypothetical protein*												
PG0490		1.45 E-1	5.1	1	2.3601	2.6168	Y	R	0	3.37 E+6	1 E+3	11.7167	21.6829	R
PGN1480	*PG0448*	*membrane protein putative*												
PG0488		6.61 E-1	9.4	10.5	-0.1578	4.3156	Y	Y	2.06 E-2	2.38 E+6	4.17 E+6	-0.8069	22.6447	Y
PGN1482	*PG0446*	*ruvB Holliday junction DNA helicase RuvB*												
PG0486		3.82 E-1	1.7	4	-1.2249	2.5138	Y	G	1.28 E-4	1.85 E+3	1.84 E+6	-9.9579	20.8131	G
PGN1484	*PG0444*	*ogt methylated-DNA--protein-cysteine S-methyltransferase*												
PG0485		2.32 E-1	42.8	57	-0.4139	6.6407	Y	Y	9.87 E-2	1.51 E+7	1.77 E+7	-0.2363	24.9667	Y
PGN1485	*PG0443*	*yajC preprotein translocase YajC subunit*												
PG0484		4.23 E-1	2.6	1	1.3601	1.8347	Y	R	1.87 E-7	8.48 E+5	1 E+3	9.7284	19.6959	R
PGN1486	*PG0442*	*hypothetical protein PG_0484*												
PG0482		6.64 E-1	13.7	12.5	0.1312	4.7110	Y	Y	3.9 E-2	7.17 E+6	3.71 E+6	0.9482	23.3750	Y
PGN1488	*PG0440*	*hypothetical protein PG_0482*												
PG0481		0	367.9	122	1.5926	8.9364	R	R	1.84 E-6	1.9 E+8	1.01 E+8	0.9065	28.1189	R
PGN1489	*PG0439*	*kbl 2-amino-3-ketobutyrate CoA ligase*												
PG0477		4.14 E-1	36.8	29.5	0.3187	6.0508	Y	R	1.42 E-5	1.63 E+7	8.82 E+6	0.8862	24.5830	R
PGN1492	*PG0436*	*panC pantoate--beta-alanine ligase*												
PG0476		4.75 E-1	18.0	22.5	-0.3244	5.3387	Y	Y	9.88 E-2	9.27 E+6	7.61 E+6	0.2832	24.0089	Y
PGN1493	*PG0435*	*yngK-2 yngK protein*												
PG0474		6.69 E-1	1.7	2	-0.2249	1.8919	Y	Y	1.05 E-1	4.32 E+5	6.36 E+5	-0.5581	20.0267	Y
PGN1495	*PG0433*	*low-specificity L-threonine aldolase*												
PG0471		6.69 E-1	2.6	3	-0.2249	2.4769	Y	Y	1.8 E-2	1.04 E+6	3.99 E+6	-1.9438	22.2600	Y
PGN1498	*PG0429*	*hypothetical protein PG_0471*												
PG0468		3.09 E-1	9.4	5	0.9126	3.8492	Y	Y	2.19 E-3	4.31 E+6	2.38 E+6	0.8548	22.6749	Y
PGN1501	*PG0427*	*manA mannose-6-phosphate isomerase class I*												
PG0466		1.16 E-1	5.1	13	-1.3404	4.1806	Y	Y	1.57 E-2	1.98 E+6	5.16 E+6	-1.3814	22.7667	Y
PGN1502	*PG0426*	*hypothetical protein PG_0466*												
PG0465		6.05 E-2	6.8	1	2.7751	2.9718	Y	R	4.08 E-8	2.08 E+6	1 E+3	11.0250	20.9915	R
PGN1503	*PG0425*	*fur ferric uptake transcriptional regulator*												

TIGR		PP PPC / Pg Spectral Count							PP PPC/ Pg Protein Intensity					33277
PGN	***LANL***	*q*-value	PP (Norm)	PPC (Norm)	log_2Ratio	log_2Sum	Regulation		*q*-value	PP (Norm)	PPC (Norm)	log_2Ratio	log_2Sum	Consensus
PG0464		3.01 E-1	82.1	98.5	-0.2620	7.4970	Y	Y	1.36 E-1	6.39 E+7	6.77 E+7	-0.0832	26.9720	Y
PGN1504	*PG0424*	*purA adenylosuccinate synthetase*												
PG0463		6 E-1	8.6	7	0.2897	3.9594	Y	R	6.37 E-4	2.67 E+6	9.68 E+5	1.4649	21.7955	R
PGN1505	*PG0423*	*folC folylpolyglutamate synthase*												
PG0462		2.51 E-6	13.7	54.5	-1.9931	6.0915	G	G	6.92 E-6	8.87 E+6	2.4 E+7	-1.4365	24.9709	G
PGN1506	*PG0422*	*transporter putative*												
PG0452		3.11 E-4	243.0	162.5	0.5805	8.6636	R	R	0	3.08 E+8	1.36 E+8	1.1782	28.7261	R
PGN1510	*PG0414*	*conserved hypothetical protein*												
PG0451		6.69 E-1	1.7	1.5	0.1901	1.6832	Y	G	0	1.85 E+3	1.18 E+5	-5.9917	16.8679	Y
PGN1511	*PG0413*	*CBS domain protein*												
PG0450		3.82 E-1	1.7	4	-1.2249	2.5138	Y	G	1.28 E-5	1.85 E+3	4.43 E+5	-7.9019	18.7616	G
PGN1512	*PG0412*	*hypothetical protein PG_0450*												
PG0449		2.61 E-9	187.4	84	1.1576	8.0842	R	R	1.16 E-11	1.79 E+8	7.28 E+7	1.2966	27.9076	R
PGN1513	*PG0411*	*TPR domain protein*												
PG0448		2.24 E-8	39.4	111.5	-1.5022	7.2371	G	G	5.49 E-14	1.13 E+7	5.32 E+7	-2.2400	25.9432	G
PGN1514	*PG0410*	*hypothetical protein PG_0448*												
PG0447		2.26 E-1	6.0	2	1.5825	2.9981	Y	R	3.51 E-5	2.54 E+6	5.3 E+5	2.2611	21.5487	R
PGN1515	*PG0409*	*conserved hypothetical protein*												
PG0446		4.23 E-1	2.6	1	1.3601	1.8347	Y	R	0	3.8 E+5	1 E+3	8.5708	18.5404	R
PGN1516	*PG0408*	*thiF thiF protein*												
PG0445		2.52 E-1	57.3	73	-0.3486	7.0260	Y	Y	3.17 E-3	5.66 E+7	9.71 E+7	-0.7788	27.1950	Y
PGN1517	*PG0407*	*pepT peptidase T*												
PG0444		6.06 E-1	10.3	12	-0.2249	4.4769	Y	Y	7.97 E-2	8.14 E+6	6.97 E+6	0.2251	23.8488	Y
PGN1518	*PG0406*	*oligopeptide transporter OPT family*												
PG0443		3.68 E-1	92.4	79	0.2262	7.4213	Y	Y	1.76 E-2	6.76 E+7	4.84 E+7	0.4830	26.7902	Y
PGN1519	*PG0405*	*hemagglutinin-related protein*												
PG0441		1.34 E-2	1.7	11.5	-2.7485	3.7237	Y	G	3.96 E-8	1.85 E+3	1.89 E+6	-9.9958	20.8509	G
PGN1520	*PG0404*	*hypothetical protein PG_0441*												
PG0438		6.69 E-1	1.7	1.5	0.1901	1.6832	Y	G	0	1.85 E+3	3.53 E+5	-7.5771	18.4383	Y
PGN1522	*PG0401*	*hypothetical protein PG_0438*												
PG0437		7.69 E-2	8.6	2	2.0970	3.4001	Y	R	4.86 E-5	7.6 E+6	6.01 E+5	3.6611	22.9674	R
PGN1523	*PG0400*	*polysaccharide export protein BexD/CtrA/VexA family*												
PG0436		1.18 E-1	31.7	18.5	0.7751	5.6484	Y	Y	8.37 E-2	1.31 E+7	1.62 E+7	-0.3082	24.8042	Y
PGN1524	*PG0399*	*capsular polysaccharide transport protein putative*												

TIGR		PP PPC / Pg Spectral Count							PP PPC/ Pg Protein Intensity						33277
PGN	***LANL***	*q*-value	PP (Norm)	PPC (Norm)	log_2Ratio	log_2Sum	Regulation		*q*-value	PP (Norm)	PPC (Norm)	log_2Ratio	log_2Sum	Consensus	
PG0435		1.5 E-1	1.7	6.5	-1.9253	3.0376	Y	G	8 E-4	1.85 E+3	2.44 E+6	-10.3676	21.2224	G	
PGN1525	*PG0398*	*capsular polysaccharide biosythesis protein putative*													
PG0434		5.17 E-1	1.7	3	-0.8099	2.2361	Y	Y	1 E-2	1.85 E+3	3.77 E+5	-7.6692	18.5300	Y	
PGN1526	*PG0397*	*hypothetical protein PG_0434*													
PG0433		5.72 E-1	12.8	10.5	0.2897	4.5444	Y	Y	1.45 E-2	2.63 E+6	3.64 E+6	-0.4697	22.5809	Y	
PGN1527	*PG0396*	*tetrapyrrole methylase family protein*													
PG0432		6 E-1	3.4	4.5	-0.3948	2.9860	Y	Y	8.66 E-2	6.49 E+5	4.77 E+5	0.4438	20.1021	Y	
PGN1528	*PG0395*	*NOL1/NOP2/sun family protein*													
PG0430		1.75 E-3	112.9	66	0.7751	7.4834	R	R	2.27 E-4	7.77 E+7	4.92 E+7	0.6599	26.9188	R	
PGN1529	*PG0394*	*oxidoreductase putative*													
PG0429		0	326.9	104	1.6521	8.7511	R	R	1.13 E-4	1.47 E+8	8.77 E+7	0.7447	27.8058	R	
PGN1530	*PG0393*	*pyruvate synthase*													
		4 E-3	23.1	6	1.9450	4.8631	R	R	5.6 E-7	2.32 E+7	8.24 E+6	1.4941	24.9073	R	
PGN1533															
		5.43 E-1	6.8	5	0.4532	3.5662	Y	Y	1.97 E-3	1.19 E+6	5.44 E+5	1.1304	20.7255	Y	
PGN1534															
		1.6 E-1	11.1	20.5	-0.8820	4.9829	Y	Y	1.61 E-1	5.83 E+6	5.48 E+6	0.0885	23.4308	Y	
PGN1535															
		6.97 E-1	3.4	3.5	-0.0323	2.7913	Y	Y	3.32 E-2	1.33 E+6	7.66 E+5	0.7915	20.9971	Y	
PGN1536															
		4.4 E-1	1.7	3.5	-1.0323	2.3816	Y	G	1.85 E-8	1.85 E+3	3.5 E+5	-7.5614	18.4227	G	
PGN1538															
		2.04 E-2	4.3	16	-1.9030	4.3419	Y	Y	1.69 E-3	2.2 E+6	4.73 E+6	-1.1033	22.7236	Y	
PGN1539															
PG0540		5.43 E-1	2.6	1.5	0.7751	2.0240	Y	R	0	2.31 E+6	3.39 E+5	2.7728	21.3395	R	
PGN1540	*PG0493*	*AcrB/AcrD/AcrF family protein*													
PG0423		3.52 E-1	12.8	8	0.6820	4.3809	Y	R	4.53 E-8	1.17 E+7	4.62 E+6	1.3422	23.9607	R	
PGN1544	*PG0388*	*hypothetical protein PG_0423*													
PG0421		1.32 E-4	28.2	5	2.4976	5.0547	R	R	6.33 E-11	1.14 E+7	2.03 E+6	2.4893	23.6810	R	
PGN1547	*PG0386*	*hypothetical protein PG_0421*													
PG0418		2.8 E-1	40.2	52.5	-0.3846	6.5347	Y	Y	1.83 E-2	2.81 E+7	3.94 E+7	-0.4877	26.0076	Y	
PGN1549	*PG0384*	*clpP ATP-dependent Clp protease proteolytic subunit*													
PG0417		4.45 E-1	6.0	9	-0.5875	3.9059	Y	Y	2.59 E-2	1.74 E+6	2.92 E+6	-0.7454	22.1537	Y	
PGN1550	*PG0382*	*clpX ATP-dependent Clp protease ATP-binding subunit ClpX*													

TIGR		PP PPC / Pg Spectral Count							PP PPC/ Pg Protein Intensity						33277
PGN	***LANL***	*q*-value	PP (Norm)	PPC (Norm)	log_2Ratio	log_2Sum	Regulation			*q*-value	PP (Norm)	PPC (Norm)	log_2Ratio	log_2Sum	Consensus
PG0416		8.91 E-2	34.2	19.5	0.8116	5.7476	Y		Y	4.97 E-3	1.34 E+7	6.83 E+6	0.9689	24.2683	Y
PGN1551	*PG0381*	*recQ-1 ATP-dependent DNA helicase RecQ*													
PG0415		1.63 E-8	48.8	6.5	2.9075	5.7885	R		R	0	2.15 E+7	3.49 E+6	2.6203	24.5729	R
PGN1552	*PG0380*	*peptidyl-prolyl cis-trans isomerase PPIC-type*													
PG0414		9.48 E-2	1.7	7.5	-2.1318	3.2034	Y		G	1.72 E-8	1.85 E+3	1.52 E+6	-9.6863	20.5417	G
PGN1553	*PG0379*	*hypothetical protein PG_0414*													
PG0413		6.69 E-1	1.7	2	-0.2249	1.8919	Y		G	1.79 E-13	1.85 E+3	2.31 E+5	-6.9630	17.8282	G
PGN1554	*PG0378*	*hypothetical protein PG_0413*													
PG0412		2.74 E-1	1.7	5	-1.5468	2.7466	Y		G	6.76 E-5	3.93 E+4	1.34 E+6	-5.0912	20.3963	G
PGN1555	*PG0377*	*mutL DNA mismatch repair protein MutL*													
PG0411		6.33 E-1	12.8	14.5	-0.1760	4.7727	Y		Y	1.21 E-1	4.61 E+6	5.26 E+6	-0.1903	23.2344	Y
PGN1556	*PG0376*	*hemagglutinin putative*													
PG0409		3.53 E-6	38.5	96	-1.3180	7.0715	G		G	2.24 E-5	1.81 E+7	4.87 E+7	-1.4268	25.9934	G
PGN1557	*PG0373*	*hypothetical protein PG_0409*													
PG0401		6.35 E-1	65.0	61.5	0.0805	6.9833	Y		Y	9.98 E-2	3.6 E+7	2.96 E+7	0.2830	25.9681	Y
PGN1564	*PG0368*	*KH/HDIG domain protein*													
PG0400		2.22 E-1	7.7	3	1.3601	3.4197	Y		R	8.86 E-8	1.01 E+6	4.82 E+5	1.0731	20.5124	R
PGN1565	*PG0367*	*conserved hypothetical protein*													
PG0398		5.17 E-1	7.7	5.5	0.4856	3.7226	Y		R	9.34 E-6	1.14 E+6	3.37 E+5	1.7623	20.4985	R
PGN1567	*PG0365*	*recF recF protein*													
PG0395		0	348.3	103.5	1.7505	8.8194	R		R	0	2.69 E+8	5.1 E+7	2.3992	28.2542	R
PGN1570	*PG0361*	*rpoC DNA-directed RNA polymerase beta' subunit*													
PG0394		0	262.7	68.5	1.9392	8.3715	R		R	0	1.82 E+8	2.99 E+7	2.6101	27.6620	R
PGN1571	*PG0360*	*rpoB DNA-directed RNA polymerase beta subunit*													
PG0393		2.04 E-1	432.1	388.5	0.1535	9.6805	Y		Y	2.02 E-1	8.05 E+8	8.05 E+8	-0.0005	30.5839	Y
PGN1572	*PG0359*	*rplL ribosomal protein L7/L12*													
PG0392		6.8 E-2	251.6	205.5	0.2918	8.8362	Y		R	2.37 E-4	1.88 E+8	1.2 E+8	0.6434	28.2008	R
PGN1573	*PG0358*	*rplJ ribosomal protein L10*													
PG0391		0	308.9	65.5	2.2375	8.5484	R		R	3.98 E-11	1.87 E+8	7.24 E+7	1.3675	27.9499	R
PGN1574	*PG0357*	*rplA ribosomal protein L1*													
PG0390		1.89 E-3	87.3	47	0.8929	7.0691	R		Y	1.12 E-2	1.36 E+8	9.63 E+7	0.5028	27.7947	R
PGN1575	*PG0356*	*rplK ribosomal protein L11*													
PG0389		3.11 E-4	52.2	19	1.4579	6.1537	R		R	1.87 E-12	2.54 E+7	6.38 E+6	1.9944	24.9235	R
PGN1576	*PG0355*	*nusG transcription antitermination protein NusG*													

TIGR	PGN	LANL	Description	PP PPC / Pg Spectral Count q-value	PP (Norm)	PPC (Norm)	log_2Ratio	log_2Sum	Regulation (Spectral Count)	Regulation (Protein Intensity)	PP PPC/ Pg Protein Intensity q-value	PP (Norm)	PPC (Norm)	log_2Ratio	log_2Sum	33277 Consensus
	PGN1577			4.02 E-1	10.3	6.5	0.6596	4.0676	Y	Y	3.87 E-2	9.35 E+6	6.31 E+6	0.5680	23.9003	Y
PG0387	PGN1578	PG0353	*tuf translation elongation factor Tu*	0	1491.4	695.5	1.1005	11.0947	R	R	0	2.28 E+9	9.23 E+8	1.3054	31.5780	R
PG0386	PGN1579	PG0352	*site-specific recombinase phage integrase family/ribosomal subunit interface protein*	1.34 E-4	38.5	10	1.9450	5.6000	R	R	3.13 E-13	4.37 E+7	4.62 E+6	3.2413	25.5253	R
PG0384	PGN1581	PG0350	*MutS2 family protein*	6 E-1	4.3	5.5	-0.3624	3.2896	Y	Y	1.95 E-1	1.32 E+6	1.3 E+6	0.0278	21.3202	Y
PG0383	PGN1582	PG0349	*membrane-associated zinc metalloprotease putative*	1.52 E-2	9.4	1	3.2345	3.3802	Y	R	0	2.6 E+6	1 E+3	11.3440	21.3103	R
PG0380	PGN1585	PG0346	*uvrB excinuclease ABC B subunit*	1.26 E-1	2.6	8.5	-1.7274	3.4682	Y	Y	1.67 E-1	8.98 E+5	8.38 E+5	0.0996	20.7269	Y
PG0378	PGN1587	PG0345	*tsf translation elongation factor Ts*	0	195.9	38	2.3664	7.8700	R	R	0	1.8 E+8	3.34 E+7	2.4337	27.6704	R
PG0377	PGN1588	PG0344	*rpsB ribosomal protein S2*	0	276.4	106	1.3826	8.5788	R	R	0	2.15 E+8	4.12 E+7	2.3839	27.9312	R
PG0376	PGN1589	PG0343	*rpsI ribosomal protein S9*	5.43 E-1	21.4	25	-0.2249	5.5358	Y	Y	2.84 E-2	1.78 E+7	1.28 E+7	0.4727	24.8652	Y
PG0375	PGN1590	PG0342	*rplM ribosomal protein L13*	2.87 E-1	81.3	66	0.3006	7.2025	Y	R	1.84 E-5	4.32 E+7	1.84 E+7	1.2322	25.8751	R
PG0373	PGN1591	PG0341	*hypothetical protein PG_0373*	5.17 E-1	1.7	3	-0.8099	2.2361	Y	Y	1.5 E-3	1.85 E+3	1.04 E+6	-9.1288	19.9851	Y
PG0369	PGN1593	PG0340	*coaD phosphopantetheine adenylyltransferase*	4.4 E-1	1.7	3.5	-1.0323	2.3816	Y	G	0	1.85 E+3	5.58 E+5	-8.2351	19.0935	G
PG0368	PGN1594	PG0339	*DNA topoisomerase IV B subunit putative*	1.4 E-1	14.5	6.5	1.1621	4.3955	Y	Y	2.5 E-3	6.44 E+6	2 E+6	1.6860	23.0088	Y
PG0366	PGN1595	PG0338	*hypothetical protein PG_0366*	2.74 E-1	8.6	4	1.0970	3.6504	Y	R	6.45 E-7	3.29 E+6	1.01 E+6	1.7055	22.0364	R
PG0365	PGN1596	PG0337	*3'-5' exonuclease domain protein*	5.39 E-1	5.1	3.5	0.5527	3.1100	Y	R	2.92 E-4	1.66 E+6	5.2 E+5	1.6763	21.0567	R
PG0364	PGN1597	PG0336	*conserved hypothetical protein*	2.14 E-1	3.4	8.5	-1.3124	3.5756	Y	Y	1.68 E-2	1.63 E+6	3.35 E+6	-1.0406	22.2484	Y
PG0362	PGN1599	PG0334	*hypothetical protein PG_0362*	1.48 E-4	6.8	32	-2.2249	5.2797	G	Y	5.91 E-3	5.32 E+6	1.03 E+7	-0.9605	23.9008	G

TIGR		PP PPC / Pg Spectral Count							PP PPC/ Pg Protein Intensity					33277
PGN	*LANL*	*q*-value	PP (Norm)	PPC (Norm)	log_2Ratio	log_2Sum	Regulation		*q*-value	PP (Norm)	PPC (Norm)	log_2Ratio	log_2Sum	Consensus
PG0361		4.23 E-1	3.4	6	-0.8099	3.2361	Y	Y	7.19 E-3	8 E+5	1.83 E+6	-1.1907	21.3238	Y
PGN1600	*PG0333*	*conserved domain protein*												
PG0360		5.84 E-4	47.9	17.5	1.4532	6.0316	R	R	5.19 E-10	2.92 E+7	9.06 E+6	1.6899	25.1910	R
PGN1601	*PG0332*	*lemA protein*												
PG0359		3.21 E-1	1.7	4.5	-1.3948	2.6349	Y	G	4.4 E-8	1.85 E+3	4.86 E+6	-11.3588	22.2131	G
PGN1602	*PG0331*	*flavin reductase domain protein*												
PG0358		4.23 E-1	2.6	1	1.3601	1.8347	Y	R	0	2.64 E+5	1 E+3	8.0465	18.0177	R
PGN1603	*PG0330*	*pyrI aspartate carbamoyltransferase regulatory subunit*												
PG0357		1.56 E-1	28.2	42.5	-0.5899	6.1444	Y	Y	8.6 E-3	9.98 E+6	1.63 E+7	-0.7087	24.6485	Y
PGN1604	*PG0329*	*pyrB aspartate carbamoyltransferase catalytic subunit*												
PG0356		2.15 E-1	6.8	13.5	-0.9798	4.3466	Y	Y	2.89 E-2	3.77 E+6	6.35 E+6	-0.7500	23.2710	Y
PGN1605	*PG0328*	*conserved hypothetical protein*												
PG0352		3.82 E-1	1.7	4	-1.2249	2.5138	Y	G	7.01 E-4	1.85 E+3	1.3 E+6	-9.4527	20.3085	G
PGN1608	*PG0324*	*sialidase putative*												
PG0350		3.49 E-1	24.0	32	-0.4175	5.8063	Y	Y	1.85 E-1	2.15 E+7	2.1 E+7	0.0324	25.3431	Y
PGN1611	*PG0322*	*internalin-related protein*												
PG0348		5.43 E-1	2.6	1.5	0.7751	2.0240	Y	Y	1.83 E-2	6.35 E+5	4.17 E+5	0.6058	20.0047	Y
PGN1613	*PG0320*	*recG ATP-dependent DNA helicase RecG*												
PG0347		3.52 E-1	32.5	24.5	0.4083	5.8333	Y	R	2.31 E-6	1.76 E+7	7.7 E+6	1.1894	24.5901	R
PGN1614	*PG0319*	*galE UDP-glucose 4-epimerase*												
PG0346		5.76 E-2	10.3	2.5	2.0381	3.6744	Y	Y	5.66 E-3	3.04 E+6	1.2 E+6	1.3382	22.0165	Y
PGN1615	*PG0318*	*GTP-binding protein*												
PG0345		3.05 E-1	3.4	1	1.7751	2.1449	Y	R	1.12 E-11	1.39 E+6	1 E+3	10.4407	20.4075	R
PGN1616	*PG0316*	*hypothetical protein PG_0345*												
PG0344		6.54 E-3	6.8	23.5	-1.7795	4.9234	G	Y	8.76 E-3	4.21 E+6	8.04 E+6	-0.9310	23.5464	G
PGN1617	*PG0315*	*purple acid phosphatase*												
PG0343		1.63 E-11	531.4	789.5	-0.5712	10.3673	G	Y	5.6 E-2	2.73 E+8	3.18 E+8	-0.2194	29.1384	G
PGN1618	*PG0314*	*megL methionine gamma-lyase*												
PG0339		4.4 E-1	1.7	3.5	-1.0323	2.3816	Y	Y	5.42 E-2	1.85 E+3	9.95 E+6	-12.3929	23.2469	Y
PGN1622	*PG0313*	*hypothetical protein PG_0339*												
PG0338		4.23 E-1	8.6	12.5	-0.5468	4.3962	Y	Y	1.13 E-1	1.61 E+6	2.02 E+6	-0.3295	21.7905	Y
PGN1623	*PG0312*	*hypothetical protein PG_0338*												
PG0336		5.43 E-1	13.7	11	0.3157	4.6259	Y	R	8.93 E-9	5.17 E+6	2.03 E+6	1.3476	22.7790	R
PGN1625	*PG0311*	*hypothetical protein PG_0336*												

TIGR / *PGN*	*LANL*	PP PPC / Pg Spectral Count *q*-value	PP (Norm)	PPC (Norm)	log_2Ratio	log_2Sum	Regulation		PP PPC/ Pg Protein Intensity *q*-value	PP (Norm)	PPC (Norm)	log_2Ratio	log_2Sum	33277 Consensus
		4.55 E-1	5.1	3	0.7751	3.0240	Y	R	7.82 E-5	1.89 E+6	2.81 E+5	2.7497	21.0498	R
PGN1627														
PG0334		2.66 E-1	8.6	15	-0.8099	4.5581	Y	Y	1.34 E-1	4.2 E+6	3.68 E+6	0.1903	22.9111	Y
PGN1628	*PG0309*	*glycosyl transferase group 2 family protein*												
PG0332		1.89 E-3	80.4	42	0.9374	6.9358	R	R	0	5.58 E+7	2.28 E+7	1.2919	26.2289	R
PGN1630	*PG0307*	*rho transcription termination factor Rho*												
PG0330		6.21 E-2	62.5	89.5	-0.5189	7.2476	Y	Y	1.17 E-1	1.32 E+8	1.49 E+8	-0.1755	28.0687	Y
PGN1631	*PG0306*	*DNA-binding protein histone-like family*												
		3.05 E-1	3.4	1	1.7751	2.1449	Y	R	0	1.77 E+5	1 E+3	7.4705	17.4444	R
PGN1632														
PG0329		2.52 E-1	160.0	185.5	-0.2133	8.4326	Y	Y	3.35 E-3	2.03 E+8	1.48 E+8	0.4601	28.3874	Y
PGN1633	*PG0305*	*formiminotransferase-cyclodeaminase-related protein*												
PG0328		3.82 E-1	41.9	51.5	-0.2967	6.5458	Y	Y	8.61 E-3	1.86 E+7	2.57 E+7	-0.4680	25.3990	Y
PGN1634	*PG0304*	*hutI imidazolonepropionase*												
PG0327		2.78 E-1	12.0	6.5	0.8820	4.2078	Y	R	3.96 E-4	4.29 E+6	2.1 E+6	1.0315	22.6055	R
PGN1635	*PG0303*	*hypothetical protein PG_0327*												
PG0326		4.57 E-3	21.4	48	-1.1660	6.1167	G	G	1.14 E-7	6.69 E+6	1.75 E+7	-1.3890	24.5294	G
PGN1636	*PG0302*	*hypothetical protein PG_0326*												
PG0325		1.96 E-12	100.1	234	-1.2249	8.3842	G	Y	1.66 E-1	5.76 E+7	5.55 E+7	0.0524	26.7537	Y
PGN1637	*PG0301*	*conserved hypothetical protein*												
PG0324		5.87 E-2	118.1	155	-0.3925	8.0932	Y	Y	2.44 E-2	7.46 E+7	9.83 E+7	-0.3986	27.3655	Y
PGN1638	*PG0299*	*hutH histidine ammonia-lyase*												
PG0323		8.6 E-7	30.8	87.5	-1.5062	6.8863	G	Y	1.07 E-2	1.47 E+7	2.63 E+7	-0.8390	25.2897	G
PGN1639	*PG0297*	*conserved hypothetical protein*												
PG0321		7.53 E-2	18.0	7.5	1.2605	4.6707	Y	Y	2.25 E-3	4.9 E+6	2.51 E+6	0.9675	22.8202	Y
PGN1641	*PG0295*	*LAO/AO transport system ATPase*												
PG0320		6 E-1	1.7	2.5	-0.5468	2.0743	Y	G	0	1.85 E+3	1.47 E+6	-9.6371	20.4927	G
PGN1642	*PG0294*	*hypothetical protein PG_0320*												
PG0319		6.36 E-5	57.3	19.5	1.5558	6.2636	R	R	0	4.27 E+7	1.29 E+7	1.7282	25.7267	R
PGN1643	*PG0293*	*hypothetical protein PG_0319*												
PG0317		1.1 E-1	16.3	29	-0.8350	5.5001	Y	Y	2.15 E-2	6.05 E+6	1.64 E+7	-1.4393	24.4218	Y
PGN1645	*PG0291*	*peptidase M49 family*												
PG0316		1.76 E-1	58.2	42	0.4702	6.6465	Y	Y	1.37 E-1	2.92 E+7	2.69 E+7	0.1185	25.7400	Y
PGN1646	*PG0290*	*serS seryl-tRNA synthetase*												

TIGR		PP PPC / Pg Spectral Count						PP PPC/ Pg Protein Intensity						33277
PGN	***LANL***	*q*-value	PP (Norm)	PPC (Norm)	log_2Ratio	log_2Sum	Regulation		*q*-value	PP (Norm)	PPC (Norm)	log_2Ratio	log_2Sum	Consensus
PG0315		1.18 E-1	10.3	3.5	1.5527	3.7832	Y	Y	4.06 E-3	7.8 E+6	2.82 E+6	1.4694	23.3396	Y
PGN1647	*PG0289*	*rpmA ribosomal protein L27*												
PG0314		5.1 E-2	35.9	19	0.9195	5.7797	Y	Y	1.44 E-1	2.74 E+7	2.52 E+7	0.1200	25.6508	Y
PGN1648	*PG0288*	*rplU ribosomal protein L21*												
PG0311		5.89 E-1	1.7	1	0.7751	1.4390	Y	R	0	2.43 E+5	1 E+3	7.9231	17.8949	R
PGN1651	*PG0285*	*glycosyl transferase group 2 family protein*												
PG0310		6 E-1	31.7	35	-0.1447	6.0587	Y	Y	5.72 E-2	1.47 E+7	1.95 E+7	-0.4039	25.0296	Y
PGN1652	*PG0284*	*nitroreductase family protein*												
PG0308		6.69 E-1	1.7	1.5	0.1901	1.6832	Y	G	0	1.85 E+3	6.77 E+6	-11.8360	22.6901	Y
PGN1654	*PG0281*	*electron transport complex RnfABCDGE type A subunit*												
PG0307		4.67 E-3	1.7	13.5	-2.9798	3.9271	Y	G	0	1.85 E+3	3.47 E+5	-7.5502	18.4116	G
PGN1655	*PG0280*	*electron transport complex RnfABCDGE type E subunit*												
PG0306		6.07 E-3	47.9	22	1.1230	6.1276	R	R	5.99 E-12	6.07 E+7	1.51 E+7	2.0103	26.1750	R
PGN1656	*PG0279*	*electron transport complex RnfABCDGE type G subunit*												
PG0305		1.42 E-1	6.8	15	-1.1318	4.4492	Y	Y	1.37 E-3	3.17 E+6	7.51 E+6	-1.2436	23.3486	Y
PGN1657	*PG0278*	*electron transport complex RnfABCDGE type D subunit*												
PG0304		6.69 E-1	50.5	49	0.0430	6.6364	Y	Y	3.2 E-2	3.62 E+7	2.98 E+7	0.2835	25.9755	Y
PGN1658	*PG0277*	*electron transport complex RnfABCDGE type C subunit*												
PG0303		1.37 E-1	30.8	46.5	-0.5941	6.2725	Y	Y	6.66 E-2	9.27 E+6	1.06 E+7	-0.1932	24.2438	Y
PGN1659	*PG0276*	*electron transport complex RnfABCDGE type B subunit*												
PG0302		2.15 E-1	4.3	1	2.0970	2.4001	Y	R	3.82 E-8	1.04 E+6	1 E+3	10.0267	19.9939	R
PGN1660	*PG0275*	*hypothetical protein PG_0302*												
PG0300		4.23 E-1	2.6	1	1.3601	1.8347	Y	R	0	1.55 E+6	1 E+3	10.6017	20.5684	R
PGN1661	*PG0274*	*TPR domain protein*												
PG0296		7 E-1	138.6	139	-0.0040	8.1169	Y	Y	1.21 E-1	9.8 E+7	9.24 E+7	0.0852	27.5048	Y
PGN1666	*PG0272*	*phosphoribosylformylglycinamidine synthase putative*												
PG0295		4.75 E-1	3.4	5.5	-0.6843	3.1575	Y	Y	2.16 E-2	1.74 E+6	1.13 E+6	0.6244	21.4495	Y
PGN1667	*PG0271*	*dprA DNA processing protein DprA putative*												
PG0294		6.69 E-1	1.7	2	-0.2249	1.8919	Y	Y	1.54 E-2	1.85 E+3	4.27 E+5	-7.8513	18.7112	Y
PGN1668	*PG0270*	*glycosyl transferase group 2 family protein*												
PG0293		0	247.3	72.5	1.7701	8.3210	R	R	0	2.68 E+8	7.93 E+7	1.7574	28.3719	R
PGN1670	*PG0269*	*secretion activator protein putative*												
PG0291		2.99 E-1	6.0	11	-0.8770	4.0866	Y	Y	1.29 E-2	3.15 E+6	6.4 E+6	-1.0233	23.1868	Y
PGN1673	*PG0266*	*hypothetical protein PG_0291*												

TIGR		PP PPC / Pg Spectral Count						PP PPC/ Pg Protein Intensity						33277
PGN	LANL	q-value	PP (Norm)	PPC (Norm)	log_2Ratio	log_2Sum	Regulation		q-value	PP (Norm)	PPC (Norm)	log_2Ratio	log_2Sum	Consensus
PG0290		3.55 E-2	25.7	11	1.2226	5.1965	Y	Y	5.41 E-2	1.34 E+7	1.05 E+7	0.3460	24.5100	Y
PGN1674	PG0265	hypothetical protein PG_0290												
PG0289		1.47 E-2	36.8	16.5	1.1570	5.7359	Y	R	9.86 E-10	3.29 E+7	9.4 E+6	1.8084	25.3344	R
PGN1675	PG0264	hypothetical protein PG_0289												
PG0288		2.82 E-1	20.5	29.5	-0.5226	5.6449	Y	Y	7.1 E-2	1.42 E+7	1.7 E+7	-0.2593	24.8989	Y
PGN1676	PG0263	lipoprotein putative												
PG0287		1.55 E-3	1.7	15.5	-3.1791	4.1053	G	G	1.9 E-5	1.85 E+3	3.65 E+6	-10.9460	21.8004	G
PGN1677	PG0262	hypothetical protein PG_0287												
PG0286		4.53 E-1	9.4	13	-0.4659	4.4862	Y	G	2.34 E-4	6.5 E+6	2.07 E+7	-1.6701	24.6974	G
PGN1678	PG0260	hypothetical protein PG_0286												
PG0279		1.24 E-1	80.4	105.5	-0.3914	7.5386	Y	Y	1.4 E-3	4.3 E+7	6.48 E+7	-0.5909	26.6839	Y
PGN1685	PG0251	maeB NADP-dependent malic enzyme												
PG0278		6.69 E-1	1.7	1.5	0.1901	1.6832	Y	G	0	1.85 E+3	6.13 E+5	-8.3706	19.2287	Y
PGN1687	PG0250	hypothetical protein PG_0278												
PG1747		1.21 E-1	4.3	11.5	-1.4265	3.9799	Y	Y	1.39 E-1	4.16 E+6	4.56 E+6	-0.1346	23.0557	Y
PGN1688	PG1523	ribose 5-phosphate isomerase B putative												
PG1748		2.21 E-3	150.6	96.5	0.6421	7.9489	R	R	2.85 E-5	1.33 E+8	8.38 E+7	0.6637	27.6909	R
PGN1689	PG1524	tkt transketolase												
PG1750		1.84 E-1	1.7	6	-1.8099	2.9470	Y	G	2.58 E-16	1.85 E+3	7.03 E+5	-8.5685	19.4260	G
PGN1690	PG1525	alpha-13/4-fucosidase putative												
PG1751		4.4 E-1	1.7	3.5	-1.0323	2.3816	Y	Y	1.07 E-2	1.85 E+3	1.19 E+6	-9.3325	20.1885	Y
PGN1691	PG1526	aminotransferase class V												
PG1753		6.97 E-1	2.6	2.5	0.0381	2.3411	Y	R	0	8.4 E+5	4.51 E+5	0.8957	20.2998	R
PGN1693	PG1527	selD selenide water dikinase												
PG1754		6.14 E-1	43.6	47	-0.1071	6.5020	Y	Y	8.83 E-2	2.17 E+7	2.45 E+7	-0.1754	25.4585	Y
PGN1694	PG1528	conserved domain protein												
PG1755		1.97 E-4	298.6	206	0.5357	8.9791	R	Y	4.09 E-3	5.15 E+8	3.93 E+8	0.3911	29.7569	R
PGN1695	PG1529	fbaB fructose-bisphosphate aldolase class I												
PG1757		5.88 E-10	18.0	81	-2.1724	6.6289	G	G	5.03 E-5	1.05 E+7	6.37 E+7	-2.5968	26.1444	G
PGN1697	PG1530	hypothetical protein PG_1757												
PG1758		2.8 E-1	43.6	32.5	0.4252	6.2506	Y	Y	1.84 E-1	6.29 E+7	6.1 E+7	0.0442	26.8842	Y
PGN1698	PG1531	rpsO ribosomal protein S15												
PG1759		3.05 E-1	6.8	12	-0.8099	4.2361	Y	Y	2.67 E-2	2.78 E+6	5.16 E+6	-0.8929	22.9205	Y
PGN1699	PG1532	adhesion protein putative												

TIGR		PP PPC / Pg Spectral Count							PP PPC/ Pg Protein Intensity					33277
PGN	*LANL*	*q*-value	PP (Norm)	PPC (Norm)	log_2Ratio	log_2Sum	Regulation		*q*-value	PP (Norm)	PPC (Norm)	log_2Ratio	log_2Sum	Consensus
PG1760		4.23 E-1	2.6	1	1.3601	1.8347	Y	R	0	1.09 E+6	1 E+3	10.0844	20.0515	R
PGN1700	*PG1533*	*ABC transporter ATP-binding protein*												
PG1762		0	288.4	87.5	1.7205	8.5540	R	R	0	2.17 E+8	9.17 E+7	1.2400	28.1992	R
PGN1702	*PG1536*	*secDF protein-export membrane protein SecD/protein-export membrane protein SecF*												
PG1763		9.44 E-2	6.0	1	2.5825	2.8052	Y	R	1.74 E-15	1.7 E+6	1 E+3	10.7321	20.6987	R
PGN1703	*PG1537*	*rnc ribonuclease III*												
PG1764		9.32 E-3	288.4	221	0.3838	8.9925	R	Y	1.68 E-2	1.22 E+8	1.55 E+8	-0.3461	28.0478	Y
PGN1704	*PG1538*	*fabF 3-oxoacyl-(acyl-carrier-protein) synthase II*												
PG1765		0	476.6	149.5	1.6726	9.2902	R	Y	1.74 E-1	4.62 E+8	4.8 E+8	-0.0553	29.8123	Y
PGN1705		*acpP acyl carrier protein*												
PG1766		6.69 E-1	10.3	9.5	0.1121	4.3051	Y	Y	8.63 E-2	1.56 E+6	2.12 E+6	-0.4482	21.8113	Y
PGN1706	*PG1539*	*purN phosphoribosylglycinamide formyltransferase*												
PG1767		1.53 E-1	2.6	8	-1.6399	3.4015	Y	Y	8.54 E-2	1.47 E+6	3.02 E+6	-1.0390	22.0988	Y
PGN1707	*PG1540*	*lipoprotein putative*												
PG1768		4.23 E-1	2.6	1	1.3601	1.8347	Y	R	0	8.3 E+5	1 E+3	9.6970	19.6645	R
PGN1708	*PG1541*	*magnesium chelatase subunit D/I family*												
PG1771		1.72 E-2	50.5	26.5	0.9298	6.2665	Y	Y	3.72 E-2	1.48 E+7	1.18 E+7	0.3324	24.6665	Y
PGN1711	*PG1545*	*pheS phenylalanyl-tRNA synthetase alpha subunit*												
PG1772		3.05 E-1	5.1	2	1.3601	2.8347	Y	R	0	4.01 E+5	6 E+4	2.7409	18.8146	R
PGN1712	*PG1547*	*nth endonuclease III*												
PG1774		4.23 E-1	6.8	4	0.7751	3.4390	Y	R	2.64 E-5	3.53 E+6	8.19 E+5	2.1082	22.0530	R
PGN1714	*PG1549*	*mfd transcription-repair coupling factor*												
PG1775		3.31 E-1	26.5	19	0.4814	5.5086	Y	Y	1.08 E-1	1.59 E+7	1.77 E+7	-0.1566	25.0039	Y
PGN1715	*PG1550*	*grpE grpE protein*												
PG1776		4.75 E-1	18.0	14	0.3601	4.9986	Y	Y	6.14 E-2	2.97 E+6	5.53 E+6	-0.8945	23.0197	Y
PGN1716	*PG1552*	*dnaJ dnaJ protein*												
PG1777		6.69 E-1	1.7	2	-0.2249	1.8919	Y	G	0	1.85 E+3	1.44 E+6	-9.6029	20.4585	G
PGN1717	*PG1553*	*conserved hypothetical protein*												
PG1778		2.67 E-1	2.6	6.5	-1.3404	3.1806	Y	Y	2.95 E-2	7.96 E+5	1.32 E+6	-0.7310	21.0145	Y
PGN1718	*PG1554*	*conserved hypothetical protein*												
PG1779		6 E-1	14.5	12.5	0.2187	4.7573	Y	Y	1.73 E-1	7.5 E+6	8.16 E+6	-0.1213	23.9006	Y
PGN1719	*PG1555*	*conserved hypothetical protein*												
PG1780		6 E-1	35.1	38.5	-0.1341	6.2013	Y	Y	9.99 E-3	1.25 E+7	9.06 E+6	0.4639	24.3621	Y
PGN1721	*PG1556*	*bioF-3 8-amino-7-oxononanoate synthase*												

TIGR / PGN	LANL	PP PPC / Pg Spectral Count q-value	PP (Norm)	PPC (Norm)	log_2Ratio	log_2Sum	Regulation (Spectral Count)	Regulation (Protein Intensity)	PP PPC/ Pg Protein Intensity q-value	PP (Norm)	PPC (Norm)	log_2Ratio	log_2Sum	33277 Consensus
PG1781		9.98 E-4	29.1	7.5	1.9557	5.1935	R	R	7.95 E-12	1.14 E+7	2.76 E+6	2.0504	23.7574	R
PGN1722	*PG1557*	*udk uridine kinase*												
PG1782		6 E-1	1.7	2.5	-0.5468	2.0743	Y	G	7.18 E-9	1.85 E+3	3.56 E+5	-7.5890	18.4502	G
PGN1723	*PG1558*	*hypothetical protein PG_1782*												
PG1783		6.69 E-1	1.7	2	-0.2249	1.8919	Y	G	1.77 E-6	1.85 E+3	1.56 E+5	-6.3995	17.2702	G
PGN1724	*PG1559*	*glycosyl transferase group 2 family protein*												
		0	1853.3	3174	-0.7762	12.2956	G	Y	1.25 E-2	2.18 E+9	2.63 E+9	-0.2724	32.1611	G
PGN1728														
PG1842		6.69 E-1	1.7	1.5	0.1901	1.6832	Y	G	0	1.85 E+3	2.57 E+5	-7.1198	17.9838	Y
PGN1729	*PG1604*	*acetyltransferase GNAT family*												
PG1841		3.21 E-1	1.7	4.5	-1.3948	2.6349	Y	G	7.61 E-16	1.85 E+3	6.95 E+5	-8.5525	19.4100	G
PGN1730	*PG1603*	*conserved hypothetical protein*												
PG1837		2.09 E-2	77.9	48	0.6979	6.9757	Y	Y	3.34 E-3	1.04 E+8	6.39 E+7	0.7084	27.3255	Y
PGN1733	*PG1602*	*hemagglutinin protein HagA*												
PG1835		4.76 E-1	30.8	36.5	-0.2448	6.0726	Y	Y	5.08 E-3	2.04 E+7	3.37 E+7	-0.7238	25.6890	Y
PGN1735	*PG1600*	*lipoprotein putative*												
PG1834		4.21 E-1	29.9	23.5	0.3498	5.7401	Y	Y	6.16 E-2	1.45 E+7	1.2 E+7	0.2748	24.6555	Y
PGN1736	*PG1598*	*glycogen synthase-related protein*												
PG1831		6.69 E-1	1.7	1.5	0.1901	1.6832	Y	G	0	1.85 E+3	9.39 E+5	-8.9877	19.8443	Y
PGN1737	*PG1597*	*recQ-2 ATP-dependent DNA helicase RecQ*												
PG1829		2.32 E-1	8.6	15.5	-0.8572	4.5884	Y	G	2.72 E-4	1.26 E+6	5.01 E+6	-1.9951	22.5787	G
PGN1738	*PG1596*	*long-chain-fatty-acid--CoA ligase putative*												
PG1824		1.69 E-6	463.8	635.5	-0.4545	10.1023	G	G	1.36 E-5	2.91 E+8	4.26 E+8	-0.5510	29.4182	G
PGN1743	*PG1593*	*eno enolase*												
PG1823		5.13 E-5	1.7	21.5	-3.6512	4.5368	G	G	5.96 E-5	1.85 E+3	1.06 E+7	-12.4779	23.3318	G
PGN1744	*PG1592*	*hypothetical protein PG_1823*												
PG1820		6.69 E-1	1.7	2	-0.2249	1.8919	Y	G	2.69 E-9	1.85 E+3	2.24 E+6	-10.2441	21.0990	G
PGN1746	*PG1591*	*nrfA cytochrome c nitrite reductase catalytic subunit NrfA*												
PG1816		1.07 E-2	103.5	65.5	0.6605	7.4012	Y	Y	1.74 E-1	4.47 E+7	4.31 E+7	0.0518	26.3878	Y
PGN1749	*PG1587*	*NAD(P)H dehydrogenase quinone family putative*												
PG1815		6.52 E-2	6.0	16	-1.4175	4.4587	Y	G	1.55 E-5	8.89 E+5	4.94 E+6	-2.4733	22.4738	G
PGN1750	*PG1586*	*kdsB 3-deoxy-D-manno-octulosonate cytidylyltransferase*												
PG1814		1.86 E-1	6.8	14	-1.0323	4.3816	Y	Y	1.88 E-1	2.49 E+6	2.54 E+6	-0.0270	22.2619	Y
PGN1751	*PG1585*	*dnaG DNA primase*												

TIGR		PP PPC / Pg Spectral Count							PP PPC/ Pg Protein Intensity					33277
PGN	***LANL***	*q*-value	PP (Norm)	PPC (Norm)	log_2Ratio	log_2Sum	Regulation		*q*-value	PP (Norm)	PPC (Norm)	log_2Ratio	log_2Sum	Consensus
PG1813		1.84 E-1	2.6	7.5	-1.5468	3.3316	Y	Y	3.72 E-2	2.92 E+6	5.2 E+6	-0.8333	22.9524	Y
PGN1752		*ferredoxin 4Fe-4S*												
PG1812		0	651.2	321.5	1.0182	9.9258	R	R	0	4.33 E+8	1.88 E+8	1.1999	29.2111	R
PGN1753	*PG1583*	*2-oxoglutarate oxidoreductase alpha subunit*												
PG1810		2.35 E-7	155.7	72.5	1.1030	7.8343	R	R	4.17 E-10	1.19 E+8	4.89 E+7	1.2811	27.3205	R
PGN1755	*PG1582*	*2-oxoglutarate oxidoreductase beta subunit*												
PG1809		4.35 E-12	116.4	30.5	1.9318	7.1984	R	R	0	1.35 E+8	2.8 E+7	2.2716	27.2798	R
PGN1756	*PG1581*	*2-oxoglutarate oxidoreductase gamma subunit*												
PG1808		4.75 E-1	23.1	18.5	0.3205	5.3786	Y	Y	2.01 E-1	7.87 E+6	7.85 E+6	0.0028	23.9057	Y
PGN1757	*PG1580*	*spoT guanosine-3'5'-bis(diphosphate) 3'-pyrophosphohydrolase*												
PG1806		6.34 E-3	29.1	10	1.5406	5.2888	R	R	3.54 E-4	1.68 E+7	7.04 E+6	1.2539	24.5072	R
PGN1759	*PG1578*	*atpI v-type ATPase subunit I*												
PG1805		2.62 E-1	18.8	28	-0.5728	5.5492	Y	Y	1.45 E-1	8.02 E+6	8.65 E+6	-0.1089	23.9901	Y
PGN1760	*PG1577*	*atpD v-type ATPase subunit D*												
PG1804		5.28 E-6	246.4	369	-0.5825	9.2654	G	Y	1.78 E-1	1.42 E+8	1.38 E+8	0.0350	28.0599	Y
PGN1761	*PG1576*	*atpB v-type ATPase subunit B*												
PG1803		5.89 E-1	159.2	151.5	0.0711	8.2792	Y	Y	7.53 E-2	1.15 E+8	1.29 E+8	-0.1625	27.8589	Y
PGN1762	*PG1575*	*atpA v-type ATPase subunit A*												
PG1802		7.01 E-1	52.2	52	0.0054	6.7031	Y	Y	1.32 E-2	2.15 E+7	1.71 E+7	0.3277	25.2027	Y
PGN1763	*PG1574*	*hypothetical protein PG_1802*												
PG1801		5.11 E-6	58.2	16.5	1.8182	6.2227	R	R	0	4.85 E+7	6.74 E+6	2.8455	25.7181	R
PGN1764	*PG1573*	*v-type ATPase subunit E putative*												
PG1798		1.15 E-2	35.9	65	-0.8550	6.6573	Y	Y	8.87 E-3	1.49 E+7	2.21 E+7	-0.5742	25.1420	Y
PGN1767	*PG1572*	*immunoreactive 46 kDa antigen PG99*												
PG1797		6.05 E-2	16.3	6	1.4381	4.4762	Y	R	4.42 E-7	7.99 E+6	3.03 E+6	1.4007	23.3931	R
PGN1768	*PG1571*	*DNA-binding response regulator/sensor histidine kinase*												
PG1794		3.82 E-1	1.7	4	-1.2249	2.5138	Y	G	2.88 E-6	1.85 E+3	1.94 E+6	-10.0368	20.8919	G
PGN1771	*PG1569*	*polA DNA polymerase type I*												
PG1793		6.69 E-1	10.3	11	-0.0994	4.4106	Y	Y	3.49 E-3	4.2 E+6	2.26 E+6	0.8921	22.6223	Y
PGN1772	*PG1568*	*glgB 14-alpha-glucan branching enzyme*												
PG1792		4.23 E-1	2.6	1	1.3601	1.8347	Y	R	0	1.63 E+5	1 E+3	7.3492	17.3238	R
PGN1773	*PG1567*	*sodium/hydrogen antiporter*												
PG1790		1.5 E-1	1.7	6.5	-1.9253	3.0376	Y	G	6.92 E-6	1.85 E+3	1.12 E+6	-9.2375	20.0936	G
PGN1775	*PG1566*	*hypothetical protein PG_1790*												

TIGR		PP PPC / Pg Spectral Count						PP PPC/ Pg Protein Intensity						33277
PGN	***LANL***	*q*-value	PP (Norm)	PPC (Norm)	log_2Ratio	log_2Sum	Regulation		*q*-value	PP (Norm)	PPC (Norm)	log_2Ratio	log_2Sum	Consensus
PG1789		2.8 E-2	21.4	41.5	-0.9561	5.9748	Y	Y	1.12 E-3	1.08 E+7	2.08 E+7	-0.9478	24.9126	Y
PGN1776	*PG1565*	*dcp-2 peptidyl-dipeptidase Dcp*												
PG1788		6.11 E-3	283.2	213.5	0.4077	8.9563	R	Y	2.02 E-1	2.81 E+8	2.81 E+8	0.0006	29.0672	R
PGN1777	*PG1564*	*cysteine peptidase putative*												
PG1847		8.08 E-7	24.8	77.5	-1.6430	6.6769	G	G	1.82 E-5	1.17 E+7	2.51 E+7	-1.1049	25.1325	G
PGN1780	*PG1609*	*endoribonuclease L-PSP putative*												
PG1848		3.05 E-1	5.1	2	1.3601	2.8347	Y	R	0	2.85 E+6	1.68 E+5	4.0800	21.5248	R
PGN1781	*PG1610*	*RNA methyltransferase TrmH family*												
PG1849		6.69 E-1	2.6	3	-0.2249	2.4769	Y	Y	3.41 E-2	1.15 E+6	6.07 E+5	0.9172	20.7418	Y
PGN1782	*PG1611*	*recN DNA repair protein RecN*												
PG1850		3.52 E-1	10.3	6	0.7751	4.0240	Y	R	0	3.07 E+6	7.52 E+5	2.0324	21.8674	R
PGN1783	*PG1612*	*hypothetical protein PG_1850*												
PG1851		3.9 E-1	9.4	14	-0.5728	4.5492	Y	Y	1.3 E-2	4.08 E+6	8.88 E+6	-1.1205	23.6281	Y
PGN1784	*PG1613*	*coaBC phosphopantothenoylcysteine decarboxylase/phosphopantothenate--cysteine ligase*												
PG1852		1.38 E-2	38.5	17.5	1.1377	5.8075	Y	Y	1.27 E-3	1.33 E+7	7.12 E+6	0.8990	24.2820	Y
PGN1785	*PG1614*	*exonuclease*												
PG1853		3.23 E-2	85.6	56	0.6116	7.1453	Y	Y	1.96 E-1	4.13 E+7	4.1 E+7	0.0111	26.2943	Y
PGN1786	*PG1615*	*dnaN DNA polymerase III beta subunit*												
PG1854		6.69 E-1	1.7	1.5	0.1901	1.6832	Y	G	0	1.85 E+3	5.7 E+5	-8.2658	19.1242	Y
PGN1787	*PG1616*	*5-formyltetrahydrofolate cyclo-ligase family protein*												
PG1855		2.25 E-1	5.1	1.5	1.7751	2.7299	Y	R	0	3.86 E+6	1.12 E+6	1.7911	22.2471	R
PGN1788	*PG1617*	*carboxyl-terminal protease*												
PG1858		6.45 E-2	37.6	59	-0.6481	6.5947	Y	Y	6.64 E-2	2.43 E+7	3.44 E+7	-0.4981	25.8068	Y
PGN1791	*PG1621*	*flavodoxin*												
PG1860		2.15 E-1	4.3	1	2.0970	2.4001	Y	R	5.11 E-12	8.07 E+5	1 E+3	9.6568	19.6243	R
PGN1793	*PG1624*	*conserved hypothetical protein*												
PG1862		6.69 E-1	3.4	3	0.1901	2.6832	Y	Y	1.83 E-1	9.54 E+5	1.03 E+6	-0.1114	20.9201	Y
PGN1795	*PG1625*	*hypothetical protein PG_1862*												
PG1864		6.94 E-1	18.8	18.5	0.0251	5.2220	Y	R	3.3 E-4	4.28 E+6	2.5 E+6	0.7760	22.6925	R
PGN1796	*PG1626*	*leucine-rich protein*												
PG1872		7.07 E-13	394.5	636.5	-0.6903	10.0098	G	G	9.75 E-6	1.46 E+8	2.73 E+8	-0.9079	28.6427	G
PGN1800	*PG1630*	*hutU urocanate hydratase*												
PG1875		6.69 E-1	1.7	1.5	0.1901	1.6832	Y	G	0	1.85 E+3	1.39 E+5	-6.2340	17.1067	Y
PGN1802	*PG1632*	*hemolysin*												

TIGR	PGN	LANL	Description	PP PPC / Pg Spectral Count q-value	PP (Norm)	PPC (Norm)	log_2Ratio	log_2Sum	Regulation (Spectral Count)	Regulation (Protein Intensity)	PP PPC/ Pg Protein Intensity q-value	PP (Norm)	PPC (Norm)	log_2Ratio	log_2Sum	33277 Consensus
PG1876	PGN1803	PG1633	*conserved hypothetical protein*	1.5 E-1	1.7	6.5	-1.9253	3.0376	Y	G	1.37 E-4	1.85 E+3	1.39 E+6	-9.5564	20.4121	G
PG1877	PGN1804	PG1634	*nhaA Na+/H+ antiporter*	6.69 E-1	1.7	1.5	0.1901	1.6832	Y	G	0	1.85 E+3	5.5 E+5	-8.2152	19.0738	Y
PG1878	PGN1805	PG1635	*cysS cysteinyl-tRNA synthetase*	1.85 E-3	54.8	24	1.1901	6.2994	R	Y	1.69 E-3	1.52 E+7	9.87 E+6	0.6228	24.5792	R
PG1879	PGN1806	PG1636	*conserved hypothetical protein*	5.8 E-1	26.5	30	-0.1776	5.8208	Y	Y	2.52 E-3	1.78 E+7	2.97 E+7	-0.7373	25.5040	Y
PG1880	PGN1807	PG1637	*glycosyl transferase group 2 family protein*	5.17 E-1	3.4	2	0.7751	2.4390	Y	R	0	9.4 E+5	8.86 E+5	0.0842	20.8003	R
PG1881	PGN1808	PG1638	*hypothetical protein PG_1881*	1.67 E-12	2.6	52.5	-4.3542	5.7831	G	G	0	8.22 E+5	3.81 E+7	-5.5332	25.2133	G
PG1884	PGN1811	PG1639	*alpha-L-fucosidase precursor putative*	4.53 E-1	25.7	20.5	0.3244	5.5289	Y	R	4.19 E-4	1.03 E+7	5.24 E+6	0.9701	23.8866	R
PG1885	PGN1812	PG1640	*ppk polyphosphate kinase*	8.79 E-3	24.0	7.5	1.6756	4.9754	R	R	1.33 E-12	1.03 E+7	1.64 E+6	2.6500	23.5097	R
PG1886	PGN1813	PG1641	*hflX GTP-binding protein HflX*	6.69 E-1	6.8	7.5	-0.1318	3.8425	Y	Y	1.89 E-1	2.01 E+6	2.07 E+6	-0.0384	21.9587	Y
PG1887	PGN1814	PG1642	*rhodanese-like domain protein*	6.69 E-1	3.4	3	0.1901	2.6832	Y	R	3.16 E-6	1.1 E+6	2.6 E+5	2.0811	20.3726	R
PG1888	PGN1815	PG1643	*conserved hypothetical protein*	3.82 E-1	1.7	4	-1.2249	2.5138	Y	G	1.22 E-9	1.85 E+3	4.85 E+5	-8.0350	18.8942	G
PG1889	PGN1816	PG1644	*hypothetical protein PG_1889*	5.72 E-1	47.1	42.5	0.1471	6.4848	Y	R	9.56 E-9	1.54 E+7	8.17 E+6	0.9182	24.4928	R
	PGN1818			5.89 E-1	1.7	1	0.7751	1.4390	Y	R	0	1.27 E+5	1 E+3	6.9893	16.9664	R
	PGN1819			5.95 E-1	12.0	10	0.2605	4.4581	Y	Y	1.32 E-2	3.33 E+6	2.1 E+6	0.6660	22.3726	Y
PG1896	PGN1827	PG1652	*metK S-adenosylmethionine synthase*	1.15 E-3	55.6	101	-0.8607	7.2911	G	G	1.68 E-6	3.16 E+7	6.11 E+7	-0.9491	26.4668	G
PG1897	PGN1828	PG1653	*thiamin pyrophosphokinase catalytic domain protein*	2.74 E-1	1.7	5	-1.5468	2.7466	Y	G	3.47 E-4	1.85 E+3	1.85 E+6	-9.9666	20.8217	G
PG1899	PGN1830	PG1655	*TonB-dependent receptor putative*	2.31 E-4	77.9	137.5	-0.8204	7.7506	G	G	1.83 E-4	5.56 E+7	1.05 E+8	-0.9110	27.2558	G

TIGR	PGN	LANL	Description	PP PPC / Pg Spectral Count q-value	PP (Norm)	PPC (Norm)	log_2Ratio	log_2Sum	Regulation		PP PPC/ Pg Protein Intensity q-value	PP (Norm)	PPC (Norm)	log_2Ratio	log_2Sum	33277 Consensus
PG1901	PGN1832	PG1657	*frr ribosome recycling factor*	2.34 E-3	47.9	20	1.2605	6.0857	R	R	7.06 E-14	7.89 E+7	1.82 E+7	2.1140	26.5335	R
PG1902	PGN1833	PG1658	*pyrH uridylate kinase*	4.55 E-1	5.1	3	0.7751	3.0240	Y	Y	1.72 E-1	3.1 E+6	2.86 E+6	0.1149	22.5077	Y
PG1903	PGN1834	PG1659	*conserved hypothetical protein*	1.21 E-2	24.8	8.5	1.5456	5.0581	Y	R	8.73 E-11	6.18 E+6	1.89 E+6	1.7087	22.9443	R
PG1910	PGN1840	PG1666	*rplQ ribosomal protein L17*	1.02 E-5	103.5	45.5	1.1862	7.2195	R	Y	5.07 E-3	7.64 E+7	4.94 E+7	0.6281	26.9063	R
PG1911	PGN1841	PG1667	*rpoA DNA-directed RNA polymerase alpha subunit*	4.13 E-4	145.5	86	0.7582	7.8546	R	R	9.81 E-4	7.47 E+7	3.66 E+7	1.0271	26.7298	R
PG1912	PGN1842	PG1668	*rpsD ribosomal protein S4*	1.04 E-4	149.7	84.5	0.8254	7.8718	R	R	1.04 E-13	1.72 E+8	6.79 E+7	1.3391	27.8356	R
PG1913	PGN1843	PG1669	*rpsK ribosomal protein S11*	2.11 E-4	39.4	11	1.8392	5.6542	R	Y	9.55 E-3	1.82 E+7	1.03 E+7	0.8198	24.7664	R
PG1914	PGN1844	PG1670	*rpsM ribosomal protein S13*	5.43 E-1	31.7	27.5	0.2032	5.8865	Y	Y	1.22 E-2	3.33 E+7	1.96 E+7	0.7668	25.6579	Y
PG1916	PGN1846		*infA translation initiation factor IF-1*	5.43 E-1	15.4	12.5	0.3012	4.8023	Y	R	3.62 E-6	5.91 E+6	2.1 E+6	1.4957	22.9327	R
PG1917	PGN1847	PG1672	*map methionine aminopeptidase type I*	2.6 E-2	10.3	25.5	-1.3124	5.1606	Y	Y	1.87 E-1	4.59 E+6	4.46 E+6	0.0439	23.1094	Y
PG1918	PGN1848	PG1673	*secY preprotein translocase SecY subunit*	4.75 E-1	25.7	31	-0.2722	5.8245	Y	Y	2.1 E-2	2.89 E+7	1.88 E+7	0.6214	25.5060	Y
PG1919	PGN1849	PG1673	*rplO ribosomal protein L15*	4.58 E-5	62.5	22	1.5055	6.4002	R	R	2.58 E-16	3.49 E+7	1.05 E+7	1.7296	25.4383	R
PG1920	PGN1850		*rpmD ribosomal protein L30*	1.94 E-2	40.2	19.5	1.0443	5.9000	Y	R	2.03 E-6	4.75 E+7	1.85 E+7	1.3615	25.9756	R
PG1921	PGN1851	PG1674	*rpsE ribosomal protein S5*	2.82 E-2	40.2	20.5	0.9721	5.9240	Y	Y	1.72 E-2	4.76 E+7	2.71 E+7	0.8133	26.1544	Y
PG1922	PGN1852	PG1675	*rplR ribosomal protein L18*	6.85 E-1	24.0	24.5	-0.0323	5.5987	Y	Y	9.2 E-2	2.9 E+7	2.38 E+7	0.2876	25.6533	Y
PG1923	PGN1853	PG1676	*rplF ribosomal protein L6*	4.37 E-1	151.5	166	-0.1323	8.3104	Y	Y	1.69 E-1	1.52 E+8	1.58 E+8	-0.0542	28.2041	Y
PG1924	PGN1854	PG1677	*rpsH ribosomal protein S8*	5.89 E-2	24.8	11.5	1.1095	5.1825	Y	Y	1.83 E-1	3.49 E+7	3.3 E+7	0.0811	26.0167	Y

TIGR		PP PPC / Pg Spectral Count							PP PPC/ Pg Protein Intensity					33277
PGN	*LANL*	*q*-value	PP (Norm)	PPC (Norm)	log_2Ratio	log_2Sum	Regulation		*q*-value	PP (Norm)	PPC (Norm)	log_2Ratio	log_2Sum	Consensus
PG1925		6.69 E-1	1.7	1.5	0.1901	1.6832	Y	G	0	1.85 E+3	2.5 E+6	-10.4023	21.2571	Y
PGN1855	*PG1678*	*rpsN ribosomal protein S14*												
PG1926		4.87 E-11	313.2	162.5	0.9465	8.8938	R	Y	1.23 E-1	1.12 E+8	1.03 E+8	0.1220	27.6845	R
PGN1856	*PG1679*	*rplE ribosomal protein L5*												
PG1927		7.93 E-2	26.5	13.5	0.9744	5.3228	Y	R	7.88 E-5	3.36 E+7	1.54 E+7	1.1269	25.5464	R
PGN1857	*PG1680*	*rplX ribosomal protein L24*												
PG1928		2.31 E-15	95.8	14	2.7751	6.7792	R	R	3.44 E-15	4.27 E+7	1.08 E+7	1.9854	25.6710	R
PGN1858	*PG1681*	*rplN ribosomal protein L14*												
PG1929		2.15 E-3	12.8	1	3.6820	3.7902	Y	R	3.24 E-7	6.65 E+6	1 E+3	12.7000	22.6660	R
PGN1859	*PG1682*	*rpsQ ribosomal protein S17*												
PG1930		6.05 E-2	6.8	1	2.7751	2.9718	Y	R	4.06 E-6	9.46 E+6	1 E+3	13.2080	23.1740	R
PGN1860		*rpmC ribosomal protein L29*												
PG1931		6.69 E-1	13.7	13	0.0747	4.7383	Y	Y	1.45 E-1	1.89 E+7	1.75 E+7	0.1098	25.1143	Y
PGN1861	*PG1683*	*rplP ribosomal protein L16*												
PG1932		3.3 E-4	107.8	57	0.9195	7.3647	R	Y	2.88 E-3	7.26 E+7	4.93 E+7	0.5598	26.8614	R
PGN1862	*PG1684*	*rpsC ribosomal protein S3*												
PG1933		2.15 E-1	27.4	17.5	0.6458	5.4880	Y	Y	7.62 E-2	1.61 E+7	1.2 E+7	0.4212	24.7430	Y
PGN1863	*PG1685*	*rplV ribosomal protein L22*												
PG1934		1.74 E-3	24.0	5.5	2.1230	4.8806	R	R	2.61 E-14	2.12 E+7	1.98 E+6	3.4177	24.4654	R
PGN1864	*PG1686*	*rpsS ribosomal protein S19*												
PG1935		3.16 E-3	27.4	8	1.7751	5.1449	R	R	0	3.13 E+7	3.6 E+6	3.1205	25.0551	R
PGN1865	*PG1687*	*rplB ribosomal protein L2*												
PG1936		2.27 E-2	11.1	2	2.4755	3.7141	Y	R	0	4.1 E+6	6.89 E+5	2.5744	22.1923	R
PGN1866	*PG1688*	*rplW ribosomal protein L23*												
PG1937		0	177.1	28	2.6612	7.6803	R	R	0	1.11 E+8	1.55 E+7	2.8445	26.9177	R
PGN1867	*PG1689*	*rplD ribosomal protein L4*												
PG1938		7.31 E-2	93.3	66.5	0.4880	7.3198	Y	Y	1.16 E-2	7.4 E+7	5.08 E+7	0.5411	26.8949	Y
PGN1868	*PG1690*	*rplC ribosomal protein L3*												
PG1939		4.02 E-4	129.2	73.5	0.8138	7.6632	R	Y	2.48 E-3	8.12 E+7	4.64 E+7	0.8072	26.9266	R
PGN1869	*PG1691*	*rpsJ ribosomal protein S10*												
PG1940		0	634.0	89	2.8327	9.4979	R	R	0	4.8 E+8	8.95 E+7	2.4232	29.0853	R
PGN1870	*PG1692*	*fusA translation elongation factor G*												
PG1941		6.66 E-3	62.5	32.5	0.9426	6.5693	R	R	1.16 E-6	7.28 E+7	3.2 E+7	1.1844	26.6436	R
PGN1871	*PG1693*	*rpsG ribosomal protein S7*												

TIGR			PP PPC / Pg Spectral Count							PP PPC/ Pg Protein Intensity						33277
PGN	LANL		*q*-value	PP (Norm)	PPC (Norm)	log_2Ratio	log_2Sum	Regulation		*q*-value	PP (Norm)	PPC (Norm)	log_2Ratio	log_2Sum		Consensus
PG1942			3.05 E-1	3.4	1	1.7751	2.1449	Y	Y	1.42 E-3	2.77 E+6	1 E+3	11.4345	21.4008		Y
PGN1872	*PG1694*		*rpsL ribosomal protein S12*													
PG1947			4.41 E-1	10.3	7	0.5527	4.1100	Y	Y	1.38 E-1	2.46 E+6	2.19 E+6	0.1699	22.1497		Y
PGN1877	*PG1699*		*TPR domain protein*													
PG1948			3.82 E-3	169.4	235.5	-0.4751	8.6615	G	Y	1.01 E-2	1.2 E+8	1.54 E+8	-0.3531	28.0301		G
PGN1878	*PG1700*		*lipoprotein putative*													
PG1949			2.8 E-2	206.2	157.5	0.3888	8.5067	Y	Y	2.72 E-2	1.34 E+8	1.94 E+8	-0.5320	28.2922		Y
PGN1880	*PG1701*		*mdh malate dehydrogenase*													
PG1950			1.86 E-1	5.1	11.5	-1.1635	4.0561	Y	Y	9.67 E-3	2.33 E+6	3.73 E+6	-0.6799	22.5295		Y
PGN1882	*PG1703*		*membrane protein putative*													
PG1951			3.78 E-2	71.0	103	-0.5364	7.4431	Y	Y	1.66 E-3	4.21 E+7	6.4 E+7	-0.6040	26.6603		Y
PGN1883	*PG1704*		*glnS glutaminyl-tRNA synthetase*													
PG1952			7.06 E-1	6.0	6	-0.0025	3.5837	Y	R	1.24 E-4	6.07 E+6	2.84 E+6	1.0939	23.0865		Y
PGN1884	*PG1705*		*DedA family protein*													
PG1953			3.88 E-1	31.7	24.5	0.3698	5.8114	Y	Y	1.89 E-1	1.83 E+7	1.87 E+7	-0.0316	25.1399		Y
PGN1885	*PG1706*		*YitT family protein*													
PG1956			2.02 E-1	51.3	37	0.4725	6.4650	Y	R	4.32 E-4	2.39 E+7	1.47 E+7	0.7033	25.2024		R
PGN1888	*PG1708*		*abfT-2 4-hydroxybutyrate CoA-transferase*													
			4.23 E-1	2.6	1	1.3601	1.8347	Y	R	0	2.99 E+6	1 E+3	11.5467	21.5130		R
PGN1889																
PG1959			6.69 E-1	1.7	1.5	0.1901	1.6832	Y	G	0	1.85 E+3	1.37 E+5	-6.2097	17.0828		Y
PGN1890	*PG1710*		*rpmG ribosomal protein L33*													
PG1960			3.68 E-1	11.1	16.5	-0.5689	4.7878	Y	Y	2.16 E-2	1.02 E+7	5.91 E+6	0.7878	23.9421		Y
PGN1891	*PG1710*		*rpmB ribosomal protein L28*													
PG1961			6.97 E-1	2.6	2.5	0.0381	2.3411	Y	Y	1.11 E-1	9.09 E+5	7.22 E+5	0.3328	20.6366		Y
PGN1892	*PG1711*		*hypothetical protein PG_1961*													
			3.82 E-1	1.7	4	-1.2249	2.5138	Y	G	2.11 E-6	1.85 E+3	9.67 E+5	-9.0300	19.8865		G
PGN1893																
PG1964			6.69 E-1	1.7	2	-0.2249	1.8919	Y	Y	1.27 E-2	1.85 E+3	5.84 E+5	-8.3009	19.1592		Y
PGN1896	*PG1715*		*bacterial sugar transferase*													
PG1966			5.17 E-1	1.7	3	-0.8099	2.2361	Y	Y	1.52 E-3	1.85 E+3	1.34 E+6	-9.5025	20.3582		Y
PGN1898	*PG1717*		*conserved hypothetical protein*													
			1.73 E-1	31.7	46	-0.5390	6.2791	Y	G	1.01 E-5	1.45 E+7	3.09 E+7	-1.0891	25.4379		G
PGN1903																

TIGR	PGN	LANL	Description	PP PPC / Pg Spectral Count q-value	PP (Norm)	PPC (Norm)	log_2Ratio	log_2Sum	Regulation (Spectral Count)	Regulation (Protein Intensity)	PP PPC/ Pg Protein Intensity q-value	PP (Norm)	PPC (Norm)	log_2Ratio	log_2Sum	33277 Consensus
PG1972	PGN1904	PG1721	hemagglutinin protein HagB	5.8 E-4	200.2	284.5	-0.5068	8.9210	G	Y	2.76 E-3	2.86 E+8	3.98 E+8	-0.4743	29.3488	G
PG1977	PGN1907	PG1726	hypothetical protein PG_1977	2.74 E-1	1.7	5	-1.5468	2.7466	Y	Y	2.02 E-2	1.85 E+3	5.18 E+5	-8.1292	18.9881	Y
	PGN1910			4.23 E-1	2.6	1	1.3601	1.8347	Y	R	0	1.69 E+6	1 E+3	10.7218	20.6885	R
PG1983	PGN1928	PG1731	CRISPR-associated protein TM1791 family	3.05 E-1	18.0	11.5	0.6439	4.8811	Y	Y	3.02 E-2	1.01 E+7	6.9 E+6	0.5486	24.0194	Y
PG1984	PGN1929	PG1732	hypothetical protein PG_1984	3.37 E-1	12.0	18	-0.5875	4.9059	Y	Y	1.38 E-1	4.57 E+6	5.11 E+6	-0.1624	23.2062	Y
PG1985	PGN1930	PG1733	CRISPR-associated protein TM1792 family	3.85 E-1	15.4	10.5	0.5527	4.6950	Y	Y	3.98 E-2	1.09 E+7	1.56 E+7	-0.5164	24.6620	Y
PG1986	PGN1931	PG1734	CRISPR-associated protein TM1793 family	4.93 E-1	18.8	15	0.3276	5.0800	Y	R	3.28 E-9	6.75 E+6	2.66 E+6	1.3424	23.1665	R
PG1987	PGN1932	PG1735	CRISPR-associated protein TM1811 family	2.8 E-1	11.1	18	-0.6944	4.8641	Y	Y	7.67 E-3	3.07 E+6	5.6 E+6	-0.8691	23.0470	Y
PG1988	PGN1933	PG1736	hypothetical protein PG_1988	5.43 E-1	16.3	19.5	-0.2624	5.1602	Y	Y	1.08 E-1	7.7 E+6	6.38 E+6	0.2708	23.7471	Y
	PGN1935			7.43 E-2	6.8	17	-1.3124	4.5756	Y	Y	8.87 E-3	1.87 E+6	3.06 E+6	-0.7107	22.2341	Y
PG1992	PGN1937	PG1739	gidA glucose-inhibited division protein A	4.21 E-1	3.4	1.5	1.1901	2.2994	Y	R	1.36 E-6	1.74 E+6	4.18 E+5	2.0619	21.0436	R
PG1993	PGN1938	PG1740	uvrC excinuclease ABC C subunit	6.69 E-1	1.7	1.5	0.1901	1.6832	Y	G	0	1.85 E+3	1.19 E+5	-6.0095	16.8854	Y
PG1994	PGN1939	PG1741	dtd D-tyrosyl-tRNA(Tyr) deacylase	2.93 E-1	6.8	3	1.1901	3.2994	Y	R	2.63 E-5	5.2 E+6	1.82 E+6	1.5127	22.7447	R
PG1996	PGN1941	PG1743	deoC deoxyribose-phosphate aldolase	2.66 E-1	68.5	85	-0.3124	7.2616	Y	Y	3.62 E-2	3.48 E+7	5.47 E+7	-0.6539	26.4147	Y
PG1998	PGN1943	PG1744	polyprenyl synthetase	1.06 E-1	20.5	10	1.0381	4.9324	Y	R	7.32 E-7	7.75 E+6	3.21 E+6	1.2693	23.3858	R
PG2001	PGN1946	PG1747	lepB signal peptidase I	4.4 E-1	18.0	13.5	0.4125	4.9758	Y	R	8.78 E-6	1.31 E+7	6.45 E+6	1.0257	24.2222	R
PG2002	PGN1947	PG1748	dapB dihydrodipicolinate reductase	3.99 E-1	17.1	23	-0.4265	5.3260	Y	Y	4.24 E-2	5.19 E+6	6.92 E+6	-0.4146	23.5297	Y

TIGR	PGN	LANL	Description	PP PPC / Pg Spectral Count q-value	PP (Norm)	PPC (Norm)	log_2Ratio	log_2Sum	Regulation	PP PPC/ Pg Protein Intensity Regulation	q-value	PP (Norm)	PPC (Norm)	log_2Ratio	log_2Sum	33277 Consensus
PG2003	PGN1948	PG1749	dgt deoxyguanosinetriphosphate triphosphohydrolase	4.02 E-1	7.7	4.5	0.7751	3.6089	Y	Y	7.74 E-2	1.82 E+6	2.56 E+6	-0.4959	22.0617	Y
PG2008	PGN1953	PG1752	TonB-dependent receptor putative	1.63 E-3	17.1	44.5	-1.3787	5.9452	G	G	1.2 E-8	5.14 E+6	2.13 E+7	-2.0541	24.6585	G
PG2010	PGN1955	PG1754	phosphomannomutase putative	2.93 E-1	111.2	93.5	0.2506	7.6776	Y	R	4.24 E-5	7.65 E+7	4.96 E+7	0.6241	26.9096	R
PG2014	PGN1960	PG1758	cas1 CRISPR-associated protein Cas1	5.8 E-1	7.7	6	0.3601	3.7762	Y	Y	1.74 E-3	1.62 E+6	8.9 E+5	0.8655	21.2597	Y
PG2015	PGN1961	PG1759	cas4 CRISPR-associated protein Cas4	6.69 E-1	1.7	2	-0.2249	1.8919	Y	Y	1.08 E-3	1.85 E+3	2.66 E+5	-7.1660	18.0297	Y
	PGN1962			1.09 E-1	269.5	227	0.2478	8.9557	Y	R	3.5 E-6	1.36 E+8	9.16 E+7	0.5745	27.7647	R
	PGN1963			6.69 E-1	68.5	70.5	-0.0425	7.1184	Y	Y	2.04 E-2	4.56 E+7	5.75 E+7	-0.3362	26.6197	Y
PG2016	PGN1964	PG1760	cas3 CRISPR-associated helicase Cas3	1.73 E-2	1.7	11	-2.6843	3.6680	Y	Y	2.64 E-3	1.85 E+3	3.54 E+6	-10.9034	21.7579	Y
	PGN1965			3.29 E-1	10.3	16	-0.6399	4.7152	Y	Y	2.53 E-3	5.97 E+6	9.77 E+6	-0.7114	23.9081	Y
PG2020	PGN1966	PG1764	CRISPR-associated protein TM1814 family	3.78 E-1	25.7	19	0.4341	5.4812	Y	Y	1.01 E-1	1.9 E+7	1.62 E+7	0.2370	25.0692	Y
PG2021	PGN1967	PG1765	conserved hypothetical protein	6 E-1	1.7	2.5	-0.5468	2.0743	Y	Y	1.14 E-3	1.85 E+3	4.79 E+5	-8.0154	18.8746	Y
PG2022	PGN1968	PG1766	hypothetical protein PG_2022	6.49 E-1	17.1	15.5	0.1428	5.0274	Y	Y	3.76 E-2	8.55 E+6	6.51 E+6	0.3942	23.8441	Y
PG2023	PGN1969	PG1767	fmt methionyl-tRNA formyltransferase	1.57 E-2	40.2	19	1.0818	5.8879	Y	R	1.87 E-7	1.46 E+7	5.87 E+6	1.3153	24.2883	R
	PGN1970			4.89 E-2	519.4	597	-0.2009	10.1246	Y	Y	2.79 E-2	4.61 E+8	5.62 E+8	-0.2876	29.9298	Y
PG2026	PGN1973	PG1769	phosphoglycerate mutase family protein	2.25 E-1	1.7	5.5	-1.6843	2.8503	Y	Y	4.02 E-3	1.85 E+3	2.71 E+6	-10.5186	21.3733	Y
PG2029	PGN1976	PG1772	hypothetical protein PG_2029	4.23 E-1	191.7	209	-0.1249	8.6463	Y	Y	4.45 E-2	1.7 E+8	1.9 E+8	-0.1638	28.4251	Y
PG2031	PGN1978	PG1774	hypothetical protein PG_2031	6.41 E-1	6.0	5	0.2605	3.4581	Y	Y	6.16 E-2	1.21 E+6	7.16 E+5	0.7569	20.8780	Y

TIGR		PP PPC / Pg Spectral Count							PP PPC/ Pg Protein Intensity					33277
PGN	*LANL*	*q*-value	PP (Norm)	PPC (Norm)	log_2Ratio	log_2Sum	Regulation		*q*-value	PP (Norm)	PPC (Norm)	log_2Ratio	log_2Sum	Consensus
PG2033		4.93 E-1	8.6	11.5	-0.4265	4.3260	Y	Y	1.01 E-1	3.49 E+6	2.79 E+6	0.3218	22.5826	Y
PGN1980	*PG1776*	*gltD glutamate synthase small subunit*												
PG2034		2.92 E-1	31.7	22.5	0.4927	5.7591	Y	Y	1.52 E-1	1.09 E+7	1.23 E+7	-0.1780	24.4691	Y
PGN1981	*PG1777*	*oxidoreductase FAD-binding putative*												
PG2036		2.15 E-1	4.3	1	2.0970	2.4001	Y	Y	2.02 E-3	5.73 E+6	1 E+3	12.4843	22.4504	Y
PGN1983	*PG1779*	*ion transporter*												
PG2040		3.82 E-1	1.7	4	-1.2249	2.5138	Y	G	4.25 E-5	1.85 E+3	3.36 E+6	-10.8246	21.6791	G
PGN1986	*PG1781*	*DNA-binding protein histone-like family*												
PG2041		6.05 E-2	1.7	8.5	-2.3124	3.3521	Y	G	9.91 E-15	1.85 E+3	1.94 E+6	-10.0330	20.8881	G
PGN1987	*PG1782*	*hypothetical protein PG_2041*												
PG2042		3.93 E-1	4.3	7.5	-0.8099	3.5581	Y	G	8.96 E-4	1.94 E+6	3.33 E+6	-0.7795	22.3293	G
PGN1988	*PG1783*	*thioredoxin family protein*												
PG2043		1.45 E-1	40.2	26	0.6292	6.0491	Y	Y	3.61 E-2	8.2 E+6	5.92 E+6	0.4701	23.7510	Y
PGN1989	*PG1784*	*conserved hypothetical protein TIGR00486*												
PG2044		8.62 E-3	49.6	24	1.0481	6.2022	R	R	6.97 E-4	2.88 E+7	1.58 E+7	0.8676	25.4109	R
PGN1990	*PG1785*	*conserved hypothetical protein*												
PG2050		1.98 E-2	57.3	32	0.8412	6.4811	Y	Y	1.94 E-1	6.9 E+7	6.79 E+7	0.0240	27.0280	Y
PGN1995	*PG1790*	*hypothetical protein PG_2050*												
PG2052		6.41 E-1	16.3	18	-0.1469	5.0983	Y	Y	1.74 E-1	1.35 E+7	1.41 E+7	-0.0636	24.7139	Y
PGN1996	*PG1791*	*dapA dihydrodipicolinate synthase*												
PG2053		5.43 E-1	2.6	1.5	0.7751	2.0240	Y	R	0	1.25 E+6	1.08 E+6	0.2073	21.1561	R
PGN1997	*PG1792*	*bioD dethiobiotin synthase*												
PG2054		6.94 E-1	35.1	35.5	-0.0171	6.1412	Y	Y	7.15 E-2	2.78 E+7	3.69 E+7	-0.4078	25.9486	Y
PGN1998	*PG1793*	*lipoprotein PG3*												
PG2055		6.74 E-1	178.0	176	0.0161	8.4675	Y	R	1.26 E-6	9.21 E+7	5.63 E+7	0.7112	27.1448	R
PGN1999	*PG1794*	*dihydroorotate dehydrogenase family protein*												
PG0052		3.05 E-1	3.4	1	1.7751	2.1449	Y	R	0	1.14 E+6	1 E+3	10.1491	20.1161	R
PGN2001	*PG0045*	*sensor histidine kinase*												
PG0054		4.53 E-1	9.4	13	-0.4659	4.4862	Y	Y	1.26 E-2	2.46 E+6	7.54 E+6	-1.6130	23.2541	Y
PGN2003	*PG0046*	*recJ single-stranded-DNA-specific exonuclease RecJ*												
PG0055		1.06 E-1	6.8	1.5	2.1901	3.0610	Y	R	3.44 E-15	1.46 E+6	7.78 E+4	4.2320	20.5534	R
PGN2004	*PG0047*	*conserved domain protein*												
PG0056		5.89 E-1	83.0	77.5	0.0989	7.3264	Y	Y	1.5 E-1	3.04 E+7	3.87 E+7	-0.3479	26.0430	Y
PGN2005	*PG0048*	*hypothetical protein PG_0056*												

TIGR		PP PPC / Pg Spectral Count							PP PPC/ Pg Protein Intensity					33277
PGN	***LANL***	*q*-value	PP (Norm)	PPC (Norm)	log_2Ratio	log_2Sum	Regulation		*q*-value	PP (Norm)	PPC (Norm)	log_2Ratio	log_2Sum	Consensus
PG0057		7.53 E-2	18.0	7.5	1.2605	4.6707	Y	R	4.94 E-6	3.6 E+6	1.72 E+6	1.0612	22.3439	R
PGN2006	*PG0049*	*pncB nicotinate phosphoribosyltransferase*												
PG0058		6 E-1	1.7	2.5	-0.5468	2.0743	Y	Y	3.03 E-2	2.92 E+5	3.83 E+5	-0.3949	19.3648	Y
PGN2007	*PG0050*	*nadD nicotinate (nicotinamide) nucleotide adenylyltransferase*												
PG0059		2.15 E-1	4.3	1	2.0970	2.4001	Y	R	0	2.4 E+6	1 E+3	11.2306	21.1970	R
PGN2008	*PG0051*	*hypothetical protein PG_0059*												
PG0062		5.89 E-1	1.7	1	0.7751	1.4390	Y	R	0	1.77 E+5	1 E+3	7.4711	17.4450	R
PGN2011	*PG0053*	*TPR domain protein*												
PG0063		3.21 E-1	1.7	4.5	-1.3948	2.6349	Y	Y	2.02 E-2	1.37 E+6	3.77 E+6	-1.4570	22.2950	Y
PGN2012	*PG0054*	*outer membrane efflux protein*												
PG0064		3.55 E-1	8.6	13.5	-0.6579	4.4631	Y	Y	2.6 E-2	2.19 E+6	3.66 E+6	-0.7364	22.4803	Y
PGN2013	*PG0055*	*heavy metal efflux pump CzcA family*												
PG0065		5.89 E-1	1.7	1	0.7751	1.4390	Y	R	0	2.52 E+5	1 E+3	7.9797	17.9511	R
PGN2014	*PG0056*	*efflux transporter RND family MFP subunit*												
PG0069		6.28 E-3	11.1	31	-1.4787	5.3966	G	G	1.49 E-6	2.8 E+6	6.57 E+6	-1.2277	23.1600	G
PGN2017	*PG0058*	*conserved hypothetical protein*												
PG0070		8.78 E-3	82.1	48	0.7751	7.0240	R	Y	1.6 E-1	4.76 E+7	4.45 E+7	0.0974	26.4556	R
PGN2018	*PG0059*	*lpxA acyl-(acyl-carrier-protein)-UDP-N-acetylglucosamine acyltransferase*												
PG0071		3.37 E-1	123.2	106.5	0.2103	7.8437	Y	Y	1.38 E-1	8.89 E+7	8.37 E+7	0.0873	27.3629	Y
PGN2019	*PG0060*	*UDP-3-O-acyl-GlcNAc deacetylase/beta-hydroxyacyl-[acyl carrier protein] dehydratase FabZ*												
PG0072		1.21 E-3	40.2	14	1.5223	5.7606	R	R	3.24 E-7	1.75 E+7	5.53 E+6	1.6592	24.4552	R
PGN2020	*PG0061*	*lpxD UDP-3-O-[3-hydroxymyristoyl] glucosamine N-acyltransferase*												
PG0073		3.2 E-6	24.8	74	-1.5764	6.6266	G	G	1.08 E-8	1.63 E+7	5.34 E+7	-1.7128	26.0547	G
PGN2021	*PG0062*	*orotidine 5'-monophosphate decarboxylase*												
PG0074		6.05 E-2	6.8	1	2.7751	2.9718	Y	R	2.58 E-16	4.07 E+6	1 E+3	11.9904	21.9566	R
PGN2022	*PG0063*	*prfA peptide chain release factor 1*												
PG0075		2.04 E-4	31.7	7	2.1772	5.2727	R	R	0	8.78 E+6	2.08 E+6	2.0772	23.3731	R
PGN2023	*PG0064*	*phosphoribosylformylglycinamidine cyclo-ligase putative*												
PG0076		5.8 E-1	7.7	6	0.3601	3.7762	Y	Y	1.17 E-2	4.61 E+6	2.97 E+6	0.6343	22.8552	Y
PGN2024	*PG0065*	*N-acetylmuramoyl-L-alanine amidase family 4*												
PG0078		6 E-1	1.7	2.5	-0.5468	2.0743	Y	G	9.28 E-6	1.85 E+3	4.9 E+5	-8.0485	18.9077	G
PGN2025	*PG0066*	*conserved hypothetical protein*												
PG0079		1.16 E-1	29.1	16.5	0.8182	5.5107	Y	R	1.34 E-6	2.24 E+7	2.86 E+6	2.9678	24.5875	R
PGN2026	*PG0067*	*abortive infection protein putative*												

TIGR		PP PPC / Pg Spectral Count						PP PPC/ Pg Protein Intensity						33277
PGN	*LANL*	*q*-value	PP (Norm)	PPC (Norm)	log_2Ratio	log_2Sum	Regulation		*q*-value	PP (Norm)	PPC (Norm)	log_2Ratio	log_2Sum	Consensus
PG0081		3.21 E-1	1.7	4.5	-1.3948	2.6349	Y	Y	4.63 E-2	1.85 E+3	2.67 E+6	-10.4953	21.3501	Y
PGN2028	*PG0069*	*hypothetical protein PG_0081*												
PG0082		2.84 E-2	1.7	10	-2.5468	3.5498	Y	G	8.43 E-6	1.85 E+3	2.31 E+6	-10.2863	21.1412	G
PGN2029	*PG0070*	*hypothetical protein PG_0082*												
PG0083		1.84 E-1	1.7	6	-1.8099	2.9470	Y	G	1.22 E-5	1.85 E+3	2.21 E+6	-10.2189	21.0739	G
PGN2030	*PG0071*	*hypothetical protein PG_0083*												
PG0084		4.03 E-1	5.1	8.5	-0.7274	3.7691	Y	Y	6.1 E-3	1.23 E+6	2.86 E+6	-1.2224	21.9616	Y
PGN2031	*PG0072*	*sda L-serine dehydratase iron-sulfur-dependent single chain form*												
PG0086		1.24 E-1	24.8	13.5	0.8782	5.2598	Y	Y	2.79 E-2	1.01 E+7	6.53 E+6	0.6313	23.9894	Y
PGN2033	*PG0074*	*ATP-dependent RNA helicase DEAD/DEAH box family*												
PG0087		5.43 E-1	4.3	6	-0.4879	3.3615	Y	Y	1.84 E-1	2.92 E+6	3.05 E+6	-0.0637	22.5082	Y
PGN2034	*PG0076*	*SIS domain protein*												
PG0090		2.77 E-9	543.3	354	0.6181	9.8095	R	Y	4.61 E-2	6.15 E+8	5.27 E+8	0.2236	30.0893	R
PGN2037	*PG0079*	*Dps family protein*												
PG0091		6.69 E-1	1.7	2	-0.2249	1.8919	Y	Y	1.37 E-3	1.85 E+3	4.37 E+5	-7.8821	18.7419	Y
PGN2038	*PG0080*	*transporter putative*												
PG0092		3.05 E-1	3.4	1	1.7751	2.1449	Y	R	9.6 E-14	9.06 E+5	1 E+3	9.8241	19.7915	R
PGN2039	*PG0081*	*transporter putative*												
PG0093		5.94 E-2	11.1	3	1.8906	3.8200	Y	Y	2.98 E-3	4.75 E+6	1.62 E+6	1.5514	22.6038	Y
PGN2040	*PG0082*	*HlyD family secretion protein*												
PG0094		2.09 E-3	23.1	53	-1.1979	6.2499	G	G	2.79 E-4	9.59 E+6	3.26 E+7	-1.7666	25.3318	G
PGN2041	*PG0083*	*outer membrane efflux protein putative*												
PG0095		4.23 E-1	11.1	7.5	0.5686	4.2191	Y	Y	2.1 E-2	3.47 E+6	2.26 E+6	0.6164	22.4492	Y
PGN2042	*PG0084*	*mutS DNA mismatch repair protein MutS*												
PG0097		1.9 E-12	95.0	19	2.3216	6.8326	R	R	0	1.93 E+7	1.9 E+6	3.3446	24.3371	R
PGN2043	*PG0085*	*conserved hypothetical protein TIGR01033*												
PG0099		7.94 E-4	78.7	38.5	1.0319	6.8731	R	R	1.56 E-14	4.42 E+7	1.92 E+7	1.2013	25.9196	R
PGN2045	*PG0086*	*pheT phenylalanyl-tRNA synthetase beta subunit*												
PG2072		2.15 E-1	4.3	1	2.0970	2.4001	Y	R	5.72 E-6	1.88 E+6	1 E+3	10.8767	20.8432	R
PGN2050	*PG1811*	*UvrD/REP helicase domain protein*												
PG2070		3.64 E-1	5.1	9	-0.8099	3.8211	Y	Y	3.45 E-2	1.05 E+6	2.02 E+6	-0.9389	21.5508	Y
PGN2052	*PG1809*	*hypothetical protein PG_2070*												
PG2069		2.52 E-1	19.7	12	0.7137	4.9855	Y	R	9.57 E-4	9.71 E+6	4.67 E+6	1.0576	23.7775	R
PGN2053	*PG1808*	*oxidoreductase short chain dehydrogenase/reductase family*												

TIGR		PP PPC / Pg Spectral Count							PP PPC/ Pg Protein Intensity					33277
PGN	*LANL*	*q*-value	PP (Norm)	PPC (Norm)	log_2Ratio	log_2Sum	Regulation		*q*-value	PP (Norm)	PPC (Norm)	log_2Ratio	log_2Sum	Consensus
PG2068		1.61 E-1	46.2	31.5	0.5527	6.2799	Y	Y	1.36 E-1	2.92 E+7	3.29 E+7	-0.1717	25.8884	Y
PGN2054	*PG1807*	*tagD glycerol-3-phosphate cytidylyltransferase*												
PG2067		4.23 E-1	20.5	26.5	-0.3679	5.5557	Y	Y	2.07 E-2	7.97 E+6	1.19 E+7	-0.5766	24.2429	Y
PGN2055	*PG1806*	*pdxA pyridoxal phosphate biosynthetic protein PdxA*												
PG2066		2.09 E-1	41.1	28.5	0.5272	6.1204	Y	Y	1.42 E-3	1.95 E+7	1.25 E+7	0.6433	24.9290	Y
PGN2056	*PG1805*	*lipoprotein putative*												
PG2065		6.69 E-1	2.6	3	-0.2249	2.4769	Y	G	0	4.9 E+5	9.16 E+5	-0.9037	20.4230	G
PGN2057	*PG1804*	*conserved hypothetical protein TIGR00048*												
PG2062		2 E-2	39.4	19	1.0507	5.8669	Y	R	2.01 E-7	2.07 E+7	10 E+6	1.0484	24.8705	R
PGN2060	*PG1801*	*hisS histidyl-tRNA synthetase*												
PG2061		5.43 E-1	3.4	5	-0.5468	3.0743	Y	R	5.28 E-4	1.25 E+6	7.29 E+5	0.7782	20.9166	Y
PGN2061	*PG1800*	*folA dihydrofolate reductase*												
PG2060		7.06 E-1	18.0	18	-0.0025	5.1687	Y	Y	1.07 E-2	9.94 E+6	6.34 E+6	0.6493	23.9569	Y
PGN2062	*PG1799*	*thyA thymidylate synthase*												
PG2197		5.74 E-1	4.3	3	0.5121	2.8636	Y	R	1.77 E-4	1.44 E+6	1.47 E+5	3.2877	20.5953	R
PGN2064	*PG1921*	*conserved hypothetical protein*												
PG2024		4.23 E-1	3.4	6	-0.8099	3.2361	Y	Y	6.09 E-3	1.45 E+7	1.79 E+6	3.0116	23.9537	Y
PGN2065	*PG1768*	*hagE hemagglutinin protein HagE*												
PG2199		4.02 E-1	4.3	2	1.0970	2.6504	Y	R	0	1.44 E+6	3.97 E+5	1.8628	20.8118	R
PGN2066	*PG1923*	*ABC transporter ATP-binding protein putative*												
PG2200		3.06 E-1	4.3	1.5	1.5121	2.5306	Y	R	0	1.33 E+6	1.86 E+5	2.8385	20.5338	R
PGN2067	*PG1924*	*TPR domain protein*												
PG2201		6.49 E-1	15.4	17	-0.1424	5.0180	Y	Y	1.74 E-1	4.65 E+6	4.88 E+6	-0.0699	23.1830	Y
PGN2068	*PG1925*	*def polypeptide deformylase*												
PG2204		1.55 E-3	63.3	110	-0.7968	7.4373	G	Y	7.49 E-3	3.52 E+7	4.58 E+7	-0.3799	26.2723	G
PGN2070	*PG1927*	*hypothetical protein PG_2204*												
PG2205		3.76 E-1	16.3	22.5	-0.4688	5.2764	Y	Y	3.53 E-2	7.93 E+6	1.03 E+7	-0.3817	24.1220	Y
PGN2071	*PG1928*	*panE 2-dehydropantoate 2-reductase*												
PG2206		2.52 E-4	29.1	6	2.2776	5.1331	R	R	0	1.39 E+7	2.66 E+6	2.3868	23.9838	R
PGN2072	*PG1929*	*ABC transporter ATP-binding protein*												
PG2207		2.62 E-2	71.0	43.5	0.7072	6.8394	Y	Y	1.67 E-3	5.2 E+7	3.01 E+7	0.7881	26.2909	Y
PGN2073	*PG1931*	*conserved domain protein*												
PG2210		4.75 E-1	14.5	11	0.4031	4.6750	Y	R	6.76 E-5	4.1 E+6	1.82 E+6	1.1718	22.4971	R
PGN2075	*PG1934*	*uvrA-2 excinuclease ABC A subunit*												

TIGR		PP PPC / Pg Spectral Count						PP PPC/ Pg Protein Intensity						33277
PGN	*LANL*	*q*-value	PP (Norm)	PPC (Norm)	log_2Ratio	log_2Sum	Regulation		*q*-value	PP (Norm)	PPC (Norm)	log_2Ratio	log_2Sum	Consensus
PG2215		7.2 E-3	43.6	19.5	1.1621	5.9804	R	R	3.64 E-4	4.38 E+7	2.29 E+7	0.9377	25.9920	R
PGN2079	*PG1936*	*manC mannose-1-phosphate guanylyltransferase*												
PG2216		5.77 E-2	26.5	45.5	-0.7785	6.1704	Y	Y	1.11 E-3	1.47 E+7	2.14 E+7	-0.5369	25.1055	Y
PGN2080	*PG1938*	*hypothetical protein PG_2216*												
PG2217		4.23 E-1	17.1	12.5	0.4532	4.8882	Y	Y	1.13 E-1	7.09 E+6	5.96 E+6	0.2511	23.6374	Y
PGN2081	*PG1939*	*dxs deoxyxylulose-5-phosphate synthase*												
PG2218		4.75 E-1	3.4	5.5	-0.6843	3.1575	Y	Y	2.23 E-2	1.98 E+6	1.38 E+6	0.5149	21.6807	Y
PGN2082	*PG1940*	*trkA potassium uptake protein TrkA*												
PG2221		6.46 E-4	1.7	17	-3.3124	4.2258	G	G	8.33 E-7	1.85 E+3	3.01 E+6	-10.6698	21.5244	G
PGN2085	*PG1942*	*MiaB-like tRNA modifying enzyme*												
PG2222		6.97 E-1	3.4	3.5	-0.0323	2.7913	Y	R	1.05 E-5	2.63 E+6	6.66 E+5	1.9787	21.6506	Y
PGN2086	*PG1943*	*acyltransferase HtrB/MsbB family*												
PG2223		6.69 E-1	1.7	2	-0.2249	1.8919	Y	G	0	1.85 E+3	9.43 E+4	-5.6705	16.5523	G
PGN2087	*PG1944*	*glycosyl transferase group 2 family protein*												
PG2227		6.77 E-1	4.3	4	0.0970	3.0493	Y	Y	1.29 E-1	2.33 E+6	2.7 E+6	-0.2109	22.2629	Y
PGN2091		*hypothetical protein PG_2227*												
